# Heterogeneity of Powdery Mildew Resistance Revealed in Accessions of the ICARDA Wild Barley Collection

**DOI:** 10.3389/fpls.2017.00202

**Published:** 2017-02-14

**Authors:** Antonin Dreiseitl

**Affiliations:** Department of Integrated Plant Protection, Agrotest Fyto Ltd, KroměřížCzechia

**Keywords:** *Blumeria graminis* f. sp. *hordei*, diversity of resistances, *Hordeum vulgare* subsp. *spontaneum*, phenotypes, response type arrays

## Abstract

The primary genepool of barley comprises two subspecies – wild barley (*Hordeum vulgare* subsp. *spontaneum*) and cultivated barley *H. vulgare.* subsp. *vulgare.* The former originated 5.5 million years ago in southwest Asia and is the immediate ancestor of cultivated barley, which arose around 10,000 years ago. In this study, the specific resistance of a set of 146 wild barley accessions, maintained by the International Center for Agriculture Research in the Dry Areas (ICARDA), to 32 isolates of barley powdery mildew caused by *Blumeria graminis* f. sp. *hordei* was evaluated. The set comprised 146 heterogeneous accessions of a previously tested collection. Seed was obtained by single seed descent and each accession was usually represented by five single plant progenies. In total, 687 plant progenies were tested. There were 211 phenotypes of resistance among the accessions, 87 of which were found in single plants, while 202 plants contained the eight most common phenotypes. The most frequent phenotype was found in 56 plants that were susceptible to all pathogen isolates, whereas the second most frequent phenotype, which occurred in 46 plants, was resistant to all isolates. The broad resistance diversity that was revealed is of practical importance and is an aid to determining the extent and role of resistance in natural ecosystems.

## Introduction

The grass family, Poaceae, originated during the Upper Cretaceous period and the *Hordeum* and *Triticum* genera diverged about 13 million years ago followed by the evolution of *Hordeum* one million years later (Nevo, 2013). More than 30 species of barley (*Hordeum*) are known, nearly all of which are included in the tertiary genepool of *H. vulgare* L. The secondary genepool comprises *H. bulbosum* L., which shares the basic genome with *H. vulgare*. Although there are interspecific crossability problems, especially regarding the tertiary genepool (Bothmer et al., 2003), discrete introgressions from *H. bulbosum* into the *H. vulgare* genome have been successful resulting in the transfer of disease resistance and other useful agronomic traits (Pickering et al., 2006; Shtaya et al., 2007; Wendler et al., 2015).

The primary genepool of barley is composed of two subspecies namely, wild barley [*H. vulgare* subsp. *spontaneum* (C. Koch) Tell.] and cultivated barley (*H. vulgare* subsp. *vulgare*) (hereafter denoted by *Hvs* and *Hvv*, respectively). *H. vulgare* originated 5.5 million years ago in southwest Asia (Dai et al., 2012). *Hvv* first arose from *Hvs* by independent polyphyletic domestication in three centres (Middle-East, Central Asia, and Tibet) around 10,000 years ago (Nevo, 2013). *Hvs* is two-rowed and diploid (2*n* = 2*x* = 14) and differs from *Hvv* in several important traits including brittle rachis and a tough (non-brittle) awn. There are no crossability barriers between the two subspecies and gene transfer is easily accomplished. The centre of distribution for *Hvs* is located mainly in southwest Asia, particularly in the Middle-East where vertical zonality and the diversity of environmental and climatic factors led to ecological and morphological differentiation (Bothmer et al., 2003).

Powdery mildew caused by *Blumeria graminis* (D.C.) Golovin ex Speer f. sp. *hordei* Em. Marchal (*Bgh*) is a world-wide disease of barley (Jørgensen, 1994; Dreiseitl, 2011a) infecting both *Hvv* and *Hvs*. Many host resistances to *Bgh* have been found in *Hvs* from the Middle-East (Fischbeck et al., 1976; Dreiseitl and Bockelman, 2003). Therefore, the region can be considered as a “specific” centre of diversity of barley resistance to *Bgh*. Regular occurrence of the pathogen (Dinoor and Eshed, 1990) and the long-term influence of evolutionary forces (McDonald and Linde, 2002) in the Middle-Eastern population of *Bgh* is indicated by the presence of many corresponding virulences (Eyal et al., 1973; Dreiseitl et al., 2006). Thus, the Middle-Eastern population of *Bgh* on *Hvs* is also the natural centre of diversity of this pathogen (Dreiseitl, 2014).

*Hvs* is still abundant in regions where it occurs naturally and because of its great diversity of useful traits for crop breeding there are also collections in gene banks. The resistance of *Hvs* against powdery mildew is usually done with a set of *Bgh* isolates. Isolate pathogenicity is determined using host genotypes that can differentiate the pathogen isolates. In both cases, the results are collated as response type arrays (RTAs) consisting of response types (RT = phenotype of plant × isolate interaction). The objective of this study was to determine the presence of specific resistances to *Bgh* in a set of heterogeneous *Hvs* accessions held in the International Center for Agriculture Research in the Dry Areas (ICARDA).

## Materials and Methods

### Plant Material and Pathogen Isolates

Resistance of a set of 146 accessions of *Hvs* maintained by ICARDA was studied (**Table 1**). This set comprised accessions from 363 previously tested accessions whose heterogeneity prevented a definitive assessment of RTAs (Dreiseitl, unpublished). Seed was obtained using single seed descent and progenies from five single plants were used for screening 128 accessions. The remaining 18 accessions had fewer plants available for testing. In total, 687 plant progenies were tested at the Agrotest Fyto Ltd.

**Table 1:** Origin of 146 *Hordeum vulgare* subsp. *spontaneum* accessions maintained by the International Center for Agriculture Research in the Dry Areas (ICARDA) tested with 32 *Blumeria graminis* f. sp. *hordei* isolates.

38612	SYR	39504	SYR	39999	JOR	40189	LBN	120796	UZB
38615	SYR	39505	SYR	40001	JOR	40190	LBN	120797	UZB
38622	JOR	39510	SYR	40003	JOR	40192	LBN	120798	UZB
38624	JOR	39513	SYR	40006	JOR	110734	SYR	120799	KAZ
38628	JOR	39515	SYR	40007	JOR	110736	SYR	123975	UZB
38629	JOR	39516	SYR	40011	JOR	110759	SYR	126414	TKM
38636	SYR	39530	SYR	40013	JOR	110762	SYR	126433	TKM
38639	SYR	39541	LBN	40017	JOR	110771	SYR	131685	TJK
39409	SYR	39842	SYR	40018	JOR	110772	SYR	135239	JOR
39411	SYR	39844	SYR	40025	JOR	110823	LBN	135244	JOR
39412	SYR	39845	SYR	40030	JOR	115784	JOR	135249	JOR
39415	SYR	39851	JOR	40032	JOR	115785	JOR	135270	JOR
39421	SYR	39855	SYR	40036	JOR	115795	JOR	135279	JOR
39441	SYR	39856	SYR	40038	JOR	116097	TUR	135294	JOR
39443	SYR	39881	SYR	40040	JOR	116103	TUR	135333	JOR
39450	SYR	39921	SYR	40042	JOR	116109	TUR	135351	SYR
39468	SYR	39925	KAZ	40044	JOR	116110	TUR	135461	TKM
39477	SYR	39943	SYR	40048	JOR	116113	TUR	135474	TKM
39478	SYR	39944	SYR	40066	JOR	116115	TUR	135489	TKM
39480	SYR	39959	SYR	40076	JOR	116117	TUR	135536	TKM
39482	SYR	39984	JOR	40095	SYR	116123	TUR	135553	TKM
39484	SYR	39985	JOR	40096	SYR	116131	TUR	135682	JOR
39488	SYR	39986	JOR	40160	SYR	117888	SYR	135762	JOR
39490	SYR	39987	JOR	40163	SYR	117892	SYR	135777	JOR
39491	SYR	39991	JOR	40165	SYR	119406	SYR	135808	JOR
39495	SYR	39992	JOR	40168	LBN	119407	SYR	135897	JOR
39497	SYR	39993	JOR	40169	SYR	119413	SYR		
39499	SYR	39994	JOR	40172	SYR	119447	SYR		
39500	SYR	39997	JOR	40176	SYR	120790	TKM		
39503	SYR	39998	JOR	40185	LBN	120792	TKM		

*JOR, Jordan (50); KAZ, Kazakhstan (2); LBN, Lebanon (7); SYR, Syria (64); TJK, Tajikistan (1); TKM, Turkmenistan (9); TUR, Turkey (9); UZB, Uzbekistan (4). The 18 highlighted accessions had fewer than five plant progenies tested.*

Thirty-two reference isolates of *Bgh* from the pathogen gene bank at Agrotest Fyto Ltd. representing the world diversity of the pathogen were used for resistance tests (**Table 2**). Five of them were collected on *Hvs* naturally occurring in Israel. Before inoculation, each isolate was purified, verified as the correct pathogenicity phenotype on standard barley lines (Kølster et al., 1986) and multiplied on leaf segments of susceptible B-3213.

**Table 2:** Origin of 32 *Blumeria graminis* f. sp. *hordei* isolates used for resistance tests of 687 *Hordeum vulgare* subsp. *spontaneum* plant progenies and their virulence (+) against 13 *Ml* resistance genes.

No.	Isolate^∗^	*Ml* resistance genes											
		*a1*	*a3*	*a6*	*a7*	*a9*	*a12*	*a13*	*k1*	*La*	*g*	*at*	*Ru2*	*Lo*
1	A-GH/05								+		+	+		+
2	CH-3-33/03												+	+
3	J-Race I/b55	+								+			+	
4	JAR-65/04								+					+
5	U-54/05								+	+	+			+
6	DK-R86/b93		+	+						+				+
7	G-120/b99	+										+		+
8	CR-21/97	+		+	+	+	+	+			+		+	+
9	CR-39/99	+		+	+	+	+		+	+	+		+	+
10	CR-248/99	+	+		+	+	+	+	+	+	+	+	+	+
11	CR-512/01			+	+	+	+		+	+	+			+
12	CR-72/07	+	+	+	+	+	+	+	+	+	+	+	+	+
13	CR-109/08			+	+	+	+			+	+		+	+
14	CR-23/09			+	+	+	+	+	+	+	+	+		+
15	CR-45/09			+	+		+	+			+	+	+	+
16	CR-158/09			+	+			+					+	+
17	CR-162/09			+	+	+	+		+		+		+	+
18	CR-167/09	+		+	+	+	+	+	+	+			+	+
19	CR-184/09		+		+		+			+	+		+	+
20	CR-190/09	+		+	+	+	+	+	+	+	+			+
21	CR-198/09		+	+	**+**		+		+	+	+		+	+
22	CR-200/09	+	+		+	+	+	+	+	+	+		+	+
23	CR-209/09	+		+	+	+	+	+	+	+	+		+	+
24	CR-232/09	+		+	+	+	+		+		+		+	+
25	CR-236/09	+	+	+			+			+	+		+	+
26	CR-246/09	+	+	+	+		+	+		+	+		+	+
27	CR-283/09			+	+		+	+		+	+	+		+
28	I-35/79	+		+		+	+		+	+	+	+	+	+
29	I-69/79	+	+	+			+		+	+	+	+	+	+
30	I-148/79	+		+		+	+		+	+	+	+	+	+
31	I-462/79	+	+	+			+		+	+	+	+	+	
32	I-16/97	+		+			+		+	+		+	+	

*^∗^A, Australia; CH, China; CR, Czech Republic; DK, Denmark; G, Germany; I, Israel; J, Japan; JAR, Republic of South Africa; U, Uruguay. Numbers after a slash indicates the year of isolate collection and b, before the year.*

### Testing Procedure

About 30 seeds of each barley plant were sown in a pot (80 mm diameter) filled with a gardening peat substrate and placed in a mildew-proof greenhouse under natural daylight. Leaf segments 20 mm long were cut from the central part of healthy fully expanded primary leaves when the second leaves were emerging. Three segments of each plant and four segments of the susceptible B-3213 were placed with adaxial surfaces upward in a 150 mm Petri dish on water agar (0.8%) containing benzimidazole (40 mg/l) – a leaf senescence inhibitor. For each isolate, a dish with leaf segments was placed at the bottom of a metal inoculation tower and inoculated at a concentration of *c*. 8 conidiospores/mm^2^. The dishes with inoculated leaf segments were incubated at 18 ± 2.0°C under artificial light (cool-white fluorescent lamps providing 12 h light at 30 ± 5 μmol/m^2^/s).

### Evaluations and Plant Phenotype Designations

Seven days after inoculation, RTs on the central part of the adaxial side of leaf segments were scored on a scale 0–4, where 0 = no visible mycelium or sporulation, and 4 = strong mycelial growth and sporulation on the leaf segment (Torp et al., 1978); RTs 0–2 were considered resistant. In rare cases, when slightly different RTs occurred on the three leaf segments the prevalent RT was recorded. A set of RTs provided a RTA for each *Hvs* plant. Based on the gene-for-gene model (Flor, 1971), a specific resistance gene of the host is matched by a virulence gene of the pathogen.

Numerical designations of *Hvs* phenotypes (RTAs) were based on their resistance/susceptibility patterns to the set of 32 isolates ranked in the order shown in **Table 2** and divided into 10 triplets and two last isolates on their own. Each of the digits indicates susceptibility to the three isolates of the respective triplet. If susceptibility to a corresponding isolate was detected, the first isolate is given the value 1 (2^0^), the second isolate has the value 2 (2^1^), and the third isolate 4 (2^2^). Therefore, each digit can have a value from 0 (no susceptibility to any of the three isolates) up to 7 (=1 + 2 + 4) denoting susceptibility to each of the three isolates. The resulting number (reverse-octal) defines the resistance/susceptibility patterns of the *Hvs* plants and their phenotypic classification (Gilmour, 1973; Limpert and Müller, 1994; Dreiseitl and Dinoor, 2004). The HaGiS program was used for transcription of RTAs into octal notation (Hermann et al., 1999).

## Results

The set of 687 individually harvested plants derived from 146 *Hvs* accessions was inoculated with 32 *Bgh* isolates (Supplementary Table 1S). Within this set there were 211 phenotypes (i.e., different combinations of resistance and susceptibility to the *Bgh* isolates used). Eighty-seven phenotypes were recorded in only one plant with a phenotypic frequency of 1, while the eight most frequent phenotypes were found in 202 plants (**Table 3**). The most frequent phenotype (77777777773) appeared in 56 plants and was susceptible to all 32 *Bgh* isolates. In contrast, 46 plants showed the second most frequent phenotype (00000000000) and was resistant to all 32 pathogen isolates (**Table 4**).

**Table 3:** Frequency of resistance phenotypes found in 687 *Hordeum vulgare* subsp. *spontaneum* plant progenies inoculated with 32 *Blumeria graminis* f. sp. *hordei* isolates.

Phenotype frequency	Number of phenotypes	Number of plant progenies
>6	8	202
6	6	36
5	29	145
4	16	64
3	23	69
2	42	84
1	87	87
Total	211	687

**Table 4:** The eight most common resistance phenotypes found among 687 individually harvested *Hordeum vulgare* subsp. *spontaneum* plants inoculated with 32 *Blumeria graminis* f. sp. *hordei* isolates.

Resistance phenotype^∗^	Phenotype frequency
77777777773	57
00000000000	46
37777777773	29
00000000073	26
00000000063	14
34615110773	12
00000000020	11
74715110773	7
Sum	202

*^∗^Resistance phenotype is coded with reverse-octal notation (Limpert and Müller, 1994).*

Eleven phenotypes (000000000xx), comprising 127 plants (18.5% of 687 total plants tested), were resistant to all non-Israeli *Bgh* isolates including four of the eight most frequent (00000000000, 00000000073, 00000000063, and 00000000020) (**Table 4**). At least 50% of plants of 25 accessions (17.1% of the 146 tested accessions) were resistant to all non-Israeli isolates and there were 100 resistant plants among the 120 plants from these accessions (**Table 5**). The remaining 27 resistant plants were found in 22 other accessions.

**Table 5:** Origin of 25 *Hordeum vulgare* subsp. *spontaneum* accessions for which at least half of the plant progenies were resistant to all 27 non-Israeli *Blumeria graminis* f. sp. *hordei* isolates.

IG Number^∗^	Country of origin	No. of plant progenies tested	No. of resistant progenies
38615	Syria	5	4
38624	Jordan	5	5
38628	Jordan	5	3
38629	Jordan	5	5
38639	Syria	5	5
39415	Syria	5	4
39515	Syria	5	4
39943	Syria	4	2
39986	Jordan	5	3
39993	Jordan	5	5
39994	Jordan	4	4
39997	Jordan	5	5
39998	Jordan	5	5
39999	Jordan	5	3
40001	Jordan	5	3
40003	Jordan	5	5
40007	Jordan	5	5
40018	Jordan	5	5
40030	Jordan	5	4
40076	Jordan	5	3
40168	Lebanon	5	5
110771	Syria	5	5
115795	Jordan	5	4
126414	Turkmenistan	5	3
135762	Jordan	2	1
Total		120	100

*^∗^IG Number, accession identification number in the gene bank of the International Center for Agriculture Research in the Dry Areas (ICARDA).*

Sixty-four of the 146 accessions (43.8%) originated from collections in Syria and 50 (34.2%) from Jordan (**Table 1**). Of the 25 accessions for which at least 50% of plants were resistant to all non-Israeli isolates, six originated from Syria (9.4% of Syrian accessions) and 17 from Jordan (34.0% of Jordanian accessions) (**Table 5**).

A total of 128 accessions were represented by five tested plants. Plants with a single identical phenotype were found in 47 of these accessions (36.7%; **Table 6**), and all of the plants from 11 accessions were resistant to every non-Israeli *Bgh* isolates (**Table 5**). Only four accessions (38636, 40038, 40095, and 110823) were fully susceptible. In contrast, in two accessions (116131 and 117892), each plant exhibited a different phenotype. Plants from each of the remaining 79 accessions displayed from two to four phenotypes (**Table 6**).

**Table 6:** Number of resistance phenotypes found among 128 accessions of *Hordeum vulgare* subsp. *spontaneum* inoculated with 32 *Blumeria graminis* f. sp. *hordei* isolates; five plant progenies were tested from each accession.

Number of resistance phenotypes per accession	Number of accessions
1	47
2	44
3	25
4	10
5	2
Total	128

Testing the set of *Hvs* plants with 32 *Bgh* isolates yielded 21,984 RTs. RT2 was the most frequent in 7,769 (=35.3%) *Hvs*–*Bgh* interactions. In contrast, RT0 was the least frequent and was only found in 1,178 (5.4%) interactions (**Table 7**). This RT denotes the greatest resistance (immunity) and was most frequently observed on plants inoculated with non-European *Hvv* isolates (mean of 85 RT0 per isolate). Inoculation with these isolates resulted in the lowest determined frequency recorded (mean of 117) for RT4 (fully susceptible). Conversely, the lowest frequency for RT0 was found among plants inoculated with *Hvs* isolates from Israel (mean of 7). Inoculation with these isolates also had the highest frequency (mean of 139) for RT4. RT1 (complete resistance), was also most often seen on plants inoculated with non-European *Hvv* isolates (83) and least frequently observed on plants inoculated with isolates found on *Hvs* in Israel (12).

**Table 7:** Number of response types developed in 687 *Hordeum vulgare* subsp. *spontaneum* plant progenies after inoculation with 32 *Blumeria graminis* f. sp. *hordei* isolates.

Response type	Number
0	1,178
1	1,503
2	7,769
3	7,558
4	3,976
Total	21,984

## Discussion

In this study, 127 out of 687 *Hvs* plants were resistant to all 27 non-Israeli *Bgh* isolates. This contrasts with previous reports describing the response of *Hvv* lines to *Bgh*. For example, seven out of 159 local landraces of the Spanish core collection were resistant to seven *Bgh* isolates (Silvar et al., 2009). Furthermore, only one out of 147 Chinese varieties was resistant to all 32 isolates of *Bgh* (Dreiseitl and Yang, 2007), and no powdery mildew resistance to 28 isolates was found among 129 genotypes obtained from the Serbian barley gene bank (Šurlan-Momirović et al., 2016). Thus, *Hvs* is recognized as a good source of broadly acting resistance to *Bgh*.

In the present investigation, there was great diversity of *Hvs* resistance to *Bgh*; 211 phenotypes were recorded among 146 accessions. One hundred and twenty-eight accessions were represented by five tested plants. All plants within each of 47 accessions were homogeneous, had identical phenotypes and in the set of 47 accessions 38 different phenotypes were recorded.

In contrast, 81 heterogeneous accessions always exhibited more than one phenotype (mean of 2.63 phenotypes). Hence, *Hvs* diversity of resistance to *Bgh* has developed naturally during long-term co-evolution of these organisms (Fischbeck et al., 1976; Moseman et al., 1981; Dreiseitl and Dinoor, 2004). It is likely that the high resistance diversity of accessions housed in gene banks might also has arisen first, from the original collection of bulked samples that were more likely to be heterogeneous, second, as a result of the open flowering nature of *Hvs* (Brown et al., 1978) leading to frequent out-pollination in multiplication plots, and third, from mechanical admixtures during successive cycles of seed increase (Dreiseitl, 2007).

Once RTAs reach a certain complexity the data are collated. Mathematical codes are the most suitable parameter, especially octal notation and separation data into triplets (Gilmour, 1973; Limpert and Müller, 1994). Therefore, the use of this notation for classifying pathotypes (physiologic races) has been recommended (Limpert et al., 1994). Since host–parasite relationships relate to compatibility responses, it was proposed to use octal notation also for classifying host resistances (Dreiseitl and Dinoor, 2004).

The present study set was composed of accessions selected from 363 accessions that had been tested previously and were heterogeneous in their response to well-characterized *Bgh* isolates. Forty-seven accessions had homogeneous phenotypes for all five tested plants. In previous tests, standard seed samples were used and each accession was composed of about 30 plants. However, in this report testing of “only” five plant progenies did not establish that these 47 accessions were heterogeneous. Testing more plants would result in reduced numbers of homogeneous accessions caused by fewer impurities arising from cross-pollination or mechanical admixtures.

The region where *Hvs* naturally occurs is subject to annual *Bgh* epidemics. As a result of co-evolution, the local pathogen population has adapted to specific resistances (Dreiseitl et al., 2006) that originate in this area and which were, or still are, fully effective worldwide (Jahoor and Fischbeck, 1987; Dreiseitl and Bockelman, 2003). The five pathogen isolates originating from collections in Israel, therefore, provide a deeper understanding of the diversity of resistance in the evaluated accessions. Although the RTAs suggest that the resistances are not durable, their effectiveness in other regions should be high.

All the recorded RTAs were compared with RTAs of standard barley genotypes with known resistance genes in order to identify resistance genes in the *Hvs* accessions. Nevertheless, this was satisfactory only in those accessions with RTAs composed of just one or a few avirulent RTs. Twenty-nine plants had the 37777777773 phenotype, which is resistant to the Japanese isolate Race I (Hiura and Heta, 1955). This phenotype can be conditioned by the presence of the following two resistance genes – *Mla8*, which is already well characterized, produced RT0 in 13 plants, while a lesser known resistance, originally designated *Ml(Ch)* and present in many Chinese *Hvv* accessions (Dreiseitl and Yang, 2007), expressed RT2 in 16 plants. The chromosomal location of the latter gene is unknown and some accessions with *Mla8* might contain both resistances. In some plants, one or two of these genes may be present along with unidentified gene(s) that mask the corresponding responses to the pathogen. Their combined presence cannot, therefore, be established using conventional screening tests.

Immunity (RT0) to Race I was found in four plants of accession 38615 and six plants from five other accessions, although these genotypes also showed RT0 to two Israeli isolates. This set of three avirulences typifies the presence of the *Ml(Lo)* (Dreiseitl, 2011b). All the above 10 plants had a range of avirulent responses (mostly RT2) following inoculation with some of the other 29 isolates suggesting the presence of unidentified resistances in addition to *Ml(Lo)*.

The two most important basic requirements for genetic resistance of hosts to pathogens are effectiveness and durability. Effectiveness can be defined by the host response to individual pathotypes – e.g., the effectiveness of *Mla8* against the Japanese Race I is very high (RT0). However, it is more appropriate to characterize effectiveness at the population level. For example, *Mla8* is ineffective against the European *Bgh* population is zero because no isolate avirulent to *Mla8* was present in the population. The minimum requirement for durability is that resistance is maintained throughout a host’s lifespan.

In summary, genetic resistance to *Bgh* can be divided into qualitative resistance conditioned by major genes of specific resistance (Jørgensen, 1994), quantitative resistance conditioned by minor genes (Niks et al., 2015), and durable *mlo* resistance gene (Jørgensen, 1992). The latter has not yet been found in *Hvs*. Many specific resistance genes have been detected in *Hvs* (Dreiseitl, 2014) and many others can be hypothesized based on the present findings. However, using specific resistances to *Bgh* singly is not recommended for breeding barley varieties. Nevertheless, they can be used in varieties when combined with *mlo*. This is a useful strategy since the pathogen can gradually adapt even to partial virulence to this durable resistance gene (Schwarzbach, 1987) and erode its effectiveness. In such combinations, an effective specific resistance can prevent the erosion of *mlo* efficacy. A new method may also be devised that will improve the durability of specific resistances (Brown, 2015). Detecting specific resistances is, therefore, important first, for their potential use in breeding programs; second, for easier detection of minor genes that are frequently masked by specific resistance genes; third for increasing our knowledge of the occurrence and role of resistance in natural ecosystems.

## Author Contributions

The author confirms being the sole contributor of this work and approved it for publication.

## Conflict of Interest Statement

The author declares that the research was conducted in the absence of any commercial or financial relationships that could be construed as a potential conflict of interest.
