# Supplementary material for: Heterogeneity of Powdery Mildew Resistance Revealed in Accessions of the ICARDA Wild Barley Collection
Source: Front Plant Sci. 2017 Feb 14;8:202. doi: 10.3389/fpls.2017.00202 (PMC5307490; doi:10.3389/fpls.2017.00202)
Supplement: Supplementary file 1 [file Table_1.DOC]

Table 1S Resistance phenotypes of 687 *Hordeum vulgare* subsp. *spontaneum* plant progenies inoculated with 32 *Blumeria graminis* f. sp. *hordei* isolates

| Accession | Plant | *Blumeria graminis* f. sp. *hordei* isolate* | | | | | | | | | | | | | | | | | | | | | | | | | | | | | | | |
| --- | --- | --- | --- | --- | --- | --- | --- | --- | --- | --- | --- | --- | --- | --- | --- | --- | --- | --- | --- | --- | --- | --- | --- | --- | --- | --- | --- | --- | --- | --- | --- | --- | --- |
|  | progeny | 1 | 2 | 3 | 4 | 5 | 6 | 7 | 8 | 9 | 10 | 11 | 12 | 13 | 14 | 15 | 16 | 17 | 18 | 19 | 20 | 21 | 22 | 23 | 24 | 25 | 26 | 27 | 28 | 29 | 30 | 31 | 32 |
| 38612 | 1 | 4† | 1 | 2 | 2 | 1 | 2 | 4 | 2 | 2 | 4 | 2 | 4 | 2 | 4 | 4 | 1 | 2 | 1 | 2 | 2 | 2 | 2 | 2 | 2 | 2 | 2 | 4 | 3 | 4 | 4 | 4 | 4 |
| 38612 | 2 | 4 | 4 | 3 | 4 | 4 | 4 | 4 | 4 | 4 | 4 | 4 | 3 | 4 | 4 | 3 | 4 | 4 | 3 | 3 | 4 | 4 | 4 | 3 | 4 | 4 | 4 | 4 | 4 | 4 | 4 | 4 | 4 |
| 38612 | 3 | 3 | 4 | 3 | 4 | 4 | 4 | 4 | 4 | 4 | 4 | 4 | 3 | 4 | 4 | 3 | 4 | 4 | 3 | 3 | 4 | 4 | 4 | 4 | 4 | 4 | 4 | 4 | 3 | 4 | 4 | 4 | 4 |
| 38612 | 4 | 4 | 1 | 2 | 2 | 2 | 2 | 4 | 2 | 2 | 4 | 2 | 4 | 2 | 4 | 4 | 2 | 2 | 2 | 1 | 2 | 2 | 2 | 2 | 2 | 2 | 2 | 4 | 3 | 4 | 4 | 4 | 4 |
| 38612 | 5 | 4 | 4 | 3 | 4 | 4 | 4 | 4 | 4 | 4 | 4 | 4 | 4 | 4 | 4 | 3 | 4 | 4 | 3 | 3 | 4 | 4 | 4 | 4 | 4 | 4 | 4 | 4 | 3 | 4 | 4 | 4 | 4 |
| 38615 | 1 | 0 | 0 | 0 | 2 | 0 | 0 | 0 | 1 | 1 | 2 | 2 | 2 | 1 | 1 | 1 | 1 | 1 | 1 | 0 | 2 | 1 | 2 | 2 | 0 | 1 | 1 | 0 | 3 | 4 | 2 | 0 | 0 |
| 38615 | 2 | 0 | 0 | 0 | 0 | 0 | 1 | 0 | 2 | 2 | 2 | 2 | 0 | 0 | 0 | 2 | 1 | 0 | 1 | 0 | 2 | 1 | 2 | 0 | 1 | 2 | 1 | 0 | 4 | 3 | 2 | 0 | 0 |
| 38615 | 3 | 0 | 0 | 0 | 0 | 0 | 2 | 0 | 2 | 0 | 2 | 2 | 0 | 0 | 2 | 1 | 1 | 0 | 1 | 2 | 2 | 2 | 2 | 0 | 1 | 2 | 1 | 0 | 3 | 4 | 2 | 0 | 0 |
| 38615 | 4 | 4 | 2 | 2 | 2 | 3 | 4 | 2 | 3 | 3 | 3 | 4 | 3 | 4 | 3 | 2 | 3 | 3 | 3 | 3 | 3 | 3 | 4 | 4 | 4 | 4 | 4 | 3 | 3 | 4 | 3 | 4 | 3 |
| 38615 | 5 | 0 | 0 | 0 | 1 | 0 | 2 | 0 | 2 | 1 | 2 | 2 | 0 | 0 | 2 | 2 | 2 | 0 | 2 | 0 | 2 | 2 | 1 | 0 | 1 | 2 | 1 | 0 | 3 | 4 | 2 | 0 | 0 |
| 38622 | 1 | 2 | 3 | 0 | 2 | 3 | 3 | 3 | 3 | 2 | 3 | 3 | 3 | 2 | 3 | 4 | 4 | 4 | 3 | 2 | 4 | 2 | 3 | 3 | 3 | 4 | 4 | 3 | 4 | 3 | 3 | 3 | 4 |
| 38622 | 2 | 3 | 3 | 2 | 3 | 3 | 3 | 3 | 3 | 2 | 3 | 3 | 3 | 2 | 4 | 4 | 3 | 2 | 3 | 4 | 4 | 3 | 2 | 3 | 3 | 4 | 4 | 3 | 4 | 4 | 3 | 4 | 4 |
| 38622 | 3 | 2 | 3 | 0 | 4 | 2 | 3 | 3 | 4 | 4 | 4 | 3 | 4 | 4 | 4 | 4 | 4 | 2 | 3 | 2 | 4 | 4 | 4 | 4 | 4 | 4 | 4 | 2 | 4 | 4 | 3 | 4 | 4 |
| 38624 | 1 | 2 | 0 | 0 | 1 | 1 | 2 | 2 | 2 | 2 | 1 | 2 | 2 | 2 | 2 | 2 | 2 | 2 | 2 | 2 | 2 | 2 | 0 | 2 | 2 | 2 | 2 | 2 | 4 | 4 | 3 | 4 | 4 |
| 38624 | 2 | 0 | 2 | 0 | 0 | 0 | 2 | 0 | 2 | 2 | 0 | 2 | 2 | 2 | 2 | 2 | 2 | 0 | 2 | 0 | 2 | 2 | 0 | 2 | 2 | 2 | 2 | 2 | 3 | 4 | 4 | 4 | 4 |
| 38624 | 3 | 0 | 0 | 0 | 0 | 0 | 2 | 0 | 2 | 2 | 1 | 2 | 2 | 2 | 2 | 1 | 2 | 0 | 2 | 0 | 2 | 2 | 0 | 2 | 2 | 0 | 1 | 2 | 3 | 4 | 3 | 3 | 3 |
| 38624 | 4 | 1 | 0 | 2 | 2 | 2 | 1 | 0 | 2 | 2 | 2 | 2 | 1 | 0 | 1 | 1 | 1 | 2 | 2 | 0 | 1 | 1 | 0 | 1 | 1 | 0 | 0 | 0 | 3 | 4 | 3 | 3 | 3 |
| 38624 | 5 | 1 | 1 | 2 | 2 | 1 | 0 | 0 | 2 | 1 | 1 | 2 | 2 | 0 | 0 | 0 | 0 | 2 | 0 | 0 | 0 | 0 | 0 | 1 | 1 | 0 | 1 | 0 | 3 | 4 | 3 | 3 | 3 |
| 38628 | 1 | 0 | 0 | 0 | 0 | 0 | 1 | 0 | 0 | 1 | 0 | 2 | 2 | 1 | 2 | 2 | 0 | 0 | 0 | 0 | 2 | 0 | 0 | 1 | 1 | 0 | 1 | 2 | 4 | 4 | 4 | 4 | 4 |
| 38628 | 2 | 0 | 0 | 0 | 0 | 0 | 0 | 0 | 1 | 1 | 0 | 2 | 2 | 1 | 2 | 2 | 0 | 0 | 0 | 0 | 2 | 2 | 0 | 1 | 1 | 0 | 2 | 2 | 4 | 4 | 4 | 4 | 4 |
| 38628 | 3 | 3 | 2 | 2 | 2 | 2 | 2 | 4 | 2 | 2 | 4 | 2 | 4 | 2 | 3 | 4 | 2 | 2 | 2 | 2 | 2 | 2 | 2 | 2 | 2 | 3 | 2 | 3 | 3 | 4 | 4 | 4 | 3 |
| 38628 | 4 | 1 | 2 | 0 | 0 | 0 | 2 | 0 | 2 | 2 | 1 | 2 | 2 | 2 | 2 | 2 | 2 | 0 | 2 | 0 | 2 | 2 | 0 | 2 | 2 | 0 | 2 | 2 | 4 | 4 | 4 | 3 | 4 |
| 38628 | 5 | 3 | 1 | 0 | 2 | 0 | 2 | 3 | 2 | 2 | 3 | 2 | 3 | 2 | 3 | 3 | 2 | 2 | 2 | 2 | 2 | 2 | 2 | 2 | 2 | 3 | 2 | 3 | 4 | 4 | 4 | 3 | 3 |
| 38629 | 1 | 0 | 0 | 0 | 0 | 0 | 2 | 0 | 0 | 2 | 0 | 2 | 2 | 2 | 2 | 2 | 2 | 0 | 2 | 0 | 2 | 2 | 0 | 2 | 2 | 0 | 2 | 2 | 3 | 4 | 3 | 3 | 3 |
| 38629 | 2 | 0 | 0 | 0 | 1 | 0 | 2 | 0 | 2 | 2 | 0 | 2 | 2 | 2 | 2 | 2 | 2 | 1 | 2 | 0 | 2 | 2 | 0 | 2 | 2 | 0 | 2 | 2 | 4 | 4 | 4 | 4 | 4 |
| 38629 | 3 | 0 | 0 | 0 | 1 | 0 | 2 | 0 | 0 | 2 | 2 | 2 | 2 | 2 | 0 | 0 | 1 | 2 | 2 | 0 | 2 | 0 | 2 | 2 | 0 | 2 | 2 | 2 | 3 | 4 | 3 | 3 | 3 |
| 38629 | 4 | 0 | 0 | 0 | 0 | 0 | 2 | 0 | 2 | 2 | 0 | 2 | 2 | 2 | 2 | 2 | 2 | 2 | 2 | 0 | 2 | 2 | 0 | 2 | 2 | 0 | 2 | 2 | 4 | 4 | 3 | 4 | 3 |
| 38629 | 5 | 0 | 0 | 0 | 0 | 0 | 2 | 1 | 2 | 2 | 0 | 2 | 2 | 2 | 2 | 2 | 2 | 2 | 2 | 0 | 2 | 2 | 0 | 2 | 2 | 0 | 2 | 2 | 4 | 4 | 4 | 3 | 4 |
| 38636 | 1 | 4 | 4 | 4 | 4 | 4 | 4 | 4 | 4 | 4 | 4 | 4 | 4 | 4 | 4 | 4 | 4 | 4 | 4 | 4 | 4 | 4 | 4 | 4 | 4 | 4 | 4 | 4 | 3 | 4 | 3 | 3 | 3 |
| 38636 | 2 | 4 | 4 | 4 | 4 | 4 | 4 | 4 | 4 | 4 | 4 | 4 | 4 | 4 | 4 | 4 | 4 | 4 | 4 | 4 | 4 | 4 | 4 | 4 | 4 | 4 | 4 | 4 | 3 | 4 | 3 | 3 | 3 |
| 38636 | 3 | 4 | 4 | 4 | 4 | 4 | 4 | 4 | 4 | 4 | 4 | 4 | 4 | 4 | 4 | 4 | 4 | 4 | 4 | 4 | 4 | 4 | 4 | 4 | 4 | 4 | 4 | 4 | 3 | 4 | 3 | 3 | 3 |
| 38636 | 4 | 4 | 4 | 4 | 4 | 4 | 4 | 4 | 4 | 4 | 4 | 4 | 4 | 4 | 4 | 4 | 4 | 4 | 4 | 4 | 4 | 4 | 4 | 4 | 4 | 4 | 4 | 4 | 3 | 4 | 3 | 3 | 3 |
| 38636 | 5 | 4 | 4 | 4 | 4 | 4 | 4 | 4 | 4 | 4 | 4 | 4 | 4 | 4 | 4 | 4 | 4 | 4 | 4 | 4 | 4 | 4 | 4 | 4 | 4 | 4 | 4 | 4 | 3 | 4 | 3 | 3 | 3 |
| 38639 | 1 | 2 | 2 | 2 | 2 | 2 | 2 | 2 | 2 | 2 | 2 | 2 | 2 | 2 | 2 | 2 | 2 | 2 | 2 | 2 | 2 | 2 | 2 | 2 | 2 | 2 | 2 | 2 | 3 | 2 | 2 | 3 | 2 |
| 38639 | 2 | 2 | 2 | 2 | 2 | 2 | 2 | 2 | 2 | 2 | 2 | 2 | 2 | 2 | 2 | 2 | 2 | 2 | 2 | 2 | 2 | 2 | 2 | 2 | 2 | 2 | 2 | 2 | 3 | 2 | 2 | 3 | 2 |
| 38639 | 3 | 0 | 0 | 0 | 0 | 0 | 0 | 1 | 0 | 0 | 0 | 0 | 0 | 0 | 0 | 0 | 0 | 1 | 0 | 0 | 0 | 0 | 0 | 0 | 0 | 0 | 0 | 0 | 3 | 2 | 2 | 3 | 2 |
| 38639 | 4 | 0 | 0 | 0 | 0 | 0 | 0 | 1 | 0 | 0 | 0 | 0 | 0 | 0 | 0 | 0 | 0 | 1 | 0 | 0 | 0 | 0 | 0 | 0 | 0 | 0 | 0 | 0 | 3 | 2 | 2 | 3 | 2 |
| 38639 | 5 | 0 | 0 | 0 | 0 | 0 | 0 | 1 | 0 | 0 | 0 | 0 | 0 | 0 | 0 | 0 | 0 | 1 | 0 | 0 | 0 | 0 | 0 | 0 | 0 | 0 | 0 | 0 | 3 | 2 | 2 | 3 | 2 |
| 39409 | 1 | 2 | 4 | 0 | 1 | 4 | 4 | 0 | 3 | 3 | 4 | 4 | 3 | 3 | 2 | 4 | 4 | 3 | 3 | 4 | 4 | 4 | 3 | 3 | 3 | 3 | 3 | 2 | 3 | 3 | 3 | 3 | 2 |
| 39409 | 2 | 2 | 4 | 0 | 1 | 4 | 4 | 0 | 3 | 3 | 3 | 4 | 3 | 4 | 2 | 4 | 4 | 3 | 3 | 3 | 3 | 4 | 3 | 3 | 3 | 4 | 3 | 2 | 3 | 3 | 3 | 3 | 2 |
| 39409 | 3 | 2 | 4 | 0 | 1 | 4 | 4 | 2 | 3 | 3 | 4 | 3 | 3 | 4 | 2 | 3 | 4 | 4 | 3 | 4 | 3 | 3 | 3 | 3 | 3 | 4 | 3 | 2 | 3 | 3 | 3 | 3 | 2 |
| 39409 | 4 | 2 | 4 | 0 | 1 | 4 | 4 | 2 | 3 | 3 | 4 | 3 | 3 | 4 | 2 | 4 | 4 | 4 | 3 | 4 | 4 | 4 | 4 | 3 | 3 | 4 | 3 | 2 | 3 | 3 | 3 | 3 | 2 |
| 39409 | 5 | 2 | 4 | 0 | 1 | 4 | 4 | 2 | 4 | 3 | 4 | 4 | 4 | 3 | 2 | 4 | 4 | 4 | 4 | 4 | 4 | 4 | 4 | 4 | 4 | 4 | 3 | 2 | 3 | 3 | 3 | 3 | 2 |
| 39411 | 1 | 4 | 4 | 2 | 0 | 1 | 4 | 4 | 4 | 4 | 4 | 2 | 2 | 4 | 2 | 4 | 4 | 2 | 1 | 4 | 2 | 2 | 2 | 2 | 2 | 4 | 4 | 4 | 4 | 4 | 4 | 4 | 4 |
| 39411 | 2 | 4 | 4 | 2 | 1 | 1 | 4 | 4 | 4 | 4 | 4 | 2 | 2 | 4 | 2 | 4 | 4 | 2 | 1 | 4 | 2 | 2 | 2 | 2 | 2 | 4 | 4 | 4 | 4 | 4 | 4 | 4 | 4 |
| 39411 | 3 | 4 | 4 | 2 | 1 | 1 | 4 | 3 | 4 | 4 | 4 | 1 | 2 | 4 | 2 | 4 | 4 | 2 | 1 | 4 | 2 | 2 | 2 | 2 | 2 | 4 | 4 | 4 | 4 | 4 | 4 | 4 | 4 |
| 39411 | 4 | 4 | 4 | 2 | 1 | 1 | 4 | 4 | 4 | 4 | 4 | 2 | 2 | 4 | 2 | 4 | 4 | 2 | 1 | 4 | 2 | 2 | 2 | 2 | 1 | 4 | 4 | 4 | 4 | 4 | 4 | 4 | 4 |
| 39411 | 5 | 4 | 4 | 2 | 0 | 1 | 4 | 3 | 4 | 4 | 4 | 2 | 2 | 4 | 2 | 4 | 4 | 2 | 1 | 4 | 2 | 2 | 2 | 2 | 2 | 4 | 4 | 4 | 3 | 4 | 4 | 4 | 4 |
| 39412 | 1 | 2 | 2 | 0 | 2 | 2 | 2 | 2 | 2 | 2 | 2 | 2 | 3 | 2 | 2 | 2 | 3 | 3 | 2 | 2 | 2 | 2 | 2 | 2 | 2 | 3 | 2 | 2 | 3 | 3 | 4 | 4 | 4 |
| 39412 | 2 | 2 | 2 | 0 | 2 | 2 | 2 | 2 | 2 | 2 | 2 | 2 | 3 | 2 | 2 | 2 | 2 | 2 | 2 | 2 | 2 | 2 | 2 | 2 | 2 | 3 | 2 | 2 | 4 | 2 | 2 | 3 | 4 |
| 39412 | 3 | 2 | 2 | 0 | 2 | 2 | 2 | 2 | 2 | 2 | 2 | 2 | 3 | 2 | 2 | 2 | 2 | 2 | 2 | 2 | 2 | 2 | 2 | 2 | 2 | 3 | 2 | 2 | 3 | 2 | 2 | 3 | 4 |
| 39412 | 4 | 2 | 2 | 0 | 2 | 2 | 2 | 2 | 2 | 2 | 2 | 2 | 3 | 2 | 2 | 2 | 2 | 2 | 2 | 2 | 2 | 2 | 2 | 2 | 2 | 3 | 2 | 2 | 3 | 2 | 2 | 3 | 4 |
| 39412 | 5 | 2 | 2 | 0 | 2 | 2 | 2 | 2 | 2 | 2 | 2 | 2 | 3 | 2 | 2 | 2 | 2 | 2 | 2 | 2 | 2 | 2 | 2 | 2 | 2 | 3 | 2 | 2 | 3 | 2 | 2 | 3 | 3 |
| 39415 | 1 | 4 | 4 | 0 | 2 | 2 | 3 | 3 | 3 | 2 | 3 | 2 | 3 | 2 | 3 | 2 | 2 | 3 | 2 | 4 | 3 | 4 | 4 | 3 | 4 | 4 | 2 | 2 | 3 | 3 | 3 | 2 | 3 |
| 39415 | 2 | 2 | 2 | 2 | 2 | 2 | 2 | 2 | 2 | 2 | 2 | 2 | 2 | 2 | 2 | 2 | 2 | 2 | 2 | 2 | 2 | 2 | 2 | 2 | 2 | 2 | 2 | 2 | 3 | 2 | 2 | 2 | 2 |
| 39415 | 3 | 2 | 2 | 2 | 2 | 2 | 2 | 2 | 2 | 2 | 2 | 2 | 2 | 2 | 2 | 2 | 2 | 2 | 2 | 2 | 2 | 2 | 2 | 2 | 2 | 2 | 2 | 2 | 3 | 2 | 2 | 2 | 2 |
| 39415 | 4 | 2 | 2 | 2 | 2 | 2 | 2 | 2 | 2 | 2 | 2 | 2 | 2 | 2 | 2 | 2 | 2 | 2 | 2 | 2 | 2 | 2 | 2 | 2 | 2 | 2 | 2 | 2 | 3 | 2 | 2 | 2 | 2 |
| 39415 | 5 | 2 | 2 | 2 | 2 | 2 | 2 | 2 | 2 | 2 | 2 | 2 | 2 | 2 | 2 | 2 | 2 | 2 | 2 | 2 | 2 | 2 | 2 | 2 | 2 | 2 | 2 | 2 | 3 | 2 | 2 | 2 | 2 |
| 39421 | 1 | 3 | 4 | 0 | 3 | 3 | 2 | 3 | 2 | 2 | 4 | 2 | 2 | 3 | 2 | 3 | 2 | 4 | 2 | 4 | 3 | 3 | 4 | 3 | 2 | 4 | 2 | 2 | 4 | 4 | 4 | 4 | 4 |
| 39421 | 2 | 2 | 0 | 0 | 1 | 4 | 2 | 2 | 2 | 2 | 2 | 2 | 3 | 3 | 2 | 2 | 2 | 3 | 2 | 4 | 4 | 2 | 4 | 2 | 2 | 4 | 2 | 2 | 4 | 4 | 4 | 4 | 4 |
| 39421 | 3 | 2 | 0 | 0 | 0 | 2 | 2 | 1 | 2 | 2 | 2 | 2 | 2 | 2 | 2 | 2 | 2 | 0 | 2 | 2 | 2 | 2 | 2 | 2 | 2 | 2 | 2 | 2 | 4 | 4 | 4 | 4 | 4 |
| 39421 | 4 | 3 | 4 | 0 | 3 | 4 | 2 | 3 | 2 | 2 | 3 | 2 | 2 | 3 | 2 | 3 | 2 | 4 | 2 | 4 | 3 | 3 | 4 | 4 | 2 | 4 | 2 | 2 | 4 | 4 | 4 | 4 | 4 |
| 39421 | 5 | 3 | 4 | 0 | 3 | 4 | 2 | 3 | 2 | 2 | 3 | 2 | 2 | 3 | 2 | 3 | 2 | 4 | 2 | 4 | 3 | 3 | 4 | 4 | 2 | 4 | 2 | 2 | 4 | 4 | 4 | 4 | 4 |
| 39441 | 1 | 2 | 4 | 0 | 1 | 4 | 2 | 1 | 2 | 2 | 4 | 3 | 4 | 2 | 2 | 2 | 3 | 4 | 2 | 4 | 4 | 2 | 3 | 3 | 2 | 4 | 2 | 2 | 3 | 4 | 3 | 3 | 3 |
| 39441 | 2 | 2 | 4 | 0 | 1 | 4 | 2 | 1 | 2 | 2 | 4 | 3 | 3 | 2 | 2 | 2 | 3 | 4 | 2 | 4 | 3 | 2 | 4 | 3 | 2 | 4 | 2 | 2 | 4 | 4 | 3 | 3 | 4 |
| 39441 | 3 | 2 | 4 | 0 | 1 | 4 | 2 | 1 | 2 | 2 | 4 | 3 | 3 | 2 | 2 | 2 | 3 | 4 | 2 | 4 | 3 | 2 | 4 | 3 | 2 | 4 | 2 | 2 | 4 | 4 | 4 | 3 | 3 |
| 39441 | 4 | 2 | 4 | 0 | 1 | 4 | 2 | 2 | 2 | 2 | 4 | 3 | 3 | 2 | 2 | 2 | 3 | 4 | 2 | 4 | 3 | 2 | 4 | 3 | 2 | 4 | 2 | 2 | 4 | 3 | 4 | 3 | 3 |
| 39441 | 5 | 2 | 4 | 0 | 0 | 4 | 2 | 0 | 2 | 2 | 4 | 3 | 3 | 2 | 2 | 2 | 3 | 4 | 2 | 4 | 3 | 2 | 4 | 3 | 2 | 4 | 2 | 2 | 4 | 4 | 3 | 3 | 3 |
| 39443 | 2 | 4 | 4 | 0 | 4 | 4 | 4 | 3 | 3 | 4 | 4 | 4 | 4 | 2 | 4 | 3 | 4 | 3 | 3 | 4 | 4 | 4 | 3 | 3 | 4 | 4 | 4 | 4 | 4 | 2 | 3 | 3 | 4 |
| 39443 | 3 | 3 | 4 | 1 | 4 | 4 | 4 | 4 | 4 | 3 | 4 | 4 | 4 | 2 | 4 | 4 | 4 | 4 | 4 | 4 | 4 | 4 | 4 | 4 | 4 | 4 | 4 | 4 | 4 | 2 | 3 | 3 | 4 |
| 39443 | 4 | 4 | 4 | 2 | 4 | 4 | 4 | 3 | 4 | 3 | 4 | 4 | 4 | 2 | 4 | 4 | 4 | 4 | 4 | 4 | 4 | 4 | 4 | 4 | 4 | 4 | 4 | 4 | 4 | 2 | 3 | 3 | 4 |
| 39443 | 5 | 1 | 1 | 2 | 1 | 2 | 1 | 2 | 2 | 2 | 1 | 1 | 2 | 2 | 2 | 1 | 1 | 2 | 2 | 2 | 2 | 2 | 2 | 2 | 2 | 2 | 1 | 2 | 3 | 3 | 2 | 2 | 2 |
| 39450 | 1 | 4 | 4 | 0 | 4 | 4 | 4 | 4 | 4 | 3 | 4 | 4 | 4 | 4 | 4 | 4 | 4 | 4 | 4 | 4 | 4 | 4 | 3 | 4 | 4 | 4 | 4 | 4 | 3 | 4 | 3 | 4 | 4 |
| 39450 | 2 | 2 | 2 | 0 | 1 | 2 | 3 | 2 | 3 | 2 | 2 | 3 | 3 | 3 | 2 | 2 | 3 | 2 | 3 | 2 | 3 | 3 | 3 | 3 | 2 | 3 | 2 | 3 | 3 | 3 | 2 | 4 | 3 |
| 39450 | 3 | 4 | 3 | 0 | 2 | 3 | 3 | 2 | 3 | 2 | 2 | 2 | 3 | 2 | 2 | 3 | 3 | 2 | 2 | 3 | 3 | 3 | 3 | 3 | 4 | 4 | 2 | 2 | 3 | 3 | 2 | 4 | 3 |
| 39450 | 4 | 4 | 4 | 0 | 4 | 4 | 4 | 4 | 4 | 4 | 4 | 4 | 4 | 4 | 4 | 4 | 4 | 4 | 4 | 4 | 4 | 4 | 4 | 4 | 4 | 4 | 4 | 4 | 4 | 4 | 3 | 4 | 3 |
| 39450 | 5 | 4 | 3 | 0 | 2 | 3 | 3 | 2 | 3 | 2 | 2 | 2 | 3 | 2 | 2 | 3 | 3 | 2 | 2 | 3 | 3 | 3 | 3 | 3 | 4 | 4 | 2 | 2 | 3 | 3 | 2 | 3 | 3 |
| 39468 | 1 | 2 | 3 | 2 | 4 | 3 | 3 | 3 | 2 | 2 | 3 | 2 | 2 | 2 | 2 | 2 | 2 | 4 | 2 | 3 | 2 | 3 | 4 | 3 | 3 | 3 | 2 | 3 | 4 | 4 | 3 | 4 | 4 |
| 39468 | 2 | 2 | 2 | 2 | 2 | 3 | 2 | 2 | 2 | 2 | 2 | 2 | 3 | 2 | 2 | 2 | 2 | 2 | 2 | 2 | 2 | 2 | 3 | 3 | 2 | 2 | 2 | 2 | 2 | 4 | 3 | 4 | 4 |
| 39468 | 3 | 2 | 2 | 1 | 2 | 2 | 2 | 1 | 2 | 2 | 2 | 2 | 2 | 2 | 2 | 2 | 2 | 2 | 1 | 2 | 3 | 2 | 3 | 3 | 2 | 2 | 2 | 2 | 2 | 3 | 2 | 2 | 3 |
| 39468 | 4 | 2 | 2 | 2 | 2 | 2 | 2 | 1 | 2 | 2 | 2 | 2 | 2 | 2 | 2 | 2 | 2 | 2 | 1 | 2 | 3 | 2 | 3 | 3 | 2 | 2 | 2 | 2 | 2 | 3 | 2 | 2 | 3 |
| 39468 | 5 | 2 | 2 | 2 | 2 | 2 | 2 | 2 | 2 | 2 | 2 | 2 | 2 | 2 | 2 | 2 | 2 | 2 | 2 | 2 | 3 | 2 | 3 | 3 | 2 | 2 | 2 | 2 | 2 | 3 | 2 | 2 | 3 |
| 39477 | 2 | 4 | 4 | 2 | 4 | 4 | 4 | 4 | 4 | 4 | 4 | 4 | 4 | 4 | 4 | 4 | 4 | 4 | 4 | 4 | 4 | 4 | 4 | 4 | 4 | 4 | 4 | 4 | 4 | 4 | 4 | 4 | 4 |
| 39477 | 3 | 4 | 4 | 2 | 0 | 1 | 4 | 2 | 4 | 4 | 4 | 1 | 1 | 4 | 0 | 4 | 4 | 1 | 1 | 4 | 0 | 1 | 0 | 1 | 1 | 4 | 4 | 4 | 4 | 4 | 4 | 4 | 4 |
| 39477 | 4 | 4 | 4 | 0 | 4 | 4 | 4 | 3 | 4 | 4 | 4 | 4 | 4 | 4 | 4 | 4 | 4 | 4 | 4 | 4 | 4 | 4 | 4 | 4 | 4 | 4 | 4 | 4 | 4 | 4 | 4 | 4 | 4 |
| 39477 | 5 | 4 | 4 | 0 | 0 | 0 | 4 | 2 | 4 | 3 | 4 | 0 | 0 | 4 | 0 | 4 | 4 | 0 | 1 | 4 | 1 | 0 | 1 | 1 | 0 | 4 | 4 | 3 | 4 | 4 | 4 | 3 | 4 |
| 39478 | 1 | 4 | 4 | 4 | 4 | 4 | 4 | 3 | 4 | 4 | 4 | 4 | 4 | 4 | 4 | 4 | 4 | 4 | 4 | 4 | 4 | 4 | 4 | 4 | 4 | 4 | 4 | 4 | 3 | 4 | 3 | 3 | 3 |
| 39478 | 2 | 4 | 4 | 2 | 4 | 4 | 4 | 4 | 4 | 4 | 4 | 4 | 4 | 4 | 4 | 4 | 4 | 4 | 4 | 4 | 4 | 4 | 4 | 4 | 4 | 4 | 4 | 4 | 4 | 4 | 3 | 3 | 3 |
| 39478 | 3 | 4 | 4 | 2 | 1 | 4 | 4 | 4 | 4 | 4 | 4 | 4 | 4 | 4 | 4 | 4 | 4 | 3 | 4 | 4 | 4 | 4 | 4 | 4 | 4 | 4 | 4 | 4 | 3 | 4 | 3 | 3 | 3 |
| 39478 | 4 | 4 | 4 | 0 | 4 | 4 | 4 | 4 | 4 | 4 | 4 | 4 | 4 | 4 | 4 | 4 | 4 | 4 | 4 | 4 | 4 | 4 | 4 | 4 | 4 | 4 | 4 | 4 | 4 | 4 | 3 | 4 | 3 |
| 39478 | 5 | 4 | 4 | 2 | 4 | 4 | 4 | 4 | 4 | 4 | 4 | 4 | 4 | 4 | 4 | 4 | 4 | 4 | 4 | 4 | 4 | 4 | 4 | 4 | 4 | 4 | 4 | 4 | 4 | 4 | 3 | 4 | 4 |
| 39480 | 1 | 4 | 4 | 0 | 1 | 1 | 2 | 0 | 2 | 3 | 3 | 1 | 2 | 3 | 0 | 4 | 2 | 0 | 0 | 3 | 1 | 1 | 1 | 1 | 0 | 2 | 3 | 0 | 4 | 2 | 0 | 3 | 3 |
| 39480 | 2 | 2 | 4 | 2 | 0 | 0 | 4 | 2 | 4 | 4 | 4 | 1 | 1 | 4 | 0 | 4 | 2 | 1 | 0 | 4 | 0 | 0 | 0 | 1 | 0 | 4 | 4 | 4 | 4 | 4 | 4 | 4 | 4 |
| 39480 | 3 | 4 | 4 | 2 | 2 | 4 | 4 | 2 | 4 | 4 | 4 | 4 | 4 | 4 | 4 | 4 | 4 | 4 | 4 | 4 | 4 | 4 | 4 | 4 | 4 | 4 | 4 | 4 | 4 | 4 | 4 | 4 | 3 |
| 39480 | 4 | 4 | 4 | 2 | 1 | 4 | 4 | 2 | 4 | 4 | 4 | 4 | 4 | 4 | 4 | 4 | 4 | 4 | 4 | 4 | 4 | 4 | 4 | 4 | 4 | 4 | 4 | 4 | 4 | 4 | 4 | 4 | 3 |
| 39480 | 5 | 4 | 2 | 1 | 1 | 2 | 2 | 2 | 4 | 3 | 2 | 4 | 2 | 3 | 2 | 4 | 3 | 2 | 2 | 3 | 2 | 2 | 0 | 2 | 2 | 4 | 3 | 4 | 4 | 4 | 4 | 4 | 4 |
| 39482 | 1 | 4 | 4 | 3 | 2 | 4 | 4 | 4 | 4 | 4 | 4 | 4 | 4 | 4 | 4 | 4 | 4 | 4 | 4 | 4 | 4 | 4 | 4 | 4 | 4 | 4 | 4 | 4 | 3 | 4 | 4 | 3 | 3 |
| 39482 | 2 | 4 | 4 | 3 | 2 | 4 | 4 | 4 | 4 | 3 | 4 | 4 | 3 | 4 | 4 | 4 | 4 | 4 | 4 | 4 | 4 | 4 | 4 | 4 | 4 | 4 | 4 | 4 | 3 | 4 | 4 | 3 | 3 |
| 39482 | 3 | 4 | 4 | 2 | 2 | 4 | 3 | 0 | 4 | 4 | 4 | 4 | 4 | 4 | 4 | 4 | 4 | 4 | 4 | 4 | 4 | 4 | 4 | 4 | 4 | 4 | 4 | 4 | 4 | 4 | 4 | 3 | 4 |
| 39482 | 4 | 4 | 4 | 4 | 2 | 4 | 4 | 3 | 4 | 4 | 4 | 4 | 4 | 4 | 4 | 4 | 3 | 4 | 4 | 4 | 4 | 4 | 4 | 4 | 4 | 4 | 4 | 4 | 4 | 4 | 4 | 4 | 4 |
| 39482 | 5 | 4 | 4 | 4 | 2 | 4 | 4 | 3 | 4 | 4 | 4 | 4 | 4 | 4 | 4 | 4 | 4 | 4 | 4 | 4 | 4 | 4 | 4 | 4 | 4 | 4 | 4 | 4 | 4 | 4 | 4 | 4 | 4 |
| 39484 | 1 | 4 | 4 | 4 | 3 | 4 | 4 | 4 | 4 | 4 | 4 | 4 | 4 | 4 | 4 | 4 | 4 | 4 | 4 | 4 | 4 | 4 | 4 | 4 | 4 | 4 | 4 | 4 | 4 | 4 | 4 | 4 | 4 |
| 39484 | 2 | 4 | 4 | 4 | 4 | 4 | 4 | 4 | 4 | 4 | 4 | 4 | 4 | 4 | 4 | 4 | 4 | 4 | 4 | 4 | 4 | 4 | 4 | 4 | 4 | 4 | 4 | 4 | 3 | 4 | 4 | 3 | 4 |
| 39484 | 3 | 4 | 4 | 3 | 4 | 4 | 4 | 4 | 4 | 4 | 4 | 4 | 4 | 4 | 4 | 4 | 3 | 4 | 4 | 4 | 4 | 4 | 4 | 4 | 4 | 4 | 4 | 4 | 3 | 4 | 4 | 3 | 3 |
| 39484 | 4 | 4 | 4 | 3 | 3 | 4 | 4 | 4 | 4 | 4 | 4 | 4 | 4 | 4 | 4 | 4 | 4 | 4 | 4 | 4 | 4 | 4 | 4 | 4 | 4 | 4 | 4 | 4 | 3 | 4 | 4 | 3 | 4 |
| 39484 | 5 | 4 | 4 | 3 | 3 | 4 | 4 | 3 | 4 | 4 | 4 | 4 | 4 | 4 | 4 | 4 | 4 | 4 | 4 | 4 | 4 | 4 | 4 | 4 | 4 | 4 | 4 | 4 | 2 | 4 | 4 | 4 | 3 |
| 39488 | 1 | 4 | 4 | 3 | 0 | 2 | 4 | 4 | 4 | 4 | 4 | 1 | 2 | 4 | 1 | 4 | 3 | 1 | 2 | 4 | 0 | 2 | 2 | 2 | 2 | 4 | 4 | 4 | 4 | 4 | 4 | 4 | 4 |
| 39488 | 2 | 4 | 4 | 3 | 1 | 2 | 4 | 4 | 4 | 4 | 4 | 2 | 2 | 4 | 1 | 4 | 4 | 2 | 2 | 4 | 0 | 2 | 2 | 2 | 2 | 4 | 4 | 4 | 4 | 4 | 4 | 4 | 3 |
| 39488 | 3 | 4 | 3 | 2 | 0 | 2 | 4 | 4 | 4 | 4 | 4 | 1 | 1 | 4 | 1 | 4 | 3 | 1 | 0 | 4 | 0 | 1 | 2 | 2 | 2 | 4 | 4 | 4 | 4 | 4 | 4 | 4 | 4 |
| 39488 | 4 | 4 | 4 | 3 | 1 | 2 | 4 | 4 | 4 | 4 | 4 | 1 | 1 | 4 | 1 | 3 | 4 | 1 | 1 | 4 | 0 | 1 | 2 | 2 | 1 | 4 | 4 | 4 | 4 | 4 | 4 | 4 | 4 |
| 39488 | 5 | 4 | 4 | 3 | 0 | 2 | 4 | 4 | 4 | 4 | 4 | 2 | 2 | 4 | 1 | 4 | 3 | 0 | 2 | 4 | 1 | 0 | 2 | 2 | 1 | 4 | 4 | 4 | 4 | 4 | 4 | 4 | 4 |
| 39490 | 1 | 2 | 2 | 2 | 2 | 3 | 2 | 2 | 2 | 2 | 2 | 2 | 2 | 2 | 2 | 2 | 2 | 3 | 2 | 2 | 2 | 3 | 3 | 4 | 2 | 3 | 2 | 2 | 3 | 4 | 4 | 3 | 4 |
| 39490 | 2 | 2 | 2 | 2 | 2 | 3 | 2 | 2 | 2 | 2 | 2 | 2 | 2 | 2 | 2 | 2 | 2 | 3 | 2 | 2 | 2 | 3 | 3 | 4 | 2 | 3 | 2 | 2 | 3 | 4 | 4 | 4 | 4 |
| 39490 | 3 | 2 | 2 | 2 | 2 | 3 | 2 | 2 | 2 | 2 | 2 | 2 | 2 | 2 | 2 | 2 | 2 | 3 | 2 | 2 | 2 | 3 | 3 | 4 | 2 | 3 | 2 | 2 | 4 | 4 | 4 | 4 | 4 |
| 39490 | 4 | 2 | 2 | 3 | 3 | 3 | 2 | 2 | 4 | 3 | 3 | 2 | 3 | 2 | 2 | 2 | 2 | 4 | 2 | 3 | 2 | 3 | 3 | 4 | 2 | 3 | 2 | 2 | 4 | 4 | 4 | 4 | 4 |
| 39490 | 5 | 2 | 2 | 3 | 4 | 3 | 2 | 2 | 4 | 3 | 3 | 2 | 3 | 2 | 2 | 2 | 0 | 4 | 2 | 3 | 2 | 3 | 3 | 3 | 2 | 3 | 2 | 2 | 4 | 4 | 4 | 4 | 4 |
| 39491 | 1 | 2 | 2 | 2 | 2 | 2 | 2 | 2 | 2 | 2 | 2 | 2 | 2 | 2 | 2 | 3 | 3 | 2 | 2 | 2 | 2 | 2 | 2 | 2 | 2 | 3 | 2 | 2 | 4 | 4 | 3 | 3 | 4 |
| 39491 | 2 | 2 | 2 | 2 | 2 | 2 | 3 | 2 | 2 | 2 | 2 | 2 | 2 | 2 | 2 | 3 | 3 | 3 | 2 | 2 | 3 | 3 | 3 | 4 | 3 | 3 | 2 | 2 | 4 | 4 | 3 | 4 | 3 |
| 39491 | 3 | 2 | 2 | 2 | 2 | 2 | 3 | 2 | 2 | 2 | 2 | 2 | 2 | 2 | 2 | 3 | 3 | 3 | 2 | 2 | 4 | 3 | 3 | 4 | 3 | 4 | 2 | 2 | 4 | 4 | 4 | 4 | 3 |
| 39491 | 4 | 4 | 4 | 4 | 1 | 2 | 4 | 4 | 4 | 4 | 4 | 2 | 2 | 4 | 1 | 4 | 4 | 2 | 2 | 4 | 2 | 2 | 2 | 2 | 2 | 4 | 4 | 4 | 4 | 4 | 4 | 4 | 3 |
| 39491 | 5 | 4 | 4 | 3 | 1 | 2 | 4 | 3 | 4 | 4 | 4 | 2 | 2 | 4 | 1 | 3 | 4 | 2 | 1 | 4 | 0 | 1 | 2 | 2 | 1 | 4 | 4 | 4 | 4 | 4 | 4 | 4 | 4 |
| 39495 | 1 | 4 | 4 | 2 | 4 | 4 | 4 | 3 | 4 | 4 | 4 | 4 | 4 | 4 | 4 | 4 | 4 | 4 | 4 | 4 | 4 | 4 | 4 | 4 | 4 | 4 | 4 | 4 | 4 | 4 | 2 | 4 | 4 |
| 39495 | 2 | 4 | 4 | 2 | 4 | 4 | 4 | 4 | 4 | 4 | 4 | 4 | 4 | 4 | 3 | 4 | 4 | 4 | 4 | 4 | 4 | 4 | 4 | 4 | 4 | 4 | 4 | 4 | 4 | 4 | 2 | 4 | 4 |
| 39495 | 3 | 4 | 4 | 2 | 4 | 4 | 4 | 4 | 4 | 4 | 4 | 4 | 4 | 4 | 4 | 4 | 4 | 4 | 4 | 4 | 4 | 4 | 4 | 4 | 4 | 4 | 4 | 4 | 4 | 4 | 2 | 4 | 4 |
| 39495 | 4 | 4 | 4 | 2 | 3 | 4 | 4 | 4 | 4 | 4 | 4 | 4 | 4 | 4 | 4 | 4 | 4 | 4 | 3 | 4 | 4 | 4 | 4 | 4 | 4 | 4 | 4 | 4 | 4 | 4 | 2 | 4 | 4 |
| 39495 | 5 | 4 | 4 | 2 | 4 | 4 | 3 | 4 | 4 | 4 | 4 | 3 | 4 | 4 | 4 | 4 | 4 | 3 | 3 | 4 | 3 | 3 | 4 | 4 | 4 | 4 | 3 | 3 | 3 | 3 | 3 | 4 | 3 |
| 39497 | 1 | 4 | 3 | 2 | 1 | 1 | 4 | 1 | 4 | 3 | 4 | 2 | 2 | 3 | 2 | 4 | 3 | 2 | 2 | 4 | 2 | 2 | 2 | 2 | 2 | 4 | 4 | 4 | 4 | 4 | 4 | 4 | 4 |
| 39497 | 2 | 4 | 4 | 2 | 0 | 2 | 4 | 2 | 4 | 4 | 3 | 2 | 2 | 4 | 2 | 4 | 4 | 2 | 1 | 4 | 2 | 2 | 1 | 2 | 2 | 4 | 4 | 4 | 4 | 4 | 4 | 4 | 4 |
| 39497 | 3 | 4 | 3 | 2 | 0 | 1 | 4 | 0 | 4 | 4 | 3 | 1 | 1 | 4 | 2 | 4 | 4 | 2 | 0 | 4 | 1 | 2 | 2 | 2 | 1 | 4 | 3 | 4 | 4 | 4 | 4 | 4 | 4 |
| 39497 | 4 | 4 | 3 | 2 | 1 | 1 | 4 | 2 | 4 | 4 | 4 | 2 | 2 | 4 | 2 | 4 | 4 | 2 | 1 | 4 | 1 | 2 | 2 | 2 | 1 | 4 | 4 | 4 | 4 | 4 | 4 | 4 | 4 |
| 39497 | 5 | 4 | 4 | 2 | 1 | 2 | 4 | 2 | 4 | 4 | 4 | 2 | 2 | 4 | 2 | 4 | 4 | 2 | 1 | 4 | 2 | 2 | 2 | 2 | 1 | 4 | 4 | 4 | 4 | 4 | 4 | 4 | 4 |
| 39499 | 1 | 4 | 4 | 2 | 0 | 1 | 4 | 1 | 4 | 4 | 4 | 0 | 1 | 4 | 1 | 4 | 4 | 1 | 1 | 4 | 1 | 1 | 1 | 1 | 1 | 4 | 4 | 4 | 4 | 4 | 4 | 4 | 4 |
| 39499 | 2 | 4 | 4 | 2 | 1 | 0 | 3 | 1 | 4 | 4 | 4 | 0 | 1 | 4 | 1 | 4 | 4 | 1 | 0 | 4 | 1 | 1 | 1 | 1 | 0 | 4 | 4 | 4 | 4 | 4 | 4 | 4 | 4 |
| 39499 | 3 | 4 | 4 | 2 | 0 | 2 | 4 | 2 | 4 | 3 | 4 | 2 | 2 | 4 | 1 | 4 | 4 | 2 | 2 | 4 | 2 | 2 | 1 | 2 | 1 | 4 | 3 | 4 | 4 | 4 | 4 | 4 | 4 |
| 39499 | 4 | 3 | 4 | 2 | 0 | 2 | 4 | 2 | 4 | 3 | 3 | 0 | 2 | 3 | 1 | 4 | 3 | 1 | 1 | 3 | 0 | 0 | 1 | 2 | 2 | 4 | 3 | 3 | 4 | 4 | 4 | 4 | 4 |
| 39499 | 5 | 4 | 4 | 2 | 0 | 0 | 3 | 1 | 4 | 4 | 4 | 1 | 2 | 4 | 1 | 4 | 4 | 2 | 0 | 4 | 0 | 1 | 2 | 1 | 2 | 4 | 4 | 4 | 4 | 4 | 4 | 4 | 4 |
| 39500 | 1 | 2 | 2 | 2 | 4 | 2 | 2 | 2 | 2 | 2 | 2 | 2 | 2 | 2 | 2 | 2 | 2 | 2 | 1 | 2 | 2 | 2 | 3 | 4 | 2 | 2 | 2 | 2 | 4 | 4 | 4 | 4 | 4 |
| 39500 | 2 | 2 | 2 | 2 | 2 | 2 | 2 | 2 | 2 | 2 | 2 | 2 | 2 | 2 | 2 | 2 | 2 | 2 | 2 | 2 | 3 | 2 | 3 | 4 | 2 | 2 | 2 | 2 | 4 | 4 | 4 | 4 | 3 |
| 39500 | 3 | 2 | 2 | 2 | 2 | 2 | 2 | 2 | 2 | 2 | 2 | 2 | 2 | 2 | 2 | 2 | 2 | 2 | 1 | 2 | 3 | 2 | 3 | 4 | 2 | 2 | 2 | 2 | 4 | 4 | 4 | 3 | 3 |
| 39500 | 4 | 2 | 2 | 2 | 4 | 2 | 2 | 2 | 2 | 2 | 2 | 2 | 2 | 2 | 2 | 2 | 2 | 2 | 2 | 2 | 2 | 2 | 3 | 3 | 2 | 2 | 2 | 2 | 4 | 4 | 4 | 3 | 3 |
| 39500 | 5 | 2 | 2 | 2 | 2 | 2 | 2 | 2 | 2 | 2 | 2 | 2 | 2 | 2 | 2 | 2 | 2 | 2 | 2 | 2 | 3 | 2 | 3 | 3 | 2 | 2 | 2 | 2 | 3 | 4 | 4 | 3 | 3 |
| 39503 | 1 | 2 | 2 | 2 | 3 | 2 | 2 | 2 | 2 | 2 | 3 | 2 | 2 | 2 | 2 | 2 | 2 | 3 | 2 | 2 | 2 | 2 | 3 | 3 | 2 | 2 | 2 | 2 | 4 | 4 | 4 | 4 | 4 |
| 39503 | 2 | 2 | 2 | 2 | 3 | 2 | 2 | 2 | 2 | 2 | 3 | 2 | 2 | 2 | 2 | 2 | 2 | 3 | 2 | 2 | 2 | 2 | 3 | 3 | 2 | 2 | 2 | 2 | 4 | 4 | 4 | 4 | 3 |
| 39503 | 3 | 2 | 2 | 2 | 3 | 2 | 2 | 2 | 2 | 2 | 3 | 2 | 2 | 2 | 2 | 2 | 2 | 3 | 2 | 2 | 2 | 2 | 3 | 3 | 2 | 2 | 2 | 2 | 3 | 4 | 4 | 4 | 3 |
| 39503 | 4 | 2 | 2 | 2 | 3 | 2 | 2 | 2 | 2 | 2 | 3 | 2 | 2 | 2 | 2 | 2 | 2 | 3 | 2 | 2 | 2 | 2 | 3 | 3 | 2 | 2 | 2 | 2 | 3 | 4 | 4 | 4 | 4 |
| 39503 | 5 | 2 | 2 | 2 | 3 | 2 | 2 | 2 | 2 | 2 | 3 | 2 | 2 | 2 | 2 | 2 | 2 | 3 | 2 | 2 | 2 | 2 | 3 | 3 | 2 | 2 | 2 | 2 | 3 | 4 | 4 | 4 | 4 |
| 39504 | 1 | 2 | 2 | 2 | 3 | 3 | 2 | 2 | 2 | 2 | 3 | 2 | 3 | 2 | 3 | 2 | 2 | 3 | 2 | 2 | 3 | 2 | 3 | 3 | 2 | 2 | 2 | 2 | 4 | 4 | 4 | 4 | 4 |
| 39504 | 2 | 2 | 2 | 2 | 3 | 3 | 2 | 2 | 2 | 2 | 3 | 2 | 3 | 2 | 3 | 2 | 2 | 3 | 2 | 2 | 3 | 2 | 3 | 3 | 2 | 2 | 2 | 2 | 4 | 4 | 4 | 4 | 4 |
| 39504 | 3 | 2 | 2 | 2 | 3 | 3 | 2 | 2 | 2 | 2 | 3 | 2 | 3 | 2 | 3 | 2 | 2 | 3 | 2 | 2 | 3 | 2 | 3 | 3 | 2 | 2 | 2 | 2 | 4 | 4 | 4 | 4 | 4 |
| 39504 | 4 | 2 | 2 | 2 | 3 | 3 | 2 | 2 | 2 | 2 | 3 | 2 | 3 | 2 | 3 | 2 | 2 | 3 | 2 | 2 | 3 | 2 | 3 | 3 | 2 | 2 | 2 | 2 | 4 | 4 | 4 | 4 | 4 |
| 39504 | 5 | 2 | 2 | 2 | 3 | 3 | 2 | 2 | 2 | 2 | 3 | 2 | 3 | 2 | 3 | 2 | 2 | 3 | 2 | 1 | 3 | 2 | 3 | 3 | 2 | 2 | 2 | 2 | 4 | 4 | 4 | 4 | 4 |
| 39505 | 1 | 2 | 2 | 2 | 2 | 2 | 2 | 2 | 2 | 2 | 2 | 2 | 2 | 2 | 2 | 2 | 2 | 2 | 1 | 2 | 2 | 2 | 3 | 3 | 2 | 2 | 2 | 2 | 4 | 3 | 3 | 3 | 4 |
| 39505 | 2 | 2 | 2 | 2 | 2 | 2 | 2 | 2 | 2 | 2 | 2 | 2 | 2 | 2 | 2 | 2 | 2 | 2 | 1 | 2 | 2 | 2 | 3 | 3 | 2 | 2 | 2 | 2 | 4 | 4 | 3 | 3 | 4 |
| 39505 | 3 | 2 | 2 | 2 | 2 | 2 | 2 | 2 | 2 | 2 | 2 | 2 | 1 | 2 | 2 | 2 | 2 | 2 | 0 | 2 | 2 | 2 | 3 | 3 | 2 | 2 | 2 | 2 | 4 | 3 | 3 | 3 | 4 |
| 39505 | 4 | 2 | 2 | 2 | 2 | 2 | 2 | 2 | 2 | 2 | 2 | 2 | 0 | 2 | 2 | 2 | 2 | 2 | 1 | 2 | 2 | 2 | 3 | 3 | 2 | 2 | 2 | 2 | 4 | 3 | 3 | 3 | 4 |
| 39505 | 5 | 2 | 2 | 2 | 2 | 2 | 2 | 2 | 2 | 2 | 2 | 2 | 1 | 2 | 2 | 2 | 2 | 2 | 1 | 2 | 2 | 2 | 3 | 3 | 2 | 2 | 2 | 2 | 4 | 3 | 3 | 4 | 4 |
| 39510 | 1 | 2 | 2 | 2 | 2 | 3 | 1 | 3 | 2 | 2 | 2 | 1 | 0 | 2 | 2 | 2 | 2 | 2 | 2 | 2 | 2 | 1 | 3 | 2 | 0 | 1 | 1 | 0 | 4 | 3 | 3 | 4 | 4 |
| 39510 | 2 | 2 | 4 | 2 | 4 | 3 | 2 | 4 | 3 | 3 | 3 | 3 | 2 | 2 | 3 | 2 | 2 | 2 | 2 | 4 | 3 | 2 | 4 | 2 | 3 | 4 | 2 | 2 | 4 | 3 | 3 | 4 | 4 |
| 39510 | 3 | 2 | 2 | 2 | 2 | 4 | 2 | 3 | 2 | 2 | 2 | 2 | 2 | 2 | 2 | 2 | 2 | 2 | 1 | 2 | 1 | 2 | 3 | 2 | 2 | 2 | 2 | 2 | 4 | 3 | 3 | 3 | 3 |
| 39510 | 4 | 2 | 1 | 2 | 2 | 3 | 2 | 3 | 2 | 2 | 2 | 2 | 2 | 2 | 2 | 2 | 2 | 2 | 2 | 2 | 2 | 2 | 3 | 2 | 2 | 2 | 2 | 2 | 3 | 3 | 3 | 3 | 3 |
| 39510 | 5 | 2 | 2 | 2 | 2 | 3 | 2 | 3 | 2 | 2 | 2 | 2 | 2 | 2 | 2 | 2 | 2 | 2 | 2 | 2 | 1 | 0 | 3 | 2 | 2 | 2 | 2 | 2 | 4 | 3 | 3 | 4 | 4 |
| 39513 | 1 | 3 | 3 | 2 | 4 | 3 | 3 | 4 | 4 | 3 | 3 | 4 | 3 | 3 | 4 | 4 | 4 | 4 | 2 | 3 | 4 | 4 | 4 | 4 | 3 | 3 | 3 | 3 | 4 | 0 | 3 | 3 | 4 |
| 39513 | 2 | 3 | 3 | 2 | 4 | 4 | 3 | 4 | 4 | 3 | 3 | 4 | 3 | 3 | 4 | 4 | 4 | 3 | 2 | 3 | 4 | 4 | 3 | 4 | 4 | 4 | 3 | 3 | 4 | 0 | 4 | 3 | 4 |
| 39513 | 3 | 3 | 3 | 2 | 3 | 4 | 3 | 3 | 3 | 3 | 3 | 3 | 3 | 3 | 4 | 4 | 3 | 4 | 2 | 3 | 4 | 3 | 3 | 3 | 3 | 4 | 4 | 3 | 4 | 2 | 3 | 3 | 4 |
| 39513 | 4 | 2 | 3 | 2 | 3 | 3 | 4 | 4 | 3 | 3 | 3 | 3 | 3 | 2 | 4 | 3 | 3 | 3 | 2 | 4 | 3 | 3 | 3 | 1 | 3 | 4 | 4 | 3 | 3 | 1 | 3 | 3 | 4 |
| 39513 | 5 | 2 | 3 | 2 | 3 | 3 | 3 | 3 | 3 | 3 | 3 | 3 | 3 | 2 | 3 | 4 | 3 | 3 | 2 | 3 | 3 | 3 | 3 | 2 | 3 | 4 | 3 | 3 | 3 | 0 | 3 | 3 | 3 |
| 39515 | 1 | 0 | 0 | 0 | 0 | 0 | 0 | 0 | 0 | 2 | 0 | 2 | 0 | 0 | 0 | 2 | 1 | 0 | 0 | 0 | 2 | 2 | 0 | 0 | 1 | 2 | 2 | 2 | 3 | 3 | 3 | 1 | 2 |
| 39515 | 2 | 0 | 0 | 0 | 0 | 0 | 0 | 0 | 0 | 2 | 0 | 2 | 0 | 0 | 0 | 2 | 2 | 0 | 0 | 1 | 2 | 2 | 0 | 0 | 2 | 2 | 2 | 2 | 4 | 3 | 3 | 2 | 1 |
| 39515 | 3 | 2 | 2 | 2 | 2 | 2 | 2 | 2 | 2 | 2 | 4 | 2 | 2 | 2 | 3 | 2 | 2 | 2 | 2 | 2 | 2 | 2 | 2 | 3 | 2 | 2 | 2 | 2 | 3 | 3 | 3 | 2 | 3 |
| 39515 | 4 | 0 | 0 | 0 | 0 | 0 | 0 | 0 | 0 | 2 | 0 | 2 | 0 | 0 | 1 | 2 | 1 | 1 | 0 | 2 | 2 | 2 | 0 | 2 | 2 | 2 | 2 | 2 | 3 | 3 | 3 | 2 | 2 |
| 39515 | 5 | 0 | 0 | 0 | 0 | 0 | 0 | 0 | 0 | 2 | 1 | 2 | 2 | 0 | 0 | 2 | 1 | 2 | 2 | 0 | 2 | 2 | 0 | 0 | 2 | 2 | 2 | 2 | 3 | 4 | 4 | 1 | 0 |
| 39516 | 1 | 0 | 2 | 0 | 2 | 1 | 2 | 1 | 2 | 2 | 2 | 2 | 2 | 2 | 2 | 1 | 2 | 0 | 2 | 2 | 2 | 2 | 2 | 2 | 2 | 0 | 2 | 2 | 3 | 3 | 3 | 0 | 0 |
| 39516 | 2 | 2 | 2 | 0 | 4 | 2 | 3 | 3 | 2 | 2 | 3 | 2 | 2 | 2 | 2 | 2 | 2 | 2 | 2 | 2 | 2 | 3 | 3 | 2 | 2 | 2 | 2 | 3 | 3 | 3 | 2 | 3 | 3 |
| 39516 | 3 | 2 | 2 | 0 | 1 | 2 | 2 | 2 | 2 | 2 | 2 | 2 | 2 | 2 | 2 | 2 | 2 | 2 | 2 | 2 | 2 | 2 | 2 | 2 | 2 | 2 | 2 | 3 | 3 | 2 | 2 | 3 | 2 |
| 39516 | 4 | 1 | 0 | 1 | 0 | 0 | 0 | 0 | 1 | 2 | 2 | 2 | 1 | 1 | 0 | 2 | 2 | 0 | 0 | 0 | 2 | 1 | 0 | 0 | 1 | 1 | 2 | 0 | 4 | 3 | 3 | 0 | 0 |
| 39516 | 5 | 2 | 2 | 1 | 3 | 2 | 3 | 3 | 2 | 2 | 3 | 2 | 2 | 2 | 2 | 2 | 2 | 2 | 2 | 2 | 2 | 3 | 3 | 2 | 2 | 2 | 2 | 3 | 3 | 3 | 2 | 3 | 3 |
| 39530 | 1 | 2 | 3 | 0 | 4 | 2 | 4 | 2 | 3 | 4 | 4 | 3 | 2 | 3 | 4 | 3 | 4 | 2 | 2 | 3 | 4 | 4 | 4 | 3 | 4 | 4 | 4 | 3 | 4 | 3 | 3 | 4 | 4 |
| 39530 | 2 | 2 | 3 | 0 | 4 | 2 | 3 | 2 | 3 | 3 | 3 | 3 | 2 | 3 | 3 | 3 | 4 | 1 | 1 | 3 | 4 | 3 | 3 | 3 | 3 | 4 | 3 | 3 | 3 | 3 | 3 | 3 | 4 |
| 39530 | 3 | 2 | 3 | 2 | 4 | 2 | 3 | 2 | 3 | 4 | 3 | 4 | 2 | 3 | 4 | 4 | 4 | 2 | 2 | 3 | 4 | 3 | 3 | 3 | 3 | 3 | 3 | 4 | 3 | 3 | 3 | 3 | 4 |
| 39530 | 4 | 2 | 3 | 2 | 4 | 2 | 4 | 2 | 3 | 3 | 4 | 4 | 2 | 3 | 4 | 3 | 4 | 2 | 2 | 3 | 4 | 4 | 4 | 3 | 3 | 4 | 4 | 3 | 4 | 3 | 4 | 2 | 4 |
| 39530 | 5 | 2 | 3 | 0 | 4 | 2 | 4 | 2 | 4 | 4 | 4 | 4 | 2 | 3 | 4 | 3 | 4 | 2 | 2 | 3 | 4 | 4 | 4 | 4 | 3 | 4 | 4 | 3 | 4 | 3 | 3 | 4 | 4 |
| 39541 | 1 | 3 | 2 | 3 | 2 | 2 | 4 | 3 | 2 | 3 | 2 | 2 | 3 | 4 | 3 | 3 | 3 | 4 | 4 | 3 | 4 | 4 | 3 | 3 | 4 | 4 | 3 | 2 | 2 | 3 | 2 | 3 | 3 |
| 39541 | 2 | 3 | 2 | 3 | 2 | 2 | 4 | 3 | 2 | 3 | 2 | 2 | 4 | 4 | 4 | 4 | 4 | 4 | 4 | 3 | 4 | 4 | 4 | 3 | 4 | 4 | 4 | 2 | 2 | 3 | 2 | 3 | 3 |
| 39541 | 3 | 3 | 2 | 3 | 2 | 2 | 4 | 3 | 2 | 3 | 2 | 2 | 3 | 4 | 3 | 4 | 4 | 4 | 4 | 3 | 4 | 4 | 4 | 4 | 4 | 4 | 4 | 2 | 2 | 3 | 3 | 3 | 3 |
| 39541 | 4 | 3 | 2 | 4 | 2 | 0 | 4 | 3 | 2 | 3 | 2 | 2 | 4 | 4 | 3 | 3 | 3 | 4 | 4 | 3 | 4 | 3 | 4 | 4 | 4 | 4 | 3 | 2 | 2 | 3 | 3 | 3 | 3 |
| 39541 | 5 | 3 | 2 | 3 | 2 | 2 | 4 | 3 | 2 | 3 | 2 | 2 | 4 | 4 | 3 | 4 | 4 | 4 | 4 | 3 | 4 | 3 | 4 | 4 | 4 | 4 | 3 | 2 | 2 | 3 | 3 | 3 | 3 |
| 39842 | 1 | 4 | 4 | 4 | 4 | 4 | 4 | 4 | 4 | 4 | 4 | 4 | 4 | 4 | 4 | 4 | 4 | 4 | 3 | 4 | 4 | 4 | 4 | 3 | 4 | 4 | 4 | 4 | 2 | 3 | 3 | 3 | 3 |
| 39842 | 2 | 4 | 4 | 4 | 4 | 3 | 4 | 4 | 4 | 3 | 4 | 3 | 4 | 3 | 4 | 4 | 4 | 4 | 3 | 4 | 4 | 4 | 4 | 4 | 4 | 4 | 4 | 4 | 2 | 3 | 3 | 3 | 3 |
| 39842 | 3 | 3 | 4 | 4 | 4 | 3 | 4 | 4 | 4 | 4 | 4 | 4 | 4 | 3 | 4 | 4 | 4 | 4 | 3 | 4 | 4 | 4 | 4 | 4 | 4 | 4 | 3 | 4 | 2 | 3 | 3 | 3 | 3 |
| 39842 | 4 | 3 | 4 | 4 | 4 | 4 | 4 | 4 | 4 | 4 | 4 | 4 | 4 | 4 | 3 | 4 | 4 | 4 | 3 | 4 | 4 | 4 | 4 | 4 | 4 | 4 | 3 | 4 | 2 | 3 | 3 | 3 | 3 |
| 39842 | 5 | 3 | 4 | 4 | 4 | 4 | 4 | 4 | 3 | 4 | 4 | 3 | 3 | 3 | 3 | 3 | 4 | 4 | 3 | 4 | 4 | 4 | 4 | 3 | 4 | 4 | 3 | 4 | 2 | 3 | 3 | 3 | 3 |
| 39844 | 1 | 3 | 1 | 1 | 3 | 4 | 2 | 2 | 2 | 4 | 3 | 3 | 4 | 2 | 3 | 2 | 2 | 3 | 3 | 2 | 3 | 3 | 4 | 3 | 4 | 2 | 2 | 2 | 4 | 3 | 4 | 4 | 4 |
| 39844 | 2 | 3 | 4 | 4 | 4 | 4 | 4 | 4 | 3 | 4 | 4 | 4 | 4 | 4 | 4 | 4 | 4 | 4 | 4 | 4 | 4 | 4 | 4 | 4 | 4 | 4 | 4 | 4 | 3 | 3 | 3 | 3 | 4 |
| 39844 | 3 | 4 | 4 | 4 | 4 | 3 | 4 | 4 | 4 | 4 | 4 | 4 | 4 | 4 | 4 | 3 | 4 | 3 | 3 | 3 | 3 | 3 | 4 | 4 | 3 | 4 | 3 | 3 | 3 | 3 | 3 | 3 | 3 |
| 39844 | 4 | 4 | 4 | 1 | 4 | 4 | 4 | 4 | 4 | 3 | 2 | 4 | 4 | 3 | 3 | 4 | 4 | 4 | 3 | 4 | 4 | 4 | 4 | 4 | 4 | 4 | 3 | 3 | 2 | 3 | 3 | 3 | 3 |
| 39844 | 5 | 4 | 4 | 1 | 4 | 4 | 4 | 4 | 4 | 3 | 2 | 4 | 4 | 3 | 4 | 4 | 4 | 4 | 4 | 4 | 4 | 4 | 4 | 4 | 4 | 4 | 3 | 3 | 2 | 3 | 3 | 3 | 3 |
| 39845 | 1 | 2 | 3 | 2 | 3 | 2 | 3 | 2 | 2 | 3 | 2 | 3 | 3 | 3 | 3 | 3 | 3 | 2 | 3 | 3 | 2 | 3 | 3 | 3 | 3 | 3 | 3 | 2 | 2 | 3 | 3 | 2 | 3 |
| 39845 | 2 | 2 | 3 | 0 | 4 | 2 | 4 | 2 | 2 | 3 | 2 | 4 | 4 | 3 | 4 | 3 | 4 | 4 | 3 | 3 | 4 | 4 | 3 | 3 | 3 | 4 | 3 | 4 | 2 | 3 | 3 | 2 | 3 |
| 39845 | 3 | 2 | 2 | 2 | 2 | 2 | 2 | 2 | 2 | 2 | 2 | 2 | 2 | 2 | 2 | 2 | 2 | 2 | 2 | 2 | 2 | 2 | 2 | 2 | 2 | 2 | 2 | 2 | 2 | 3 | 3 | 4 | 4 |
| 39845 | 4 | 4 | 4 | 0 | 4 | 4 | 4 | 4 | 4 | 4 | 4 | 4 | 4 | 4 | 4 | 4 | 4 | 4 | 4 | 4 | 4 | 4 | 4 | 3 | 4 | 4 | 4 | 4 | 3 | 3 | 3 | 4 | 3 |
| 39845 | 5 | 2 | 1 | 2 | 2 | 2 | 2 | 2 | 2 | 1 | 2 | 2 | 2 | 2 | 2 | 2 | 2 | 2 | 2 | 2 | 2 | 2 | 2 | 2 | 2 | 2 | 2 | 2 | 2 | 3 | 3 | 4 | 3 |
| 39851 | 3 | 2 | 2 | 2 | 3 | 2 | 0 | 2 | 2 | 2 | 3 | 2 | 2 | 2 | 2 | 2 | 2 | 2 | 2 | 3 | 2 | 2 | 2 | 2 | 0 | 1 | 2 | 0 | 3 | 3 | 3 | 3 | 3 |
| 39855 | 2 | 2 | 2 | 2 | 3 | 2 | 2 | 2 | 2 | 2 | 3 | 2 | 2 | 2 | 2 | 2 | 3 | 2 | 3 | 2 | 3 | 2 | 3 | 3 | 3 | 4 | 3 | 3 | 3 | 3 | 4 | 4 | 4 |
| 39855 | 3 | 2 | 2 | 2 | 3 | 2 | 2 | 2 | 2 | 2 | 3 | 2 | 2 | 2 | 2 | 2 | 3 | 2 | 3 | 2 | 3 | 2 | 3 | 3 | 3 | 4 | 3 | 3 | 3 | 3 | 4 | 4 | 4 |
| 39856 | 1 | 2 | 2 | 3 | 3 | 2 | 2 | 3 | 2 | 2 | 2 | 2 | 3 | 2 | 2 | 3 | 3 | 2 | 2 | 2 | 2 | 2 | 2 | 2 | 2 | 3 | 2 | 2 | 3 | 3 | 2 | 2 | 3 |
| 39856 | 3 | 2 | 2 | 3 | 3 | 2 | 2 | 3 | 2 | 2 | 2 | 2 | 3 | 2 | 2 | 3 | 3 | 2 | 2 | 2 | 2 | 2 | 2 | 2 | 2 | 3 | 2 | 2 | 3 | 3 | 2 | 2 | 3 |
| 39881 | 1 | 2 | 1 | 0 | 3 | 3 | 1 | 1 | 1 | 2 | 4 | 2 | 3 | 2 | 4 | 1 | 2 | 2 | 3 | 1 | 4 | 3 | 4 | 3 | 4 | 1 | 1 | 1 | 2 | 3 | 3 | 3 | 4 |
| 39881 | 2 | 2 | 1 | 0 | 4 | 3 | 1 | 1 | 1 | 2 | 3 | 2 | 3 | 1 | 4 | 2 | 2 | 2 | 4 | 1 | 4 | 3 | 3 | 3 | 4 | 1 | 1 | 1 | 2 | 3 | 3 | 3 | 4 |
| 39881 | 3 | 2 | 1 | 0 | 4 | 4 | 1 | 1 | 1 | 2 | 4 | 2 | 3 | 1 | 4 | 2 | 2 | 2 | 3 | 1 | 4 | 4 | 4 | 3 | 4 | 1 | 1 | 1 | 2 | 3 | 3 | 3 | 3 |
| 39881 | 4 | 2 | 1 | 1 | 3 | 4 | 2 | 1 | 1 | 2 | 3 | 2 | 3 | 1 | 4 | 2 | 2 | 2 | 3 | 2 | 4 | 3 | 3 | 3 | 3 | 1 | 2 | 2 | 2 | 3 | 3 | 3 | 3 |
| 39881 | 5 | 2 | 2 | 1 | 2 | 4 | 3 | 2 | 2 | 3 | 2 | 2 | 4 | 2 | 4 | 4 | 3 | 2 | 3 | 2 | 4 | 3 | 3 | 4 | 4 | 4 | 4 | 3 | 2 | 2 | 2 | 4 | 3 |
| 39921 | 1 | 2 | 2 | 2 | 3 | 2 | 4 | 2 | 4 | 3 | 3 | 4 | 3 | 2 | 2 | 2 | 3 | 2 | 3 | 3 | 2 | 3 | 3 | 3 | 3 | 3 | 2 | 2 | 2 | 2 | 2 | 2 | 2 |
| 39921 | 2 | 2 | 2 | 2 | 3 | 2 | 4 | 2 | 3 | 3 | 3 | 4 | 3 | 2 | 2 | 2 | 3 | 2 | 3 | 3 | 2 | 3 | 3 | 3 | 3 | 3 | 2 | 2 | 2 | 2 | 2 | 2 | 2 |
| 39921 | 3 | 2 | 2 | 2 | 3 | 2 | 4 | 2 | 3 | 3 | 3 | 4 | 4 | 2 | 2 | 2 | 3 | 2 | 3 | 3 | 2 | 3 | 3 | 2 | 3 | 3 | 2 | 2 | 2 | 2 | 2 | 2 | 2 |
| 39921 | 4 | 4 | 4 | 4 | 4 | 4 | 4 | 4 | 4 | 3 | 3 | 4 | 4 | 4 | 4 | 4 | 4 | 3 | 3 | 3 | 3 | 3 | 4 | 4 | 4 | 4 | 4 | 3 | 3 | 3 | 2 | 3 | 3 |
| 39921 | 5 | 2 | 2 | 2 | 3 | 2 | 3 | 2 | 3 | 3 | 3 | 4 | 3 | 2 | 2 | 2 | 3 | 2 | 3 | 3 | 2 | 3 | 3 | 3 | 3 | 3 | 2 | 2 | 2 | 2 | 2 | 2 | 2 |
| 39925 | 3 | 3 | 3 | 4 | 3 | 3 | 4 | 3 | 3 | 3 | 3 | 4 | 4 | 3 | 3 | 3 | 2 | 3 | 3 | 3 | 3 | 3 | 3 | 3 | 3 | 3 | 2 | 3 | 3 | 3 | 2 | 3 | 2 |
| 39925 | 4 | 3 | 4 | 3 | 4 | 4 | 4 | 3 | 3 | 3 | 3 | 4 | 3 | 3 | 3 | 3 | 1 | 3 | 3 | 3 | 3 | 3 | 3 | 3 | 3 | 3 | 2 | 3 | 3 | 3 | 2 | 3 | 2 |
| 39943 | 1 | 2 | 2 | 2 | 2 | 2 | 1 | 2 | 2 | 1 | 2 | 2 | 2 | 2 | 2 | 2 | 2 | 2 | 2 | 2 | 2 | 1 | 2 | 2 | 2 | 2 | 2 | 2 | 2 | 2 | 2 | 2 | 2 |
| 39943 | 2 | 3 | 3 | 3 | 3 | 3 | 3 | 3 | 3 | 2 | 3 | 2 | 3 | 3 | 3 | 2 | 3 | 3 | 3 | 3 | 3 | 3 | 3 | 3 | 3 | 3 | 3 | 3 | 2 | 2 | 2 | 2 | 2 |
| 39943 | 3 | 2 | 2 | 1 | 2 | 2 | 2 | 2 | 2 | 2 | 2 | 2 | 2 | 2 | 2 | 2 | 2 | 2 | 2 | 2 | 2 | 2 | 2 | 2 | 2 | 2 | 2 | 2 | 2 | 2 | 2 | 2 | 2 |
| 39943 | 4 | 3 | 3 | 3 | 3 | 3 | 3 | 3 | 4 | 2 | 3 | 2 | 4 | 3 | 3 | 2 | 3 | 3 | 3 | 3 | 3 | 3 | 3 | 3 | 3 | 3 | 3 | 3 | 2 | 2 | 2 | 2 | 2 |
| 39944 | 1 | 2 | 3 | 2 | 4 | 3 | 3 | 3 | 3 | 3 | 4 | 3 | 3 | 2 | 3 | 3 | 3 | 3 | 2 | 3 | 3 | 3 | 3 | 3 | 3 | 3 | 3 | 2 | 3 | 3 | 2 | 3 | 3 |
| 39944 | 2 | 2 | 3 | 2 | 4 | 3 | 3 | 4 | 3 | 3 | 4 | 3 | 3 | 2 | 3 | 3 | 3 | 3 | 2 | 3 | 3 | 3 | 3 | 3 | 3 | 3 | 3 | 2 | 3 | 3 | 2 | 3 | 3 |
| 39944 | 3 | 2 | 3 | 2 | 4 | 3 | 3 | 4 | 3 | 3 | 4 | 3 | 3 | 2 | 3 | 3 | 3 | 3 | 2 | 3 | 3 | 3 | 3 | 3 | 3 | 3 | 3 | 2 | 3 | 3 | 2 | 3 | 3 |
| 39944 | 4 | 2 | 3 | 2 | 4 | 3 | 3 | 4 | 3 | 3 | 4 | 3 | 3 | 2 | 3 | 3 | 3 | 3 | 2 | 3 | 3 | 3 | 3 | 3 | 3 | 3 | 3 | 2 | 3 | 3 | 2 | 3 | 3 |
| 39944 | 5 | 2 | 4 | 2 | 4 | 3 | 3 | 4 | 3 | 3 | 4 | 3 | 3 | 2 | 3 | 3 | 3 | 3 | 2 | 3 | 3 | 3 | 3 | 3 | 3 | 3 | 3 | 2 | 3 | 3 | 2 | 3 | 3 |
| 39959 | 2 | 2 | 2 | 2 | 2 | 2 | 2 | 2 | 2 | 2 | 2 | 2 | 2 | 2 | 2 | 2 | 2 | 2 | 2 | 2 | 2 | 1 | 1 | 2 | 2 | 2 | 2 | 2 | 2 | 2 | 2 | 0 | 2 |
| 39959 | 3 | 2 | 1 | 1 | 1 | 1 | 3 | 2 | 3 | 3 | 2 | 2 | 3 | 2 | 3 | 3 | 2 | 2 | 2 | 2 | 3 | 3 | 0 | 3 | 2 | 2 | 3 | 3 | 3 | 4 | 3 | 4 | 3 |
| 39959 | 4 | 2 | 2 | 0 | 1 | 1 | 3 | 2 | 3 | 3 | 2 | 2 | 3 | 2 | 3 | 3 | 2 | 2 | 2 | 2 | 3 | 3 | 1 | 3 | 2 | 2 | 3 | 3 | 3 | 4 | 4 | 3 | 3 |
| 39984 | 1 | 0 | 0 | 0 | 1 | 0 | 4 | 0 | 1 | 0 | 4 | 0 | 4 | 1 | 1 | 0 | 1 | 1 | 1 | 4 | 1 | 3 | 3 | 2 | 2 | 4 | 4 | 1 | 2 | 3 | 2 | 3 | 2 |
| 39985 | 1 | 3 | 2 | 2 | 4 | 3 | 3 | 3 | 3 | 3 | 3 | 3 | 3 | 3 | 3 | 3 | 2 | 3 | 3 | 4 | 3 | 3 | 4 | 2 | 4 | 3 | 3 | 3 | 3 | 3 | 3 | 3 | 2 |
| 39985 | 3 | 1 | 1 | 1 | 2 | 2 | 4 | 1 | 2 | 2 | 2 | 1 | 3 | 2 | 2 | 2 | 1 | 2 | 2 | 3 | 2 | 2 | 3 | 2 | 2 | 4 | 3 | 2 | 2 | 3 | 2 | 3 | 2 |
| 39985 | 5 | 1 | 1 | 1 | 1 | 2 | 3 | 2 | 2 | 2 | 2 | 2 | 4 | 2 | 2 | 1 | 1 | 2 | 2 | 3 | 2 | 2 | 3 | 2 | 2 | 4 | 3 | 2 | 1 | 3 | 2 | 4 | 2 |
| 39986 | 1 | 0 | 1 | 0 | 2 | 0 | 0 | 0 | 1 | 2 | 0 | 1 | 1 | 1 | 0 | 0 | 0 | 0 | 0 | 1 | 0 | 0 | 2 | 2 | 0 | 0 | 0 | 0 | 2 | 0 | 3 | 2 | 2 |
| 39986 | 2 | 3 | 2 | 0 | 2 | 2 | 3 | 2 | 3 | 3 | 2 | 3 | 2 | 2 | 3 | 3 | 3 | 2 | 2 | 3 | 3 | 3 | 3 | 2 | 3 | 3 | 3 | 3 | 3 | 2 | 3 | 2 | 3 |
| 39986 | 3 | 3 | 2 | 0 | 2 | 1 | 3 | 2 | 3 | 3 | 2 | 3 | 2 | 2 | 3 | 3 | 3 | 2 | 2 | 3 | 3 | 3 | 3 | 0 | 3 | 3 | 3 | 3 | 3 | 2 | 3 | 2 | 4 |
| 39986 | 4 | 0 | 0 | 0 | 0 | 2 | 0 | 0 | 1 | 1 | 1 | 1 | 0 | 1 | 0 | 1 | 0 | 0 | 1 | 0 | 0 | 0 | 2 | 0 | 0 | 0 | 0 | 0 | 2 | 0 | 4 | 2 | 2 |
| 39986 | 5 | 0 | 1 | 0 | 1 | 1 | 0 | 1 | 1 | 2 | 1 | 1 | 1 | 1 | 0 | 1 | 0 | 0 | 0 | 0 | 0 | 0 | 2 | 1 | 0 | 0 | 0 | 0 | 3 | 3 | 3 | 2 | 3 |
| 39987 | 1 | 3 | 3 | 1 | 3 | 4 | 4 | 4 | 4 | 3 | 4 | 3 | 4 | 4 | 3 | 3 | 3 | 3 | 3 | 4 | 4 | 3 | 4 | 3 | 3 | 4 | 3 | 3 | 3 | 2 | 2 | 2 | 2 |
| 39987 | 2 | 2 | 2 | 2 | 2 | 3 | 3 | 3 | 3 | 3 | 3 | 3 | 3 | 3 | 3 | 3 | 2 | 2 | 2 | 3 | 2 | 3 | 4 | 3 | 3 | 2 | 2 | 2 | 3 | 3 | 2 | 2 | 2 |
| 39987 | 3 | 4 | 3 | 1 | 3 | 4 | 4 | 4 | 4 | 3 | 3 | 3 | 3 | 4 | 3 | 3 | 3 | 3 | 3 | 3 | 3 | 3 | 3 | 3 | 3 | 4 | 3 | 3 | 3 | 2 | 2 | 2 | 2 |
| 39987 | 4 | 2 | 2 | 2 | 2 | 3 | 3 | 3 | 3 | 3 | 3 | 3 | 3 | 3 | 3 | 3 | 2 | 2 | 2 | 3 | 2 | 3 | 3 | 3 | 3 | 2 | 2 | 2 | 3 | 3 | 2 | 2 | 2 |
| 39987 | 5 | 3 | 4 | 1 | 4 | 4 | 4 | 4 | 4 | 3 | 4 | 4 | 4 | 4 | 4 | 3 | 4 | 4 | 3 | 3 | 3 | 4 | 4 | 4 | 3 | 4 | 3 | 3 | 3 | 2 | 2 | 2 | 2 |
| 39991 | 1 | 0 | 0 | 1 | 0 | 0 | 2 | 0 | 1 | 3 | 0 | 3 | 3 | 3 | 3 | 3 | 2 | 0 | 3 | 0 | 3 | 3 | 0 | 3 | 3 | 3 | 2 | 3 | 2 | 2 | 1 | 2 | 1 |
| 39992 | 1 | 3 | 4 | 3 | 4 | 3 | 3 | 3 | 3 | 3 | 4 | 3 | 3 | 4 | 3 | 4 | 3 | 3 | 3 | 3 | 3 | 3 | 3 | 3 | 3 | 3 | 3 | 3 | 3 | 3 | 3 | 3 | 3 |
| 39992 | 2 | 3 | 3 | 3 | 4 | 4 | 3 | 3 | 3 | 3 | 4 | 3 | 3 | 4 | 4 | 4 | 3 | 3 | 3 | 3 | 3 | 3 | 3 | 3 | 3 | 3 | 3 | 3 | 3 | 3 | 3 | 3 | 3 |
| 39992 | 3 | 3 | 4 | 3 | 4 | 4 | 3 | 3 | 3 | 3 | 3 | 3 | 3 | 4 | 4 | 4 | 3 | 3 | 3 | 3 | 3 | 3 | 3 | 3 | 3 | 3 | 3 | 3 | 3 | 3 | 3 | 3 | 3 |
| 39992 | 4 | 3 | 3 | 3 | 4 | 4 | 3 | 4 | 3 | 3 | 4 | 3 | 3 | 4 | 4 | 4 | 3 | 3 | 3 | 3 | 3 | 3 | 3 | 3 | 3 | 3 | 3 | 3 | 3 | 3 | 3 | 3 | 3 |
| 39992 | 5 | 2 | 2 | 1 | 2 | 0 | 3 | 2 | 0 | 3 | 0 | 3 | 3 | 2 | 3 | 3 | 3 | 3 | 3 | 3 | 3 | 2 | 2 | 0 | 3 | 3 | 2 | 3 | 3 | 4 | 3 | 3 | 4 |
| 39993 | 1 | 0 | 0 | 0 | 0 | 0 | 0 | 0 | 0 | 0 | 0 | 0 | 0 | 0 | 1 | 0 | 0 | 0 | 0 | 0 | 0 | 0 | 0 | 0 | 1 | 0 | 1 | 0 | 3 | 3 | 1 | 3 | 3 |
| 39993 | 2 | 1 | 0 | 2 | 1 | 2 | 2 | 1 | 2 | 2 | 1 | 2 | 2 | 2 | 2 | 1 | 1 | 1 | 2 | 2 | 2 | 2 | 2 | 2 | 2 | 1 | 2 | 1 | 2 | 2 | 2 | 2 | 2 |
| 39993 | 3 | 2 | 2 | 0 | 2 | 2 | 2 | 2 | 2 | 2 | 2 | 2 | 2 | 2 | 2 | 2 | 2 | 2 | 2 | 2 | 2 | 2 | 2 | 2 | 2 | 2 | 2 | 2 | 2 | 2 | 2 | 2 | 2 |
| 39993 | 4 | 2 | 2 | 0 | 2 | 2 | 2 | 2 | 2 | 2 | 2 | 2 | 2 | 2 | 2 | 2 | 2 | 2 | 2 | 2 | 2 | 2 | 2 | 2 | 2 | 2 | 2 | 2 | 2 | 2 | 2 | 2 | 2 |
| 39993 | 5 | 0 | 1 | 0 | 0 | 0 | 0 | 0 | 0 | 0 | 0 | 0 | 0 | 0 | 0 | 1 | 0 | 0 | 0 | 0 | 0 | 1 | 0 | 0 | 0 | 0 | 1 | 0 | 3 | 3 | 1 | 3 | 3 |
| 39994 | 1 | 1 | 1 | 0 | 1 | 1 | 1 | 1 | 1 | 1 | 2 | 1 | 1 | 2 | 1 | 1 | 1 | 1 | 1 | 2 | 1 | 2 | 2 | 1 | 1 | 2 | 2 | 1 | 1 | 2 | 1 | 2 | 1 |
| 39994 | 2 | 0 | 0 | 1 | 1 | 1 | 2 | 1 | 1 | 1 | 2 | 1 | 2 | 1 | 1 | 1 | 1 | 0 | 0 | 2 | 1 | 2 | 2 | 1 | 1 | 2 | 2 | 1 | 1 | 2 | 1 | 2 | 1 |
| 39994 | 3 | 1 | 0 | 0 | 1 | 1 | 2 | 1 | 1 | 1 | 2 | 2 | 2 | 1 | 1 | 1 | 1 | 1 | 1 | 2 | 1 | 2 | 2 | 1 | 1 | 2 | 2 | 1 | 1 | 2 | 1 | 2 | 1 |
| 39994 | 4 | 1 | 0 | 1 | 1 | 1 | 2 | 1 | 2 | 1 | 2 | 1 | 2 | 1 | 1 | 1 | 1 | 1 | 1 | 2 | 1 | 2 | 2 | 1 | 1 | 2 | 2 | 1 | 1 | 2 | 1 | 2 | 1 |
| 39997 | 1 | 2 | 2 | 2 | 2 | 2 | 1 | 2 | 2 | 2 | 2 | 2 | 2 | 2 | 2 | 2 | 1 | 2 | 2 | 2 | 2 | 2 | 2 | 2 | 2 | 2 | 2 | 2 | 3 | 3 | 3 | 2 | 3 |
| 39997 | 2 | 2 | 2 | 2 | 2 | 2 | 2 | 2 | 2 | 2 | 2 | 2 | 2 | 2 | 2 | 2 | 2 | 2 | 2 | 2 | 2 | 2 | 2 | 2 | 2 | 2 | 2 | 2 | 3 | 3 | 4 | 2 | 3 |
| 39997 | 3 | 2 | 2 | 0 | 2 | 2 | 1 | 1 | 2 | 2 | 2 | 2 | 2 | 2 | 2 | 2 | 2 | 1 | 1 | 2 | 2 | 2 | 2 | 2 | 2 | 2 | 2 | 1 | 2 | 2 | 2 | 2 | 2 |
| 39997 | 4 | 2 | 2 | 2 | 2 | 2 | 1 | 2 | 2 | 2 | 2 | 2 | 2 | 2 | 2 | 2 | 2 | 2 | 2 | 2 | 2 | 2 | 2 | 2 | 2 | 2 | 2 | 2 | 3 | 3 | 3 | 2 | 3 |
| 39997 | 5 | 2 | 2 | 1 | 2 | 2 | 1 | 2 | 2 | 2 | 2 | 2 | 2 | 2 | 2 | 2 | 2 | 1 | 1 | 2 | 2 | 2 | 2 | 1 | 2 | 2 | 2 | 2 | 2 | 2 | 2 | 2 | 2 |
| 39998 | 1 | 1 | 1 | 2 | 2 | 2 | 2 | 1 | 2 | 2 | 2 | 1 | 1 | 2 | 1 | 1 | 1 | 1 | 2 | 1 | 2 | 2 | 2 | 2 | 1 | 0 | 2 | 1 | 2 | 3 | 3 | 3 | 3 |
| 39998 | 2 | 2 | 2 | 2 | 2 | 2 | 2 | 2 | 2 | 2 | 2 | 2 | 2 | 2 | 2 | 2 | 2 | 2 | 2 | 2 | 2 | 2 | 2 | 2 | 2 | 2 | 2 | 2 | 2 | 3 | 3 | 3 | 3 |
| 39998 | 3 | 0 | 0 | 0 | 0 | 0 | 0 | 0 | 0 | 0 | 0 | 1 | 1 | 0 | 1 | 0 | 0 | 0 | 0 | 0 | 0 | 1 | 0 | 1 | 1 | 0 | 1 | 0 | 2 | 1 | 2 | 2 | 2 |
| 39998 | 4 | 2 | 2 | 2 | 2 | 2 | 2 | 1 | 2 | 2 | 2 | 1 | 1 | 2 | 2 | 2 | 1 | 1 | 2 | 1 | 2 | 2 | 2 | 2 | 2 | 2 | 2 | 1 | 2 | 3 | 3 | 3 | 3 |
| 39998 | 5 | 2 | 2 | 2 | 2 | 2 | 2 | 2 | 2 | 2 | 2 | 2 | 2 | 2 | 2 | 2 | 2 | 2 | 2 | 2 | 2 | 2 | 2 | 2 | 2 | 2 | 2 | 2 | 2 | 3 | 3 | 3 | 3 |
| 39999 | 1 | 2 | 1 | 0 | 2 | 0 | 0 | 1 | 2 | 0 | 0 | 0 | 0 | 0 | 0 | 0 | 0 | 1 | 0 | 0 | 1 | 0 | 0 | 0 | 0 | 1 | 0 | 0 | 2 | 2 | 2 | 2 | 2 |
| 39999 | 2 | 2 | 2 | 1 | 2 | 0 | 0 | 1 | 2 | 0 | 0 | 0 | 0 | 0 | 0 | 0 | 0 | 0 | 0 | 0 | 1 | 0 | 0 | 0 | 0 | 0 | 0 | 0 | 2 | 2 | 2 | 2 | 2 |
| 39999 | 3 | 3 | 1 | 0 | 2 | 1 | 2 | 2 | 1 | 2 | 1 | 3 | 1 | 1 | 3 | 3 | 3 | 0 | 1 | 1 | 3 | 3 | 3 | 1 | 3 | 3 | 2 | 2 | 2 | 2 | 2 | 2 | 2 |
| 39999 | 4 | 3 | 1 | 0 | 1 | 1 | 2 | 2 | 1 | 2 | 2 | 3 | 1 | 1 | 3 | 3 | 3 | 1 | 1 | 2 | 3 | 3 | 3 | 2 | 3 | 3 | 2 | 2 | 2 | 2 | 2 | 2 | 2 |
| 39999 | 5 | 1 | 1 | 0 | 2 | 0 | 0 | 1 | 2 | 0 | 0 | 0 | 0 | 0 | 0 | 0 | 0 | 0 | 0 | 0 | 1 | 0 | 0 | 0 | 0 | 0 | 0 | 0 | 2 | 2 | 2 | 2 | 2 |
| 40001 | 1 | 2 | 2 | 1 | 1 | 1 | 2 | 1 | 2 | 1 | 2 | 1 | 1 | 2 | 1 | 2 | 1 | 1 | 1 | 2 | 2 | 2 | 1 | 1 | 1 | 2 | 2 | 1 | 2 | 2 | 2 | 2 | 2 |
| 40001 | 2 | 1 | 1 | 0 | 1 | 1 | 2 | 2 | 2 | 1 | 1 | 2 | 2 | 1 | 2 | 2 | 0 | 1 | 2 | 2 | 2 | 2 | 1 | 1 | 2 | 1 | 2 | 0 | 2 | 2 | 0 | 2 | 0 |
| 40001 | 3 | 2 | 3 | 2 | 1 | 2 | 3 | 2 | 2 | 2 | 2 | 2 | 2 | 3 | 2 | 2 | 2 | 2 | 2 | 2 | 2 | 2 | 2 | 2 | 1 | 2 | 3 | 2 | 3 | 3 | 3 | 3 | 3 |
| 40001 | 4 | 2 | 3 | 0 | 1 | 1 | 3 | 2 | 2 | 2 | 2 | 1 | 2 | 3 | 0 | 2 | 2 | 1 | 1 | 2 | 1 | 2 | 2 | 2 | 1 | 2 | 3 | 2 | 3 | 3 | 3 | 3 | 3 |
| 40001 | 5 | 2 | 2 | 0 | 2 | 1 | 2 | 2 | 2 | 2 | 1 | 1 | 2 | 0 | 2 | 2 | 2 | 2 | 2 | 2 | 2 | 2 | 2 | 2 | 2 | 2 | 2 | 2 | 2 | 2 | 2 | 2 | 2 |
| 40003 | 1 | 1 | 1 | 0 | 1 | 1 | 1 | 2 | 2 | 1 | 1 | 2 | 1 | 2 | 1 | 2 | 1 | 1 | 1 | 2 | 2 | 2 | 2 | 2 | 2 | 1 | 1 | 1 | 2 | 2 | 2 | 2 | 2 |
| 40003 | 2 | 1 | 1 | 0 | 1 | 1 | 1 | 2 | 2 | 1 | 0 | 2 | 2 | 2 | 1 | 2 | 1 | 1 | 2 | 1 | 2 | 2 | 2 | 2 | 2 | 2 | 1 | 1 | 2 | 2 | 2 | 2 | 2 |
| 40003 | 3 | 1 | 1 | 0 | 2 | 1 | 1 | 2 | 2 | 1 | 1 | 2 | 2 | 2 | 1 | 2 | 1 | 1 | 2 | 2 | 2 | 2 | 2 | 2 | 2 | 1 | 2 | 1 | 2 | 2 | 2 | 2 | 2 |
| 40003 | 4 | 1 | 1 | 0 | 1 | 0 | 1 | 0 | 2 | 2 | 1 | 1 | 0 | 1 | 2 | 2 | 2 | 0 | 1 | 2 | 2 | 1 | 1 | 0 | 0 | 2 | 2 | 2 | 2 | 2 | 2 | 2 | 2 |
| 40003 | 5 | 1 | 1 | 0 | 1 | 1 | 1 | 2 | 2 | 2 | 1 | 2 | 2 | 2 | 2 | 2 | 1 | 2 | 2 | 2 | 2 | 2 | 2 | 2 | 1 | 2 | 1 | 1 | 2 | 2 | 2 | 2 | 2 |
| 40006 | 1 | 1 | 0 | 2 | 1 | 2 | 2 | 0 | 1 | 3 | 3 | 3 | 2 | 3 | 2 | 1 | 1 | 1 | 3 | 2 | 3 | 2 | 3 | 3 | 2 | 2 | 2 | 2 | 3 | 3 | 3 | 3 | 3 |
| 40006 | 2 | 1 | 0 | 2 | 0 | 2 | 2 | 1 | 2 | 3 | 3 | 3 | 2 | 3 | 2 | 0 | 2 | 1 | 3 | 2 | 3 | 2 | 3 | 3 | 2 | 2 | 2 | 2 | 3 | 3 | 3 | 3 | 3 |
| 40006 | 3 | 2 | 1 | 2 | 1 | 2 | 2 | 0 | 2 | 3 | 3 | 3 | 2 | 3 | 2 | 0 | 2 | 2 | 3 | 2 | 3 | 2 | 3 | 3 | 2 | 1 | 2 | 2 | 3 | 3 | 3 | 3 | 3 |
| 40006 | 4 | 2 | 0 | 2 | 1 | 2 | 2 | 0 | 1 | 3 | 3 | 3 | 2 | 3 | 2 | 0 | 2 | 2 | 3 | 2 | 3 | 1 | 3 | 3 | 2 | 1 | 2 | 2 | 3 | 3 | 3 | 3 | 3 |
| 40006 | 5 | 2 | 1 | 2 | 0 | 2 | 2 | 0 | 1 | 3 | 3 | 3 | 2 | 3 | 2 | 0 | 2 | 2 | 3 | 2 | 3 | 0 | 3 | 3 | 2 | 2 | 2 | 2 | 3 | 3 | 3 | 3 | 3 |
| 40007 | 1 | 0 | 0 | 0 | 1 | 2 | 1 | 1 | 1 | 2 | 1 | 1 | 1 | 2 | 2 | 0 | 1 | 1 | 1 | 2 | 2 | 1 | 1 | 1 | 1 | 1 | 1 | 1 | 2 | 2 | 2 | 2 | 2 |
| 40007 | 2 | 0 | 0 | 0 | 1 | 1 | 2 | 1 | 1 | 2 | 1 | 2 | 1 | 2 | 2 | 0 | 1 | 1 | 1 | 1 | 2 | 1 | 1 | 2 | 1 | 1 | 1 | 1 | 2 | 2 | 2 | 2 | 1 |
| 40007 | 3 | 1 | 1 | 2 | 2 | 1 | 2 | 1 | 0 | 2 | 2 | 2 | 2 | 2 | 2 | 1 | 2 | 1 | 2 | 2 | 2 | 2 | 2 | 2 | 2 | 1 | 1 | 1 | 2 | 2 | 2 | 2 | 2 |
| 40007 | 4 | 1 | 1 | 0 | 1 | 1 | 1 | 1 | 1 | 2 | 2 | 1 | 2 | 2 | 2 | 0 | 2 | 1 | 2 | 2 | 2 | 1 | 2 | 2 | 1 | 1 | 1 | 1 | 2 | 2 | 2 | 2 | 2 |
| 40007 | 5 | 1 | 0 | 0 | 1 | 1 | 2 | 1 | 1 | 1 | 2 | 1 | 2 | 2 | 2 | 0 | 2 | 1 | 1 | 2 | 2 | 1 | 2 | 2 | 1 | 1 | 1 | 1 | 2 | 2 | 2 | 2 | 2 |
| 40011 | 1 | 1 | 1 | 0 | 3 | 0 | 2 | 2 | 2 | 2 | 2 | 2 | 2 | 2 | 3 | 2 | 2 | 2 | 2 | 3 | 3 | 2 | 2 | 2 | 2 | 2 | 2 | 1 | 2 | 2 | 2 | 2 | 2 |
| 40011 | 2 | 1 | 1 | 0 | 3 | 0 | 2 | 2 | 2 | 2 | 2 | 2 | 2 | 2 | 3 | 2 | 2 | 2 | 2 | 3 | 3 | 2 | 2 | 2 | 2 | 2 | 2 | 1 | 2 | 2 | 2 | 2 | 2 |
| 40011 | 3 | 2 | 1 | 0 | 3 | 0 | 2 | 2 | 2 | 2 | 2 | 2 | 2 | 2 | 3 | 2 | 2 | 2 | 2 | 3 | 3 | 2 | 2 | 2 | 2 | 2 | 2 | 1 | 2 | 2 | 2 | 2 | 2 |
| 40011 | 4 | 1 | 1 | 0 | 3 | 0 | 2 | 2 | 2 | 2 | 2 | 2 | 2 | 2 | 3 | 2 | 2 | 2 | 2 | 3 | 3 | 2 | 2 | 2 | 2 | 2 | 2 | 1 | 2 | 2 | 2 | 2 | 2 |
| 40011 | 5 | 1 | 1 | 0 | 3 | 0 | 2 | 2 | 2 | 2 | 2 | 2 | 2 | 2 | 3 | 2 | 2 | 2 | 2 | 3 | 3 | 2 | 2 | 2 | 2 | 2 | 2 | 0 | 2 | 2 | 2 | 2 | 2 |
| 40013 | 1 | 1 | 1 | 0 | 3 | 1 | 2 | 1 | 2 | 2 | 3 | 2 | 2 | 3 | 3 | 3 | 3 | 1 | 2 | 3 | 2 | 2 | 2 | 3 | 2 | 2 | 2 | 1 | 2 | 2 | 2 | 0 | 0 |
| 40013 | 2 | 2 | 2 | 2 | 3 | 2 | 2 | 2 | 2 | 2 | 3 | 2 | 2 | 3 | 3 | 3 | 3 | 2 | 2 | 3 | 2 | 2 | 2 | 3 | 2 | 2 | 2 | 2 | 2 | 2 | 2 | 2 | 2 |
| 40013 | 3 | 2 | 2 | 0 | 3 | 0 | 0 | 2 | 2 | 2 | 3 | 2 | 2 | 3 | 3 | 3 | 3 | 2 | 2 | 3 | 2 | 2 | 2 | 3 | 2 | 2 | 2 | 2 | 2 | 2 | 2 | 2 | 2 |
| 40013 | 4 | 1 | 1 | 2 | 3 | 1 | 2 | 1 | 2 | 2 | 3 | 2 | 2 | 3 | 3 | 3 | 3 | 1 | 2 | 3 | 2 | 2 | 2 | 3 | 2 | 2 | 2 | 0 | 2 | 2 | 2 | 2 | 2 |
| 40013 | 5 | 2 | 2 | 2 | 3 | 2 | 2 | 2 | 2 | 2 | 3 | 2 | 2 | 3 | 3 | 3 | 3 | 2 | 2 | 3 | 2 | 2 | 2 | 3 | 2 | 2 | 2 | 2 | 2 | 2 | 2 | 2 | 2 |
| 40017 | 1 | 3 | 3 | 3 | 4 | 4 | 4 | 3 | 3 | 2 | 3 | 4 | 3 | 3 | 3 | 3 | 3 | 2 | 4 | 4 | 4 | 3 | 4 | 3 | 3 | 3 | 3 | 3 | 2 | 2 | 3 | 3 | 3 |
| 40017 | 2 | 0 | 0 | 0 | 0 | 0 | 0 | 0 | 0 | 1 | 0 | 2 | 2 | 0 | 0 | 0 | 2 | 0 | 0 | 0 | 2 | 2 | 0 | 2 | 2 | 0 | 2 | 2 | 2 | 2 | 2 | 0 | 0 |
| 40017 | 3 | 4 | 3 | 3 | 3 | 4 | 4 | 3 | 3 | 2 | 3 | 4 | 4 | 4 | 3 | 3 | 3 | 2 | 3 | 3 | 4 | 3 | 4 | 3 | 3 | 4 | 3 | 3 | 2 | 2 | 3 | 3 | 3 |
| 40017 | 4 | 4 | 3 | 3 | 4 | 4 | 4 | 3 | 3 | 2 | 3 | 4 | 4 | 3 | 3 | 3 | 4 | 2 | 4 | 3 | 4 | 3 | 3 | 3 | 4 | 4 | 4 | 4 | 2 | 2 | 3 | 3 | 3 |
| 40017 | 5 | 4 | 4 | 3 | 4 | 4 | 4 | 3 | 3 | 2 | 3 | 4 | 4 | 3 | 3 | 4 | 3 | 2 | 4 | 3 | 4 | 3 | 3 | 3 | 4 | 3 | 3 | 4 | 2 | 2 | 3 | 3 | 3 |
| 40018 | 1 | 2 | 2 | 2 | 2 | 2 | 2 | 2 | 2 | 2 | 2 | 2 | 2 | 2 | 2 | 0 | 2 | 2 | 2 | 2 | 2 | 2 | 2 | 2 | 2 | 0 | 2 | 2 | 2 | 3 | 3 | 3 | 3 |
| 40018 | 2 | 2 | 0 | 1 | 0 | 1 | 0 | 2 | 1 | 1 | 1 | 1 | 1 | 1 | 2 | 0 | 1 | 1 | 1 | 2 | 2 | 0 | 2 | 0 | 0 | 0 | 1 | 0 | 2 | 3 | 3 | 3 | 3 |
| 40018 | 3 | 0 | 2 | 2 | 2 | 1 | 1 | 1 | 1 | 1 | 1 | 1 | 1 | 1 | 2 | 1 | 1 | 1 | 1 | 2 | 2 | 1 | 2 | 1 | 1 | 1 | 1 | 0 | 2 | 2 | 2 | 2 | 2 |
| 40018 | 4 | 0 | 2 | 2 | 1 | 1 | 1 | 1 | 1 | 1 | 1 | 1 | 1 | 1 | 2 | 1 | 2 | 1 | 2 | 2 | 2 | 1 | 2 | 1 | 1 | 0 | 1 | 1 | 2 | 2 | 2 | 2 | 2 |
| 40018 | 5 | 2 | 2 | 2 | 2 | 2 | 2 | 2 | 2 | 2 | 2 | 2 | 2 | 2 | 2 | 2 | 2 | 2 | 2 | 2 | 2 | 2 | 2 | 2 | 2 | 0 | 2 | 2 | 2 | 2 | 2 | 2 | 2 |
| 40025 | 1 | 2 | 0 | 0 | 1 | 1 | 3 | 1 | 2 | 2 | 1 | 3 | 2 | 2 | 2 | 2 | 3 | 1 | 2 | 2 | 3 | 2 | 1 | 2 | 2 | 1 | 2 | 2 | 2 | 2 | 2 | 2 | 2 |
| 40025 | 2 | 1 | 1 | 0 | 1 | 1 | 3 | 1 | 2 | 2 | 1 | 3 | 2 | 2 | 2 | 2 | 3 | 1 | 2 | 1 | 3 | 2 | 1 | 2 | 2 | 1 | 2 | 2 | 2 | 2 | 2 | 2 | 2 |
| 40025 | 3 | 1 | 1 | 0 | 1 | 1 | 3 | 1 | 2 | 2 | 1 | 3 | 2 | 2 | 2 | 2 | 3 | 1 | 2 | 1 | 3 | 2 | 1 | 2 | 2 | 1 | 2 | 2 | 2 | 2 | 2 | 2 | 2 |
| 40025 | 4 | 1 | 1 | 0 | 1 | 1 | 3 | 1 | 2 | 2 | 1 | 3 | 2 | 2 | 2 | 2 | 3 | 1 | 2 | 1 | 3 | 2 | 1 | 2 | 2 | 1 | 2 | 2 | 2 | 2 | 2 | 2 | 2 |
| 40025 | 5 | 1 | 1 | 0 | 0 | 1 | 3 | 1 | 2 | 2 | 1 | 3 | 2 | 2 | 2 | 1 | 3 | 1 | 2 | 0 | 3 | 2 | 0 | 2 | 2 | 0 | 2 | 2 | 2 | 2 | 2 | 2 | 2 |
| 40030 | 1 | 1 | 1 | 1 | 1 | 1 | 1 | 1 | 1 | 1 | 0 | 1 | 1 | 1 | 1 | 1 | 1 | 1 | 1 | 1 | 0 | 1 | 1 | 1 | 1 | 0 | 1 | 0 | 2 | 3 | 1 | 2 | 2 |
| 40030 | 2 | 1 | 1 | 0 | 1 | 1 | 1 | 1 | 1 | 0 | 1 | 0 | 1 | 1 | 1 | 1 | 1 | 1 | 1 | 1 | 1 | 1 | 0 | 2 | 0 | 0 | 1 | 0 | 2 | 3 | 2 | 2 | 2 |
| 40030 | 3 | 2 | 2 | 2 | 2 | 1 | 2 | 2 | 2 | 2 | 2 | 2 | 2 | 2 | 2 | 2 | 2 | 2 | 2 | 2 | 2 | 2 | 2 | 2 | 2 | 2 | 2 | 2 | 2 | 3 | 2 | 2 | 2 |
| 40030 | 4 | 1 | 1 | 1 | 1 | 1 | 1 | 1 | 1 | 1 | 1 | 1 | 1 | 1 | 1 | 1 | 1 | 1 | 1 | 1 | 0 | 1 | 1 | 1 | 0 | 1 | 1 | 0 | 2 | 4 | 2 | 2 | 1 |
| 40030 | 5 | 0 | 4 | 0 | 0 | 0 | 2 | 0 | 3 | 4 | 3 | 2 | 2 | 2 | 3 | 3 | 3 | 2 | 3 | 2 | 4 | 4 | 2 | 2 | 3 | 4 | 3 | 4 | 3 | 4 | 3 | 0 | 0 |
| 40032 | 1 | 1 | 1 | 1 | 1 | 1 | 3 | 1 | 2 | 2 | 3 | 2 | 3 | 2 | 1 | 2 | 2 | 2 | 2 | 3 | 2 | 3 | 3 | 2 | 2 | 3 | 2 | 2 | 2 | 4 | 2 | 3 | 2 |
| 40032 | 2 | 1 | 1 | 0 | 1 | 1 | 3 | 1 | 2 | 2 | 3 | 2 | 3 | 2 | 2 | 1 | 2 | 2 | 2 | 3 | 2 | 3 | 3 | 1 | 2 | 3 | 2 | 2 | 2 | 3 | 2 | 3 | 2 |
| 40032 | 3 | 4 | 4 | 4 | 4 | 4 | 4 | 4 | 4 | 4 | 4 | 4 | 4 | 4 | 4 | 4 | 4 | 4 | 4 | 3 | 4 | 4 | 4 | 4 | 4 | 4 | 4 | 4 | 4 | 4 | 4 | 4 | 4 |
| 40032 | 4 | 4 | 4 | 3 | 4 | 3 | 4 | 4 | 4 | 4 | 3 | 4 | 4 | 4 | 4 | 4 | 4 | 3 | 4 | 4 | 4 | 4 | 4 | 4 | 3 | 3 | 4 | 4 | 4 | 4 | 4 | 4 | 4 |
| 40032 | 5 | 4 | 4 | 4 | 4 | 4 | 4 | 4 | 4 | 4 | 4 | 4 | 4 | 3 | 4 | 4 | 4 | 4 | 3 | 4 | 4 | 4 | 4 | 4 | 3 | 4 | 4 | 4 | 4 | 4 | 4 | 4 | 4 |
| 40036 | 1 | 2 | 1 | 2 | 2 | 0 | 2 | 2 | 2 | 2 | 2 | 2 | 2 | 2 | 2 | 2 | 2 | 2 | 2 | 2 | 2 | 2 | 2 | 0 | 2 | 2 | 0 | 2 | 0 | 4 | 4 | 4 | 4 |
| 40036 | 2 | 2 | 3 | 2 | 3 | 4 | 2 | 4 | 3 | 2 | 3 | 3 | 3 | 3 | 3 | 3 | 3 | 3 | 3 | 3 | 4 | 3 | 3 | 3 | 3 | 3 | 2 | 3 | 3 | 3 | 3 | 2 | 3 |
| 40036 | 3 | 2 | 4 | 2 | 4 | 4 | 2 | 4 | 3 | 2 | 3 | 3 | 3 | 4 | 3 | 3 | 3 | 3 | 3 | 3 | 3 | 3 | 4 | 3 | 3 | 3 | 2 | 3 | 3 | 3 | 3 | 2 | 3 |
| 40036 | 4 | 2 | 4 | 2 | 4 | 4 | 2 | 4 | 4 | 2 | 3 | 3 | 4 | 3 | 3 | 3 | 4 | 4 | 3 | 3 | 4 | 3 | 4 | 4 | 4 | 3 | 2 | 3 | 3 | 3 | 3 | 2 | 3 |
| 40038 | 1 | 3 | 4 | 3 | 4 | 3 | 4 | 4 | 4 | 4 | 4 | 4 | 3 | 4 | 4 | 3 | 4 | 4 | 4 | 4 | 4 | 3 | 4 | 3 | 3 | 4 | 4 | 4 | 4 | 4 | 4 | 4 | 3 |
| 40038 | 2 | 4 | 4 | 3 | 4 | 3 | 4 | 4 | 4 | 4 | 4 | 4 | 3 | 4 | 4 | 4 | 4 | 4 | 4 | 4 | 4 | 4 | 4 | 3 | 4 | 4 | 4 | 4 | 3 | 4 | 4 | 4 | 4 |
| 40038 | 3 | 3 | 4 | 3 | 4 | 3 | 3 | 4 | 3 | 3 | 3 | 4 | 3 | 3 | 3 | 3 | 3 | 4 | 4 | 4 | 3 | 3 | 3 | 3 | 3 | 3 | 3 | 3 | 3 | 3 | 3 | 3 | 3 |
| 40038 | 4 | 3 | 4 | 3 | 3 | 4 | 3 | 3 | 3 | 3 | 3 | 4 | 3 | 3 | 3 | 3 | 4 | 3 | 3 | 3 | 3 | 3 | 3 | 3 | 3 | 3 | 3 | 3 | 3 | 3 | 3 | 3 | 3 |
| 40038 | 5 | 3 | 3 | 3 | 3 | 3 | 3 | 3 | 4 | 3 | 3 | 4 | 3 | 3 | 3 | 4 | 4 | 3 | 3 | 3 | 3 | 3 | 3 | 4 | 3 | 3 | 3 | 3 | 3 | 3 | 3 | 3 | 3 |
| 40040 | 1 | 4 | 4 | 0 | 4 | 4 | 4 | 4 | 4 | 3 | 4 | 3 | 4 | 4 | 4 | 4 | 4 | 4 | 3 | 3 | 4 | 4 | 4 | 4 | 4 | 4 | 4 | 3 | 3 | 3 | 3 | 3 | 3 |
| 40040 | 2 | 4 | 4 | 2 | 4 | 4 | 4 | 4 | 4 | 4 | 4 | 4 | 4 | 4 | 4 | 4 | 4 | 4 | 3 | 4 | 4 | 4 | 4 | 4 | 4 | 4 | 4 | 4 | 4 | 4 | 3 | 3 | 3 |
| 40040 | 3 | 4 | 4 | 0 | 4 | 4 | 4 | 4 | 4 | 3 | 3 | 4 | 4 | 3 | 4 | 4 | 4 | 4 | 3 | 3 | 4 | 3 | 3 | 4 | 3 | 3 | 3 | 3 | 4 | 4 | 3 | 3 | 3 |
| 40040 | 4 | 4 | 4 | 0 | 4 | 4 | 4 | 4 | 4 | 3 | 3 | 4 | 4 | 4 | 3 | 4 | 4 | 4 | 3 | 3 | 3 | 3 | 3 | 3 | 3 | 3 | 3 | 3 | 3 | 3 | 3 | 3 | 3 |
| 40040 | 5 | 4 | 4 | 0 | 4 | 4 | 4 | 4 | 4 | 3 | 3 | 4 | 3 | 4 | 3 | 4 | 4 | 3 | 3 | 3 | 3 | 3 | 3 | 3 | 3 | 3 | 3 | 3 | 3 | 3 | 3 | 3 | 3 |
| 40042 | 1 | 3 | 4 | 0 | 3 | 3 | 3 | 3 | 3 | 3 | 3 | 3 | 3 | 3 | 3 | 3 | 3 | 3 | 3 | 3 | 3 | 3 | 3 | 3 | 3 | 3 | 3 | 3 | 3 | 3 | 3 | 3 | 3 |
| 40042 | 2 | 4 | 4 | 2 | 4 | 4 | 3 | 4 | 3 | 3 | 4 | 4 | 4 | 4 | 4 | 4 | 4 | 4 | 3 | 3 | 4 | 4 | 4 | 4 | 3 | 4 | 3 | 3 | 4 | 4 | 4 | 3 | 4 |
| 40042 | 3 | 4 | 4 | 2 | 3 | 3 | 4 | 3 | 3 | 3 | 3 | 3 | 3 | 4 | 3 | 3 | 4 | 3 | 3 | 3 | 3 | 3 | 3 | 3 | 3 | 3 | 3 | 3 | 3 | 4 | 3 | 3 | 3 |
| 40042 | 4 | 4 | 4 | 2 | 4 | 4 | 3 | 3 | 4 | 3 | 4 | 3 | 4 | 4 | 4 | 4 | 4 | 4 | 3 | 4 | 3 | 4 | 4 | 4 | 3 | 4 | 4 | 4 | 4 | 3 | 3 | 3 | 3 |
| 40042 | 5 | 4 | 4 | 2 | 4 | 4 | 3 | 4 | 3 | 3 | 4 | 4 | 4 | 4 | 4 | 4 | 4 | 3 | 3 | 3 | 3 | 3 | 4 | 4 | 3 | 4 | 3 | 3 | 4 | 4 | 4 | 3 | 3 |
| 40044 | 1 | 2 | 2 | 0 | 1 | 1 | 2 | 2 | 2 | 2 | 2 | 0 | 1 | 2 | 0 | 2 | 2 | 1 | 0 | 2 | 0 | 0 | 0 | 0 | 0 | 2 | 2 | 2 | 4 | 4 | 4 | 3 | 4 |
| 40044 | 2 | 2 | 2 | 2 | 0 | 0 | 4 | 2 | 2 | 3 | 3 | 0 | 0 | 3 | 0 | 2 | 2 | 0 | 0 | 2 | 0 | 0 | 0 | 0 | 0 | 2 | 2 | 3 | 4 | 3 | 3 | 3 | 3 |
| 40044 | 3 | 2 | 2 | 2 | 0 | 0 | 3 | 2 | 2 | 3 | 3 | 0 | 0 | 3 | 0 | 2 | 2 | 0 | 0 | 2 | 0 | 0 | 0 | 0 | 0 | 2 | 2 | 3 | 4 | 3 | 4 | 3 | 3 |
| 40044 | 4 | 2 | 2 | 0 | 0 | 0 | 2 | 2 | 2 | 2 | 2 | 0 | 0 | 2 | 0 | 2 | 2 | 0 | 0 | 2 | 0 | 0 | 0 | 0 | 0 | 2 | 2 | 2 | 4 | 4 | 4 | 3 | 4 |
| 40044 | 5 | 2 | 2 | 2 | 0 | 0 | 3 | 3 | 2 | 3 | 4 | 0 | 1 | 2 | 0 | 3 | 2 | 0 | 0 | 4 | 0 | 0 | 0 | 0 | 0 | 4 | 4 | 3 | 4 | 4 | 4 | 3 | 4 |
| 40048 | 1 | 3 | 3 | 0 | 3 | 3 | 3 | 3 | 3 | 3 | 3 | 3 | 3 | 4 | 3 | 3 | 3 | 3 | 3 | 3 | 3 | 3 | 3 | 3 | 4 | 3 | 3 | 3 | 3 | 4 | 3 | 3 | 3 |
| 40048 | 2 | 3 | 3 | 2 | 3 | 4 | 4 | 3 | 3 | 3 | 4 | 3 | 4 | 3 | 3 | 4 | 3 | 3 | 3 | 3 | 4 | 4 | 3 | 4 | 4 | 4 | 4 | 4 | 3 | 4 | 3 | 3 | 3 |
| 40048 | 3 | 3 | 3 | 2 | 3 | 3 | 3 | 3 | 3 | 3 | 3 | 3 | 4 | 3 | 3 | 4 | 3 | 3 | 3 | 3 | 3 | 4 | 4 | 4 | 4 | 4 | 4 | 4 | 3 | 4 | 3 | 3 | 3 |
| 40048 | 4 | 3 | 3 | 2 | 3 | 3 | 3 | 4 | 4 | 3 | 3 | 3 | 4 | 4 | 3 | 3 | 4 | 3 | 3 | 3 | 3 | 4 | 4 | 3 | 4 | 4 | 4 | 4 | 3 | 4 | 3 | 4 | 3 |
| 40048 | 5 | 3 | 3 | 0 | 3 | 3 | 3 | 3 | 3 | 3 | 4 | 3 | 3 | 3 | 3 | 3 | 4 | 2 | 3 | 3 | 3 | 3 | 4 | 4 | 3 | 4 | 4 | 4 | 3 | 3 | 3 | 3 | 3 |
| 40066 | 1 | 3 | 4 | 4 | 3 | 3 | 4 | 3 | 3 | 3 | 3 | 3 | 3 | 3 | 3 | 3 | 4 | 3 | 3 | 3 | 3 | 4 | 4 | 4 | 4 | 3 | 3 | 3 | 3 | 3 | 2 | 3 | 3 |
| 40066 | 2 | 3 | 4 | 4 | 3 | 3 | 4 | 3 | 4 | 3 | 3 | 4 | 4 | 4 | 3 | 4 | 4 | 3 | 3 | 3 | 4 | 4 | 4 | 4 | 3 | 4 | 3 | 3 | 3 | 3 | 2 | 3 | 3 |
| 40066 | 3 | 3 | 4 | 4 | 4 | 3 | 4 | 4 | 4 | 3 | 3 | 3 | 3 | 4 | 3 | 4 | 4 | 3 | 3 | 3 | 4 | 3 | 4 | 3 | 3 | 4 | 3 | 3 | 3 | 3 | 2 | 3 | 3 |
| 40066 | 4 | 3 | 4 | 4 | 4 | 4 | 4 | 3 | 3 | 3 | 3 | 4 | 3 | 4 | 3 | 3 | 4 | 3 | 4 | 3 | 4 | 4 | 4 | 4 | 4 | 4 | 3 | 3 | 3 | 3 | 2 | 3 | 3 |
| 40066 | 5 | 3 | 3 | 4 | 4 | 4 | 4 | 3 | 3 | 3 | 3 | 4 | 3 | 3 | 3 | 3 | 4 | 3 | 4 | 3 | 4 | 4 | 4 | 4 | 3 | 4 | 3 | 3 | 3 | 3 | 2 | 3 | 3 |
| 40076 | 1 | 3 | 2 | 2 | 4 | 2 | 2 | 2 | 2 | 3 | 3 | 3 | 3 | 2 | 3 | 2 | 2 | 3 | 3 | 2 | 4 | 3 | 3 | 2 | 3 | 2 | 2 | 2 | 2 | 3 | 3 | 3 | 3 |
| 40076 | 2 | 2 | 1 | 1 | 2 | 2 | 2 | 1 | 2 | 2 | 2 | 2 | 2 | 2 | 2 | 2 | 2 | 2 | 2 | 2 | 2 | 2 | 2 | 2 | 2 | 2 | 2 | 2 | 2 | 2 | 2 | 2 | 2 |
| 40076 | 3 | 2 | 1 | 1 | 2 | 2 | 2 | 1 | 2 | 2 | 2 | 2 | 2 | 2 | 2 | 2 | 2 | 2 | 2 | 2 | 2 | 2 | 2 | 2 | 2 | 1 | 2 | 2 | 2 | 2 | 2 | 2 | 2 |
| 40076 | 4 | 2 | 2 | 2 | 2 | 2 | 4 | 1 | 4 | 4 | 2 | 3 | 4 | 3 | 4 | 4 | 4 | 2 | 3 | 2 | 4 | 4 | 2 | 4 | 4 | 2 | 4 | 4 | 4 | 4 | 4 | 4 | 4 |
| 40076 | 5 | 2 | 1 | 1 | 2 | 2 | 2 | 1 | 2 | 2 | 2 | 2 | 2 | 2 | 2 | 2 | 2 | 2 | 2 | 2 | 2 | 2 | 2 | 2 | 2 | 2 | 2 | 2 | 2 | 2 | 2 | 2 | 2 |
| 40095 | 1 | 3 | 4 | 3 | 3 | 4 | 4 | 3 | 4 | 3 | 3 | 4 | 4 | 4 | 4 | 4 | 4 | 4 | 4 | 3 | 4 | 4 | 4 | 4 | 4 | 4 | 4 | 4 | 4 | 4 | 4 | 4 | 4 |
| 40095 | 2 | 4 | 4 | 4 | 4 | 4 | 4 | 4 | 4 | 3 | 4 | 4 | 4 | 4 | 3 | 4 | 4 | 4 | 4 | 4 | 4 | 4 | 4 | 4 | 4 | 4 | 4 | 3 | 4 | 4 | 3 | 4 | 4 |
| 40095 | 3 | 4 | 4 | 3 | 4 | 3 | 4 | 4 | 4 | 3 | 3 | 3 | 4 | 4 | 4 | 4 | 4 | 3 | 4 | 4 | 4 | 4 | 4 | 4 | 4 | 4 | 4 | 4 | 4 | 4 | 3 | 4 | 4 |
| 40095 | 4 | 3 | 4 | 3 | 4 | 4 | 4 | 4 | 4 | 3 | 3 | 3 | 4 | 4 | 3 | 4 | 4 | 3 | 3 | 4 | 4 | 3 | 4 | 4 | 3 | 4 | 4 | 3 | 3 | 3 | 3 | 3 | 3 |
| 40095 | 5 | 3 | 4 | 4 | 4 | 4 | 4 | 3 | 4 | 3 | 3 | 3 | 4 | 3 | 3 | 4 | 4 | 3 | 3 | 4 | 4 | 4 | 4 | 4 | 4 | 4 | 4 | 3 | 3 | 3 | 3 | 3 | 3 |
| 40096 | 1 | 3 | 3 | 3 | 3 | 3 | 3 | 3 | 4 | 3 | 3 | 4 | 3 | 3 | 3 | 4 | 3 | 3 | 4 | 3 | 3 | 3 | 3 | 4 | 3 | 3 | 3 | 3 | 4 | 4 | 3 | 3 | 3 |
| 40096 | 2 | 3 | 4 | 4 | 4 | 4 | 4 | 4 | 4 | 3 | 4 | 3 | 4 | 4 | 3 | 4 | 4 | 3 | 4 | 4 | 4 | 4 | 4 | 4 | 4 | 3 | 3 | 3 | 4 | 4 | 3 | 3 | 4 |
| 40096 | 3 | 2 | 2 | 2 | 2 | 2 | 2 | 1 | 2 | 2 | 2 | 2 | 2 | 2 | 2 | 2 | 2 | 2 | 2 | 2 | 2 | 2 | 2 | 2 | 2 | 2 | 2 | 2 | 2 | 2 | 2 | 2 | 2 |
| 40096 | 4 | 3 | 4 | 4 | 4 | 4 | 4 | 4 | 4 | 3 | 4 | 4 | 4 | 4 | 4 | 4 | 4 | 4 | 4 | 4 | 4 | 4 | 4 | 4 | 4 | 4 | 4 | 4 | 4 | 4 | 3 | 4 | 4 |
| 40096 | 5 | 3 | 3 | 3 | 3 | 3 | 3 | 3 | 3 | 3 | 3 | 3 | 3 | 4 | 3 | 4 | 3 | 3 | 3 | 3 | 3 | 3 | 3 | 3 | 3 | 3 | 3 | 3 | 4 | 4 | 3 | 4 | 3 |
| 40160 | 1 | 0 | 0 | 2 | 0 | 0 | 0 | 0 | 0 | 3 | 0 | 3 | 0 | 0 | 1 | 4 | 4 | 0 | 0 | 0 | 4 | 3 | 0 | 0 | 4 | 4 | 3 | 3 | 3 | 3 | 3 | 2 | 0 |
| 40160 | 2 | 0 | 0 | 2 | 0 | 0 | 0 | 0 | 0 | 3 | 0 | 3 | 0 | 1 | 1 | 3 | 4 | 0 | 0 | 0 | 4 | 4 | 0 | 0 | 4 | 3 | 3 | 3 | 3 | 3 | 3 | 2 | 0 |
| 40160 | 3 | 3 | 3 | 3 | 4 | 4 | 4 | 4 | 4 | 4 | 4 | 4 | 4 | 4 | 4 | 4 | 4 | 4 | 4 | 4 | 4 | 4 | 3 | 4 | 4 | 4 | 4 | 4 | 3 | 4 | 3 | 3 | 4 |
| 40160 | 4 | 2 | 2 | 2 | 2 | 2 | 2 | 2 | 2 | 3 | 2 | 2 | 3 | 3 | 3 | 2 | 2 | 3 | 3 | 2 | 2 | 2 | 3 | 3 | 2 | 2 | 2 | 2 | 2 | 4 | 3 | 3 | 3 |
| 40160 | 5 | 2 | 2 | 3 | 2 | 2 | 2 | 3 | 2 | 2 | 3 | 2 | 3 | 3 | 2 | 3 | 3 | 3 | 3 | 2 | 2 | 2 | 3 | 2 | 3 | 2 | 2 | 2 | 2 | 3 | 3 | 3 | 2 |
| 40163 | 1 | 4 | 4 | 3 | 4 | 4 | 4 | 4 | 4 | 4 | 4 | 4 | 4 | 4 | 3 | 4 | 4 | 4 | 4 | 4 | 4 | 4 | 4 | 4 | 4 | 4 | 3 | 4 | 3 | 3 | 3 | 3 | 3 |
| 40163 | 2 | 3 | 4 | 4 | 4 | 4 | 4 | 3 | 4 | 4 | 4 | 4 | 4 | 4 | 4 | 4 | 3 | 4 | 4 | 4 | 4 | 3 | 4 | 4 | 4 | 4 | 4 | 3 | 3 | 3 | 2 | 2 | 2 |
| 40163 | 3 | 3 | 4 | 4 | 4 | 4 | 4 | 4 | 4 | 4 | 4 | 4 | 4 | 4 | 4 | 4 | 3 | 4 | 4 | 4 | 4 | 4 | 4 | 4 | 4 | 4 | 3 | 3 | 3 | 3 | 2 | 2 | 2 |
| 40163 | 4 | 4 | 4 | 4 | 4 | 4 | 4 | 4 | 4 | 3 | 3 | 4 | 4 | 3 | 3 | 4 | 4 | 3 | 3 | 4 | 4 | 4 | 3 | 4 | 4 | 4 | 3 | 4 | 3 | 4 | 3 | 4 | 3 |
| 40163 | 5 | 2 | 2 | 4 | 4 | 4 | 2 | 2 | 3 | 2 | 4 | 2 | 2 | 2 | 2 | 2 | 2 | 3 | 2 | 4 | 2 | 2 | 4 | 2 | 2 | 2 | 2 | 2 | 3 | 4 | 4 | 4 | 3 |
| 40165 | 1 | 2 | 3 | 4 | 3 | 3 | 4 | 3 | 3 | 3 | 3 | 2 | 3 | 3 | 2 | 2 | 3 | 4 | 3 | 3 | 3 | 3 | 4 | 3 | 3 | 3 | 2 | 3 | 2 | 4 | 2 | 3 | 2 |
| 40165 | 2 | 3 | 4 | 4 | 4 | 4 | 4 | 4 | 4 | 4 | 4 | 4 | 4 | 4 | 4 | 4 | 4 | 4 | 4 | 4 | 4 | 4 | 4 | 4 | 4 | 4 | 4 | 4 | 3 | 3 | 3 | 3 | 3 |
| 40165 | 3 | 4 | 4 | 3 | 4 | 4 | 4 | 4 | 4 | 4 | 4 | 4 | 4 | 4 | 4 | 3 | 4 | 4 | 4 | 4 | 4 | 4 | 4 | 4 | 3 | 4 | 4 | 4 | 3 | 4 | 3 | 3 | 3 |
| 40165 | 4 | 2 | 3 | 4 | 3 | 3 | 4 | 3 | 3 | 3 | 3 | 2 | 3 | 3 | 2 | 2 | 3 | 3 | 3 | 3 | 3 | 3 | 3 | 3 | 3 | 3 | 2 | 3 | 2 | 3 | 2 | 3 | 2 |
| 40165 | 5 | 4 | 3 | 2 | 4 | 4 | 4 | 4 | 4 | 4 | 4 | 4 | 4 | 4 | 4 | 4 | 4 | 4 | 4 | 4 | 4 | 4 | 4 | 4 | 3 | 4 | 4 | 4 | 3 | 4 | 3 | 3 | 3 |
| 40168 | 1 | 2 | 2 | 2 | 2 | 2 | 2 | 2 | 2 | 2 | 2 | 2 | 2 | 2 | 2 | 2 | 2 | 2 | 2 | 2 | 2 | 2 | 2 | 2 | 2 | 2 | 2 | 2 | 2 | 3 | 3 | 4 | 3 |
| 40168 | 2 | 2 | 2 | 2 | 2 | 2 | 2 | 2 | 2 | 2 | 2 | 2 | 2 | 2 | 2 | 2 | 2 | 2 | 2 | 2 | 2 | 2 | 2 | 2 | 2 | 2 | 2 | 2 | 2 | 3 | 3 | 4 | 3 |
| 40168 | 3 | 2 | 2 | 2 | 2 | 2 | 2 | 2 | 2 | 2 | 2 | 2 | 2 | 2 | 2 | 2 | 2 | 2 | 2 | 2 | 2 | 2 | 2 | 2 | 2 | 2 | 2 | 2 | 2 | 3 | 3 | 4 | 3 |
| 40168 | 4 | 2 | 2 | 2 | 2 | 2 | 2 | 2 | 2 | 2 | 2 | 2 | 2 | 2 | 2 | 2 | 2 | 2 | 2 | 2 | 2 | 2 | 2 | 2 | 2 | 2 | 2 | 2 | 2 | 3 | 3 | 3 | 3 |
| 40168 | 5 | 2 | 2 | 2 | 2 | 2 | 2 | 2 | 2 | 2 | 2 | 2 | 2 | 2 | 2 | 2 | 2 | 2 | 2 | 2 | 2 | 2 | 2 | 2 | 2 | 2 | 2 | 2 | 2 | 3 | 3 | 3 | 3 |
| 40169 | 1 | 2 | 2 | 0 | 2 | 2 | 2 | 2 | 2 | 2 | 2 | 2 | 2 | 2 | 2 | 2 | 2 | 2 | 2 | 2 | 2 | 2 | 2 | 2 | 2 | 3 | 2 | 2 | 2 | 3 | 3 | 2 | 2 |
| 40169 | 2 | 2 | 2 | 0 | 1 | 2 | 1 | 0 | 2 | 2 | 2 | 2 | 2 | 2 | 2 | 2 | 2 | 2 | 2 | 2 | 2 | 2 | 2 | 2 | 2 | 3 | 2 | 2 | 2 | 3 | 3 | 2 | 2 |
| 40169 | 3 | 2 | 3 | 0 | 1 | 0 | 3 | 1 | 3 | 3 | 2 | 0 | 0 | 3 | 0 | 3 | 3 | 0 | 0 | 3 | 0 | 0 | 0 | 0 | 0 | 3 | 3 | 3 | 2 | 3 | 2 | 2 | 2 |
| 40169 | 4 | 1 | 2 | 0 | 1 | 2 | 2 | 1 | 2 | 2 | 2 | 2 | 2 | 2 | 2 | 2 | 2 | 2 | 2 | 2 | 2 | 2 | 2 | 2 | 2 | 3 | 2 | 2 | 2 | 3 | 3 | 2 | 2 |
| 40169 | 5 | 2 | 2 | 0 | 1 | 2 | 2 | 1 | 2 | 2 | 2 | 2 | 2 | 2 | 2 | 2 | 2 | 2 | 2 | 2 | 2 | 2 | 2 | 2 | 2 | 3 | 2 | 2 | 2 | 3 | 3 | 2 | 2 |
| 40172 | 1 | 2 | 2 | 3 | 2 | 3 | 3 | 2 | 3 | 3 | 2 | 3 | 3 | 3 | 2 | 2 | 3 | 3 | 2 | 3 | 3 | 3 | 3 | 2 | 2 | 3 | 3 | 3 | 2 | 3 | 3 | 3 | 3 |
| 40176 | 1 | 2 | 3 | 2 | 2 | 3 | 3 | 4 | 2 | 3 | 2 | 2 | 2 | 3 | 2 | 3 | 3 | 2 | 2 | 2 | 3 | 3 | 2 | 2 | 2 | 3 | 2 | 2 | 3 | 3 | 3 | 3 | 3 |
| 40176 | 2 | 2 | 3 | 2 | 2 | 3 | 3 | 3 | 2 | 3 | 2 | 2 | 2 | 3 | 2 | 3 | 3 | 2 | 2 | 2 | 3 | 3 | 2 | 2 | 2 | 3 | 2 | 2 | 3 | 3 | 3 | 3 | 3 |
| 40176 | 3 | 2 | 4 | 0 | 1 | 3 | 3 | 3 | 2 | 3 | 2 | 2 | 2 | 3 | 2 | 3 | 3 | 2 | 2 | 2 | 3 | 3 | 2 | 2 | 2 | 3 | 2 | 2 | 3 | 3 | 3 | 3 | 3 |
| 40176 | 4 | 2 | 3 | 2 | 2 | 3 | 3 | 3 | 2 | 3 | 2 | 2 | 2 | 3 | 2 | 3 | 3 | 2 | 2 | 2 | 3 | 3 | 2 | 2 | 2 | 3 | 2 | 2 | 3 | 3 | 3 | 3 | 3 |
| 40176 | 5 | 3 | 3 | 2 | 3 | 3 | 3 | 3 | 2 | 3 | 2 | 2 | 2 | 3 | 2 | 3 | 3 | 2 | 2 | 2 | 3 | 3 | 2 | 3 | 2 | 3 | 2 | 2 | 3 | 3 | 3 | 3 | 3 |
| 40185 | 1 | 2 | 2 | 2 | 2 | 2 | 2 | 2 | 2 | 2 | 2 | 2 | 2 | 2 | 2 | 2 | 2 | 2 | 2 | 2 | 3 | 3 | 2 | 2 | 2 | 3 | 2 | 2 | 3 | 3 | 3 | 3 | 3 |
| 40185 | 2 | 2 | 2 | 2 | 2 | 2 | 2 | 2 | 2 | 2 | 2 | 2 | 2 | 2 | 2 | 2 | 2 | 2 | 2 | 2 | 3 | 3 | 2 | 2 | 2 | 3 | 2 | 2 | 3 | 3 | 3 | 3 | 3 |
| 40185 | 3 | 2 | 2 | 2 | 2 | 2 | 2 | 2 | 2 | 2 | 2 | 2 | 2 | 2 | 2 | 2 | 2 | 2 | 2 | 2 | 3 | 3 | 2 | 2 | 2 | 3 | 2 | 2 | 3 | 4 | 3 | 3 | 3 |
| 40185 | 4 | 2 | 2 | 2 | 2 | 2 | 2 | 2 | 2 | 2 | 2 | 2 | 2 | 2 | 2 | 2 | 2 | 2 | 2 | 2 | 3 | 3 | 2 | 2 | 2 | 3 | 2 | 2 | 3 | 3 | 3 | 3 | 3 |
| 40185 | 5 | 2 | 2 | 2 | 2 | 2 | 2 | 2 | 2 | 2 | 2 | 2 | 2 | 2 | 2 | 2 | 2 | 2 | 2 | 2 | 3 | 3 | 2 | 2 | 2 | 3 | 2 | 2 | 3 | 4 | 3 | 3 | 3 |
| 40189 | 1 | 3 | 3 | 4 | 3 | 2 | 3 | 3 | 3 | 3 | 2 | 3 | 3 | 3 | 3 | 3 | 2 | 3 | 3 | 2 | 4 | 4 | 3 | 3 | 3 | 3 | 2 | 3 | 3 | 4 | 3 | 3 | 3 |
| 40189 | 2 | 3 | 3 | 3 | 3 | 2 | 3 | 3 | 3 | 3 | 2 | 3 | 3 | 3 | 3 | 3 | 2 | 3 | 3 | 2 | 3 | 3 | 3 | 3 | 3 | 3 | 2 | 3 | 3 | 4 | 3 | 3 | 3 |
| 40189 | 3 | 3 | 3 | 3 | 3 | 2 | 3 | 3 | 3 | 3 | 2 | 3 | 3 | 3 | 3 | 3 | 0 | 3 | 3 | 2 | 3 | 3 | 3 | 3 | 3 | 3 | 2 | 3 | 3 | 3 | 3 | 3 | 3 |
| 40189 | 4 | 3 | 4 | 3 | 3 | 2 | 3 | 3 | 3 | 3 | 2 | 3 | 3 | 3 | 3 | 3 | 2 | 3 | 3 | 2 | 3 | 3 | 3 | 3 | 3 | 3 | 2 | 3 | 3 | 4 | 3 | 3 | 3 |
| 40189 | 5 | 3 | 3 | 3 | 3 | 2 | 3 | 3 | 3 | 3 | 2 | 3 | 3 | 3 | 3 | 3 | 2 | 3 | 3 | 2 | 3 | 3 | 3 | 3 | 3 | 3 | 0 | 3 | 3 | 4 | 3 | 3 | 3 |
| 40190 | 1 | 2 | 3 | 2 | 2 | 2 | 3 | 2 | 3 | 3 | 3 | 2 | 3 | 3 | 2 | 2 | 2 | 3 | 2 | 2 | 3 | 2 | 2 | 3 | 3 | 3 | 2 | 3 | 3 | 3 | 3 | 4 | 4 |
| 40190 | 2 | 3 | 3 | 3 | 0 | 2 | 3 | 2 | 3 | 3 | 3 | 3 | 3 | 3 | 3 | 3 | 3 | 3 | 3 | 3 | 4 | 3 | 3 | 3 | 0 | 3 | 2 | 3 | 3 | 3 | 3 | 3 | 3 |
| 40190 | 3 | 4 | 3 | 4 | 4 | 4 | 4 | 4 | 3 | 3 | 4 | 3 | 4 | 4 | 3 | 3 | 4 | 2 | 3 | 3 | 4 | 3 | 3 | 3 | 3 | 3 | 2 | 3 | 3 | 3 | 3 | 3 | 3 |
| 40190 | 4 | 3 | 3 | 3 | 3 | 3 | 4 | 3 | 3 | 3 | 3 | 3 | 3 | 3 | 3 | 3 | 3 | 2 | 3 | 3 | 3 | 3 | 3 | 3 | 3 | 3 | 2 | 3 | 3 | 4 | 3 | 3 | 3 |
| 40190 | 5 | 3 | 4 | 3 | 3 | 3 | 3 | 3 | 3 | 3 | 3 | 3 | 3 | 3 | 3 | 3 | 3 | 2 | 3 | 3 | 3 | 3 | 3 | 3 | 3 | 3 | 2 | 3 | 3 | 4 | 3 | 3 | 3 |
| 40192 | 1 | 2 | 3 | 3 | 3 | 3 | 3 | 3 | 3 | 3 | 3 | 3 | 3 | 3 | 3 | 3 | 3 | 3 | 3 | 3 | 3 | 3 | 3 | 3 | 3 | 3 | 3 | 3 | 3 | 4 | 3 | 3 | 3 |
| 40192 | 2 | 4 | 4 | 4 | 4 | 4 | 4 | 4 | 3 | 4 | 4 | 4 | 4 | 4 | 4 | 3 | 4 | 4 | 3 | 4 | 4 | 3 | 4 | 4 | 4 | 4 | 3 | 4 | 3 | 4 | 3 | 3 | 4 |
| 40192 | 3 | 2 | 3 | 2 | 3 | 2 | 2 | 2 | 3 | 3 | 3 | 2 | 3 | 3 | 3 | 3 | 2 | 3 | 3 | 3 | 3 | 3 | 2 | 2 | 3 | 3 | 3 | 3 | 3 | 3 | 3 | 3 | 3 |
| 40192 | 4 | 2 | 3 | 3 | 3 | 3 | 3 | 3 | 3 | 3 | 3 | 3 | 3 | 3 | 3 | 3 | 2 | 3 | 3 | 3 | 3 | 3 | 3 | 3 | 3 | 3 | 3 | 3 | 3 | 3 | 3 | 3 | 3 |
| 40192 | 5 | 2 | 3 | 3 | 3 | 3 | 3 | 3 | 3 | 3 | 3 | 3 | 3 | 3 | 3 | 3 | 3 | 3 | 3 | 3 | 3 | 3 | 3 | 3 | 3 | 3 | 3 | 3 | 3 | 3 | 3 | 4 | 3 |
| 110734 | 1 | 2 | 3 | 3 | 3 | 3 | 3 | 3 | 3 | 3 | 3 | 3 | 3 | 3 | 3 | 3 | 3 | 3 | 3 | 3 | 3 | 3 | 3 | 3 | 3 | 3 | 3 | 3 | 3 | 4 | 3 | 3 | 3 |
| 110734 | 2 | 2 | 3 | 3 | 3 | 4 | 3 | 3 | 3 | 3 | 3 | 3 | 4 | 3 | 3 | 3 | 3 | 3 | 3 | 3 | 3 | 3 | 3 | 3 | 3 | 3 | 3 | 3 | 3 | 4 | 3 | 3 | 3 |
| 110734 | 3 | 4 | 4 | 2 | 4 | 3 | 4 | 3 | 3 | 3 | 3 | 3 | 4 | 4 | 3 | 3 | 3 | 3 | 3 | 3 | 3 | 3 | 3 | 3 | 3 | 3 | 3 | 3 | 3 | 4 | 3 | 3 | 3 |
| 110734 | 4 | 3 | 3 | 0 | 4 | 4 | 4 | 4 | 4 | 4 | 3 | 4 | 4 | 4 | 4 | 4 | 3 | 4 | 4 | 4 | 3 | 3 | 4 | 3 | 4 | 4 | 4 | 4 | 3 | 3 | 3 | 3 | 3 |
| 110734 | 5 | 2 | 3 | 3 | 3 | 3 | 3 | 3 | 3 | 3 | 3 | 3 | 4 | 3 | 3 | 3 | 3 | 3 | 3 | 3 | 3 | 3 | 3 | 3 | 3 | 3 | 3 | 3 | 3 | 3 | 3 | 3 | 3 |
| 110736 | 1 | 2 | 2 | 2 | 2 | 3 | 2 | 2 | 2 | 2 | 3 | 2 | 2 | 2 | 2 | 2 | 3 | 2 | 2 | 3 | 3 | 3 | 3 | 3 | 2 | 3 | 3 | 3 | 3 | 3 | 3 | 3 | 3 |
| 110736 | 2 | 2 | 2 | 2 | 2 | 3 | 2 | 2 | 2 | 2 | 3 | 2 | 2 | 2 | 2 | 2 | 3 | 2 | 2 | 3 | 3 | 3 | 3 | 3 | 2 | 3 | 3 | 3 | 3 | 3 | 3 | 3 | 3 |
| 110736 | 3 | 3 | 4 | 3 | 2 | 3 | 3 | 2 | 3 | 3 | 2 | 3 | 3 | 3 | 3 | 3 | 4 | 3 | 3 | 3 | 4 | 4 | 3 | 3 | 3 | 3 | 3 | 3 | 3 | 3 | 3 | 3 | 4 |
| 110736 | 4 | 3 | 3 | 3 | 2 | 3 | 3 | 2 | 3 | 3 | 2 | 3 | 3 | 3 | 3 | 3 | 3 | 3 | 3 | 3 | 3 | 3 | 3 | 3 | 3 | 3 | 3 | 3 | 3 | 3 | 3 | 3 | 3 |
| 110736 | 5 | 2 | 4 | 2 | 1 | 1 | 2 | 2 | 2 | 2 | 2 | 2 | 1 | 2 | 1 | 2 | 2 | 1 | 1 | 3 | 2 | 2 | 1 | 2 | 1 | 3 | 2 | 3 | 3 | 3 | 3 | 3 | 3 |
| 110759 | 1 | 2 | 3 | 2 | 2 | 2 | 2 | 2 | 2 | 2 | 2 | 2 | 2 | 2 | 2 | 3 | 2 | 2 | 2 | 3 | 2 | 2 | 2 | 2 | 2 | 3 | 2 | 3 | 3 | 3 | 2 | 3 | 3 |
| 110759 | 2 | 3 | 3 | 4 | 3 | 3 | 4 | 3 | 3 | 3 | 3 | 3 | 3 | 3 | 3 | 3 | 3 | 3 | 3 | 3 | 3 | 3 | 3 | 3 | 3 | 3 | 3 | 3 | 3 | 4 | 3 | 3 | 3 |
| 110759 | 3 | 2 | 3 | 2 | 2 | 2 | 2 | 2 | 2 | 2 | 2 | 2 | 2 | 2 | 2 | 3 | 2 | 2 | 2 | 3 | 2 | 2 | 2 | 2 | 2 | 3 | 2 | 3 | 3 | 3 | 2 | 3 | 3 |
| 110759 | 4 | 2 | 3 | 2 | 2 | 2 | 2 | 2 | 2 | 2 | 2 | 2 | 2 | 2 | 2 | 3 | 2 | 2 | 2 | 3 | 2 | 2 | 2 | 2 | 2 | 3 | 2 | 3 | 3 | 3 | 2 | 3 | 3 |
| 110759 | 5 | 3 | 3 | 3 | 3 | 3 | 3 | 3 | 3 | 3 | 3 | 3 | 3 | 3 | 3 | 4 | 3 | 3 | 3 | 3 | 3 | 3 | 3 | 3 | 3 | 3 | 3 | 3 | 3 | 3 | 3 | 4 | 3 |
| 110762 | 1 | 2 | 2 | 2 | 2 | 2 | 2 | 2 | 2 | 2 | 3 | 3 | 2 | 2 | 2 | 2 | 3 | 3 | 3 | 3 | 2 | 2 | 2 | 2 | 3 | 3 | 3 | 2 | 3 | 3 | 3 | 3 | 3 |
| 110762 | 2 | 3 | 3 | 2 | 3 | 3 | 3 | 3 | 3 | 3 | 3 | 3 | 3 | 3 | 3 | 3 | 3 | 3 | 3 | 3 | 3 | 3 | 3 | 3 | 3 | 3 | 3 | 3 | 3 | 4 | 3 | 3 | 3 |
| 110762 | 3 | 2 | 2 | 2 | 2 | 2 | 2 | 2 | 2 | 2 | 3 | 3 | 2 | 2 | 2 | 2 | 3 | 3 | 3 | 3 | 2 | 2 | 2 | 2 | 3 | 3 | 3 | 2 | 3 | 3 | 3 | 3 | 3 |
| 110762 | 4 | 2 | 2 | 2 | 2 | 2 | 2 | 2 | 2 | 2 | 2 | 2 | 2 | 2 | 2 | 2 | 2 | 2 | 2 | 2 | 2 | 2 | 2 | 2 | 2 | 2 | 2 | 2 | 3 | 3 | 3 | 3 | 3 |
| 110762 | 5 | 2 | 2 | 2 | 2 | 2 | 2 | 2 | 2 | 2 | 3 | 3 | 2 | 2 | 2 | 2 | 3 | 3 | 3 | 3 | 2 | 2 | 2 | 2 | 3 | 3 | 3 | 2 | 3 | 3 | 3 | 3 | 3 |
| 110771 | 1 | 1 | 2 | 2 | 2 | 2 | 2 | 2 | 2 | 2 | 2 | 2 | 2 | 2 | 2 | 2 | 2 | 2 | 2 | 2 | 2 | 2 | 2 | 2 | 2 | 2 | 2 | 2 | 2 | 2 | 2 | 2 | 2 |
| 110771 | 2 | 2 | 2 | 2 | 2 | 2 | 2 | 2 | 2 | 2 | 2 | 2 | 2 | 2 | 2 | 2 | 2 | 2 | 2 | 2 | 2 | 2 | 2 | 2 | 2 | 2 | 2 | 2 | 3 | 3 | 3 | 3 | 3 |
| 110771 | 3 | 1 | 2 | 2 | 2 | 2 | 2 | 2 | 2 | 2 | 2 | 2 | 2 | 2 | 2 | 2 | 2 | 2 | 2 | 2 | 2 | 1 | 2 | 2 | 2 | 2 | 2 | 2 | 3 | 3 | 3 | 3 | 3 |
| 110771 | 4 | 2 | 2 | 2 | 2 | 2 | 2 | 2 | 2 | 2 | 2 | 2 | 2 | 2 | 2 | 2 | 2 | 2 | 2 | 2 | 2 | 2 | 2 | 2 | 2 | 2 | 2 | 2 | 3 | 3 | 3 | 3 | 3 |
| 110771 | 5 | 2 | 2 | 2 | 2 | 2 | 2 | 2 | 2 | 2 | 2 | 2 | 2 | 2 | 2 | 2 | 2 | 2 | 2 | 2 | 2 | 2 | 2 | 2 | 2 | 2 | 2 | 2 | 3 | 3 | 3 | 3 | 3 |
| 110772 | 1 | 2 | 3 | 1 | 3 | 2 | 3 | 2 | 3 | 3 | 3 | 3 | 3 | 3 | 3 | 3 | 3 | 3 | 3 | 0 | 3 | 3 | 2 | 3 | 3 | 3 | 3 | 3 | 3 | 3 | 3 | 3 | 3 |
| 110772 | 2 | 3 | 4 | 2 | 3 | 3 | 3 | 3 | 3 | 3 | 3 | 3 | 3 | 3 | 3 | 3 | 3 | 3 | 3 | 3 | 3 | 3 | 3 | 3 | 3 | 3 | 3 | 3 | 3 | 3 | 3 | 3 | 3 |
| 110772 | 3 | 2 | 2 | 2 | 2 | 2 | 3 | 2 | 2 | 2 | 2 | 3 | 2 | 2 | 2 | 3 | 2 | 2 | 2 | 2 | 2 | 2 | 2 | 3 | 3 | 3 | 3 | 3 | 3 | 3 | 3 | 3 | 3 |
| 110772 | 4 | 0 | 1 | 0 | 1 | 1 | 0 | 2 | 0 | 1 | 1 | 1 | 0 | 1 | 0 | 0 | 0 | 2 | 1 | 1 | 0 | 0 | 1 | 0 | 0 | 1 | 1 | 0 | 3 | 3 | 3 | 3 | 3 |
| 110772 | 5 | 0 | 1 | 0 | 1 | 1 | 0 | 2 | 0 | 1 | 2 | 1 | 0 | 2 | 0 | 1 | 0 | 2 | 1 | 1 | 1 | 0 | 1 | 0 | 0 | 1 | 1 | 0 | 3 | 3 | 3 | 3 | 3 |
| 110823 | 1 | 4 | 4 | 4 | 4 | 4 | 4 | 4 | 3 | 4 | 4 | 4 | 4 | 4 | 4 | 3 | 4 | 4 | 4 | 3 | 4 | 3 | 4 | 4 | 4 | 4 | 4 | 4 | 4 | 4 | 4 | 4 | 4 |
| 110823 | 2 | 4 | 4 | 3 | 4 | 4 | 4 | 4 | 4 | 4 | 4 | 4 | 4 | 4 | 3 | 3 | 4 | 4 | 4 | 3 | 4 | 3 | 4 | 4 | 4 | 4 | 4 | 4 | 4 | 4 | 4 | 4 | 4 |
| 110823 | 3 | 4 | 4 | 3 | 4 | 4 | 4 | 4 | 3 | 4 | 4 | 4 | 4 | 4 | 4 | 4 | 4 | 4 | 4 | 3 | 4 | 3 | 4 | 4 | 4 | 4 | 4 | 4 | 4 | 4 | 3 | 4 | 4 |
| 110823 | 4 | 3 | 4 | 3 | 3 | 4 | 4 | 4 | 3 | 3 | 3 | 3 | 4 | 4 | 4 | 3 | 4 | 4 | 3 | 3 | 3 | 3 | 3 | 4 | 3 | 4 | 4 | 3 | 3 | 4 | 3 | 3 | 3 |
| 110823 | 5 | 4 | 4 | 3 | 4 | 4 | 4 | 4 | 3 | 3 | 3 | 3 | 4 | 4 | 3 | 3 | 4 | 3 | 3 | 3 | 4 | 3 | 4 | 3 | 3 | 4 | 4 | 3 | 3 | 3 | 3 | 3 | 3 |
| 115784 | 1 | 2 | 3 | 1 | 2 | 3 | 3 | 2 | 3 | 3 | 2 | 2 | 1 | 3 | 3 | 3 | 2 | 3 | 3 | 3 | 3 | 3 | 2 | 3 | 3 | 2 | 2 | 3 | 3 | 3 | 2 | 3 | 3 |
| 115784 | 2 | 1 | 1 | 0 | 0 | 1 | 1 | 0 | 1 | 2 | 1 | 1 | 1 | 0 | 1 | 1 | 3 | 1 | 2 | 1 | 1 | 1 | 1 | 1 | 1 | 1 | 1 | 1 | 3 | 3 | 2 | 3 | 3 |
| 115784 | 3 | 3 | 3 | 0 | 3 | 3 | 3 | 3 | 3 | 3 | 2 | 2 | 3 | 3 | 3 | 3 | 3 | 3 | 3 | 3 | 3 | 3 | 3 | 3 | 3 | 2 | 3 | 3 | 3 | 3 | 2 | 3 | 3 |
| 115784 | 4 | 3 | 3 | 1 | 3 | 3 | 3 | 3 | 3 | 3 | 2 | 2 | 3 | 3 | 3 | 3 | 3 | 3 | 3 | 3 | 3 | 3 | 3 | 3 | 3 | 2 | 3 | 3 | 3 | 3 | 2 | 3 | 3 |
| 115784 | 5 | 2 | 3 | 2 | 3 | 3 | 2 | 3 | 2 | 2 | 2 | 2 | 3 | 3 | 2 | 3 | 3 | 3 | 2 | 3 | 3 | 2 | 2 | 2 | 2 | 2 | 2 | 2 | 3 | 3 | 2 | 3 | 3 |
| 115785 | 1 | 2 | 2 | 1 | 3 | 2 | 1 | 2 | 1 | 2 | 2 | 2 | 2 | 1 | 2 | 1 | 2 | 2 | 2 | 2 | 3 | 2 | 2 | 2 | 2 | 2 | 2 | 2 | 2 | 3 | 2 | 2 | 2 |
| 115785 | 2 | 2 | 2 | 1 | 2 | 2 | 1 | 2 | 1 | 2 | 2 | 2 | 2 | 1 | 2 | 2 | 2 | 2 | 2 | 2 | 2 | 2 | 2 | 2 | 2 | 2 | 2 | 2 | 3 | 3 | 3 | 3 | 3 |
| 115785 | 3 | 2 | 2 | 1 | 3 | 2 | 1 | 2 | 1 | 2 | 2 | 2 | 1 | 1 | 2 | 2 | 2 | 2 | 2 | 2 | 3 | 2 | 2 | 2 | 2 | 2 | 2 | 2 | 3 | 3 | 3 | 3 | 3 |
| 115785 | 4 | 2 | 2 | 2 | 2 | 2 | 2 | 2 | 2 | 2 | 2 | 2 | 2 | 2 | 2 | 2 | 2 | 2 | 2 | 2 | 2 | 2 | 2 | 2 | 2 | 2 | 2 | 2 | 3 | 3 | 3 | 3 | 3 |
| 115785 | 5 | 2 | 2 | 2 | 3 | 2 | 2 | 2 | 1 | 2 | 2 | 2 | 2 | 2 | 2 | 2 | 2 | 2 | 2 | 2 | 3 | 2 | 2 | 2 | 2 | 2 | 2 | 2 | 3 | 4 | 3 | 3 | 3 |
| 115795 | 1 | 2 | 2 | 1 | 2 | 2 | 2 | 2 | 2 | 2 | 2 | 2 | 2 | 2 | 2 | 2 | 2 | 2 | 2 | 2 | 2 | 2 | 2 | 2 | 2 | 2 | 2 | 2 | 2 | 3 | 2 | 2 | 2 |
| 115795 | 2 | 2 | 2 | 0 | 2 | 2 | 2 | 2 | 2 | 2 | 2 | 2 | 2 | 2 | 2 | 2 | 2 | 2 | 2 | 2 | 2 | 2 | 2 | 2 | 2 | 2 | 2 | 2 | 2 | 3 | 2 | 2 | 2 |
| 115795 | 3 | 2 | 3 | 3 | 3 | 2 | 2 | 2 | 3 | 2 | 2 | 2 | 2 | 3 | 2 | 2 | 2 | 2 | 3 | 3 | 3 | 2 | 2 | 2 | 2 | 2 | 3 | 2 | 3 | 3 | 2 | 3 | 3 |
| 115795 | 4 | 2 | 2 | 0 | 2 | 2 | 2 | 2 | 2 | 2 | 2 | 2 | 2 | 2 | 2 | 2 | 2 | 2 | 2 | 2 | 2 | 2 | 2 | 2 | 2 | 2 | 2 | 2 | 2 | 3 | 2 | 2 | 2 |
| 115795 | 5 | 2 | 2 | 0 | 2 | 2 | 2 | 2 | 2 | 2 | 2 | 2 | 2 | 2 | 2 | 2 | 2 | 2 | 2 | 2 | 2 | 2 | 2 | 2 | 2 | 2 | 2 | 2 | 2 | 3 | 2 | 2 | 2 |
| 116097 | 1 | 0 | 0 | 2 | 2 | 2 | 2 | 1 | 2 | 2 | 2 | 2 | 0 | 2 | 3 | 3 | 2 | 2 | 0 | 2 | 2 | 2 | 2 | 2 | 0 | 2 | 2 | 2 | 3 | 3 | 2 | 2 | 2 |
| 116097 | 2 | 2 | 2 | 2 | 2 | 2 | 2 | 2 | 2 | 2 | 2 | 2 | 1 | 2 | 3 | 3 | 2 | 2 | 2 | 2 | 2 | 2 | 2 | 2 | 2 | 2 | 2 | 2 | 3 | 3 | 2 | 2 | 2 |
| 116097 | 3 | 2 | 2 | 2 | 2 | 2 | 2 | 2 | 2 | 2 | 2 | 2 | 0 | 2 | 3 | 3 | 2 | 2 | 2 | 2 | 2 | 2 | 2 | 2 | 2 | 2 | 2 | 2 | 3 | 3 | 2 | 2 | 2 |
| 116097 | 4 | 2 | 2 | 2 | 2 | 2 | 2 | 2 | 2 | 2 | 2 | 2 | 1 | 2 | 3 | 3 | 2 | 2 | 2 | 2 | 2 | 2 | 2 | 2 | 2 | 2 | 2 | 2 | 3 | 3 | 2 | 2 | 2 |
| 116097 | 5 | 2 | 2 | 2 | 2 | 2 | 2 | 2 | 2 | 2 | 2 | 2 | 1 | 1 | 3 | 3 | 2 | 2 | 2 | 2 | 2 | 2 | 2 | 2 | 2 | 2 | 2 | 2 | 3 | 3 | 2 | 2 | 2 |
| 116103 | 1 | 3 | 3 | 0 | 2 | 3 | 3 | 3 | 3 | 3 | 3 | 3 | 3 | 3 | 3 | 3 | 3 | 3 | 3 | 3 | 3 | 3 | 3 | 3 | 3 | 3 | 3 | 3 | 2 | 3 | 2 | 2 | 3 |
| 116103 | 2 | 3 | 3 | 1 | 2 | 3 | 3 | 3 | 3 | 3 | 3 | 3 | 3 | 3 | 3 | 3 | 3 | 3 | 3 | 3 | 3 | 3 | 3 | 3 | 3 | 3 | 3 | 3 | 2 | 3 | 2 | 2 | 3 |
| 116103 | 3 | 3 | 3 | 0 | 2 | 3 | 3 | 3 | 3 | 3 | 3 | 3 | 3 | 3 | 3 | 3 | 3 | 3 | 3 | 3 | 3 | 3 | 3 | 3 | 3 | 3 | 3 | 3 | 2 | 3 | 2 | 2 | 3 |
| 116103 | 4 | 3 | 3 | 1 | 2 | 3 | 3 | 3 | 3 | 3 | 3 | 3 | 3 | 3 | 3 | 3 | 3 | 3 | 3 | 3 | 3 | 3 | 3 | 3 | 3 | 3 | 3 | 3 | 2 | 3 | 2 | 2 | 3 |
| 116103 | 5 | 3 | 3 | 1 | 2 | 3 | 3 | 3 | 3 | 3 | 3 | 3 | 3 | 3 | 3 | 3 | 3 | 3 | 3 | 3 | 3 | 3 | 3 | 3 | 3 | 3 | 3 | 3 | 2 | 3 | 2 | 2 | 3 |
| 116109 | 1 | 3 | 1 | 2 | 1 | 4 | 3 | 1 | 3 | 2 | 2 | 4 | 3 | 2 | 2 | 3 | 3 | 3 | 3 | 3 | 3 | 3 | 3 | 3 | 4 | 3 | 2 | 2 | 2 | 3 | 2 | 3 | 2 |
| 116109 | 2 | 4 | 3 | 1 | 1 | 2 | 1 | 2 | 3 | 2 | 3 | 3 | 3 | 2 | 3 | 3 | 2 | 3 | 2 | 3 | 3 | 3 | 3 | 3 | 3 | 2 | 3 | 3 | 2 | 3 | 2 | 3 | 3 |
| 116109 | 3 | 2 | 1 | 1 | 2 | 1 | 2 | 2 | 2 | 2 | 0 | 2 | 3 | 2 | 1 | 2 | 1 | 2 | 2 | 0 | 2 | 3 | 0 | 3 | 3 | 2 | 2 | 2 | 3 | 3 | 3 | 3 | 2 |
| 116109 | 4 | 0 | 1 | 1 | 1 | 2 | 2 | 1 | 1 | 2 | 1 | 2 | 3 | 2 | 1 | 2 | 2 | 2 | 2 | 2 | 2 | 3 | 2 | 3 | 3 | 2 | 2 | 2 | 3 | 3 | 3 | 3 | 2 |
| 116109 | 5 | 3 | 2 | 2 | 1 | 1 | 2 | 1 | 3 | 2 | 1 | 3 | 3 | 2 | 2 | 3 | 2 | 2 | 3 | 3 | 3 | 3 | 2 | 3 | 2 | 3 | 2 | 2 | 3 | 3 | 2 | 3 | 3 |
| 116110 | 1 | 3 | 2 | 0 | 3 | 2 | 3 | 2 | 3 | 2 | 2 | 3 | 2 | 2 | 2 | 3 | 3 | 2 | 2 | 3 | 3 | 3 | 2 | 3 | 3 | 3 | 3 | 2 | 3 | 3 | 2 | 3 | 3 |
| 116110 | 2 | 3 | 1 | 1 | 3 | 2 | 3 | 2 | 3 | 2 | 2 | 3 | 2 | 1 | 2 | 3 | 3 | 2 | 0 | 3 | 3 | 3 | 2 | 3 | 3 | 3 | 3 | 2 | 3 | 3 | 2 | 3 | 3 |
| 116110 | 3 | 3 | 1 | 0 | 3 | 2 | 3 | 2 | 3 | 2 | 2 | 3 | 0 | 0 | 2 | 3 | 3 | 2 | 0 | 3 | 3 | 3 | 2 | 3 | 3 | 3 | 3 | 2 | 3 | 3 | 2 | 3 | 3 |
| 116110 | 4 | 3 | 2 | 0 | 3 | 2 | 3 | 2 | 3 | 2 | 2 | 4 | 2 | 1 | 2 | 3 | 3 | 2 | 0 | 3 | 3 | 3 | 2 | 3 | 3 | 3 | 3 | 2 | 3 | 4 | 0 | 3 | 3 |
| 116110 | 5 | 4 | 2 | 0 | 3 | 2 | 3 | 2 | 3 | 2 | 2 | 3 | 1 | 1 | 2 | 3 | 3 | 2 | 0 | 3 | 3 | 3 | 2 | 3 | 3 | 3 | 3 | 2 | 3 | 3 | 0 | 3 | 3 |
| 116113 | 1 | 2 | 2 | 1 | 1 | 2 | 2 | 2 | 1 | 2 | 2 | 2 | 3 | 1 | 3 | 2 | 2 | 2 | 2 | 2 | 2 | 2 | 1 | 2 | 2 | 2 | 2 | 3 | 3 | 3 | 3 | 2 | 2 |
| 116113 | 2 | 2 | 2 | 2 | 2 | 2 | 2 | 2 | 2 | 2 | 2 | 2 | 3 | 2 | 3 | 2 | 2 | 2 | 2 | 2 | 2 | 2 | 2 | 2 | 2 | 2 | 2 | 3 | 3 | 3 | 3 | 2 | 2 |
| 116113 | 3 | 2 | 1 | 1 | 1 | 2 | 2 | 2 | 2 | 2 | 2 | 1 | 3 | 2 | 3 | 2 | 2 | 2 | 2 | 2 | 2 | 2 | 2 | 2 | 2 | 2 | 2 | 3 | 3 | 3 | 3 | 2 | 2 |
| 116113 | 4 | 3 | 1 | 1 | 1 | 2 | 2 | 2 | 2 | 2 | 2 | 1 | 3 | 1 | 3 | 2 | 2 | 2 | 2 | 2 | 2 | 2 | 1 | 2 | 2 | 2 | 2 | 3 | 3 | 2 | 2 | 2 | 2 |
| 116113 | 5 | 2 | 1 | 1 | 1 | 1 | 2 | 2 | 2 | 2 | 1 | 2 | 3 | 2 | 3 | 2 | 1 | 2 | 2 | 2 | 2 | 2 | 2 | 2 | 2 | 2 | 2 | 3 | 3 | 3 | 3 | 2 | 2 |
| 116115 | 1 | 0 | 0 | 0 | 0 | 0 | 2 | 0 | 2 | 2 | 0 | 3 | 2 | 0 | 3 | 3 | 2 | 1 | 0 | 0 | 3 | 3 | 0 | 2 | 3 | 3 | 3 | 3 | 3 | 3 | 2 | 3 | 2 |
| 116115 | 2 | 0 | 0 | 0 | 0 | 0 | 0 | 0 | 0 | 2 | 0 | 3 | 2 | 2 | 3 | 3 | 2 | 0 | 2 | 0 | 3 | 3 | 0 | 2 | 3 | 4 | 3 | 3 | 3 | 3 | 2 | 3 | 2 |
| 116115 | 3 | 0 | 0 | 0 | 0 | 0 | 4 | 0 | 3 | 3 | 0 | 3 | 2 | 0 | 3 | 3 | 2 | 1 | 2 | 0 | 3 | 3 | 0 | 2 | 3 | 3 | 3 | 3 | 3 | 3 | 2 | 3 | 2 |
| 116115 | 4 | 0 | 0 | 0 | 0 | 0 | 2 | 0 | 2 | 2 | 0 | 3 | 2 | 0 | 3 | 3 | 2 | 0 | 0 | 0 | 3 | 3 | 0 | 2 | 3 | 3 | 3 | 3 | 3 | 3 | 1 | 3 | 2 |
| 116115 | 5 | 2 | 0 | 1 | 0 | 2 | 2 | 0 | 2 | 2 | 1 | 2 | 2 | 2 | 2 | 2 | 2 | 1 | 2 | 1 | 2 | 2 | 1 | 2 | 2 | 2 | 2 | 2 | 3 | 3 | 2 | 3 | 2 |
| 116117 | 1 | 2 | 2 | 1 | 2 | 2 | 3 | 2 | 3 | 2 | 2 | 3 | 2 | 0 | 3 | 3 | 2 | 3 | 2 | 3 | 3 | 3 | 4 | 3 | 2 | 3 | 3 | 2 | 2 | 3 | 2 | 2 | 2 |
| 116117 | 2 | 2 | 2 | 1 | 1 | 1 | 2 | 2 | 2 | 2 | 2 | 0 | 2 | 1 | 1 | 3 | 2 | 1 | 2 | 2 | 1 | 1 | 0 | 1 | 0 | 3 | 3 | 2 | 2 | 3 | 2 | 3 | 3 |
| 116117 | 3 | 2 | 2 | 1 | 0 | 2 | 2 | 2 | 2 | 1 | 2 | 1 | 2 | 2 | 2 | 3 | 2 | 2 | 1 | 2 | 2 | 1 | 2 | 2 | 1 | 3 | 3 | 2 | 2 | 3 | 1 | 3 | 3 |
| 116117 | 4 | 2 | 2 | 1 | 0 | 2 | 0 | 2 | 1 | 0 | 2 | 1 | 2 | 2 | 2 | 3 | 0 | 2 | 2 | 2 | 2 | 2 | 2 | 2 | 1 | 3 | 3 | 2 | 2 | 3 | 2 | 3 | 3 |
| 116117 | 5 | 3 | 4 | 3 | 2 | 1 | 3 | 3 | 3 | 3 | 3 | 0 | 1 | 3 | 1 | 3 | 3 | 2 | 0 | 3 | 1 | 0 | 1 | 0 | 0 | 3 | 4 | 3 | 3 | 3 | 3 | 3 | 3 |
| 116123 | 1 | 2 | 2 | 2 | 1 | 2 | 2 | 2 | 2 | 2 | 1 | 2 | 2 | 2 | 2 | 2 | 2 | 2 | 2 | 2 | 2 | 2 | 2 | 2 | 2 | 2 | 2 | 2 | 2 | 2 | 2 | 2 | 2 |
| 116123 | 2 | 2 | 4 | 0 | 0 | 3 | 4 | 3 | 3 | 3 | 3 | 2 | 3 | 2 | 3 | 3 | 3 | 3 | 3 | 2 | 3 | 3 | 4 | 3 | 3 | 2 | 3 | 2 | 3 | 3 | 3 | 0 | 0 |
| 116123 | 3 | 2 | 4 | 2 | 4 | 3 | 3 | 2 | 3 | 2 | 3 | 1 | 2 | 3 | 2 | 3 | 3 | 3 | 2 | 1 | 3 | 3 | 3 | 2 | 2 | 3 | 3 | 3 | 2 | 3 | 3 | 3 | 2 |
| 116123 | 4 | 2 | 2 | 1 | 2 | 1 | 3 | 2 | 2 | 2 | 2 | 3 | 2 | 2 | 3 | 3 | 3 | 2 | 3 | 2 | 2 | 2 | 2 | 3 | 3 | 2 | 2 | 3 | 3 | 2 | 2 | 3 | 2 |
| 116131 | 1 | 2 | 4 | 3 | 4 | 3 | 3 | 3 | 3 | 3 | 3 | 3 | 3 | 3 | 3 | 3 | 3 | 3 | 3 | 3 | 3 | 3 | 3 | 3 | 1 | 3 | 3 | 3 | 3 | 3 | 3 | 3 | 3 |
| 116131 | 2 | 2 | 4 | 1 | 3 | 4 | 3 | 3 | 3 | 3 | 3 | 3 | 3 | 3 | 3 | 3 | 3 | 4 | 3 | 3 | 3 | 3 | 4 | 3 | 3 | 3 | 3 | 3 | 3 | 3 | 3 | 3 | 3 |
| 116131 | 3 | 3 | 3 | 3 | 3 | 3 | 3 | 4 | 3 | 2 | 3 | 3 | 3 | 3 | 0 | 2 | 3 | 3 | 3 | 3 | 3 | 3 | 3 | 3 | 3 | 3 | 3 | 3 | 3 | 3 | 2 | 3 | 2 |
| 116131 | 4 | 4 | 4 | 2 | 4 | 4 | 3 | 4 | 3 | 2 | 3 | 3 | 4 | 3 | 3 | 3 | 3 | 4 | 3 | 3 | 3 | 3 | 3 | 4 | 3 | 4 | 3 | 3 | 3 | 4 | 3 | 3 | 3 |
| 116131 | 5 | 3 | 4 | 0 | 3 | 3 | 3 | 3 | 3 | 3 | 3 | 3 | 3 | 3 | 3 | 3 | 3 | 2 | 3 | 4 | 3 | 3 | 3 | 3 | 3 | 3 | 3 | 3 | 3 | 3 | 3 | 3 | 3 |
| 117888 | 1 | 2 | 2 | 2 | 2 | 3 | 3 | 2 | 3 | 2 | 2 | 2 | 3 | 2 | 3 | 3 | 2 | 3 | 3 | 2 | 3 | 3 | 4 | 3 | 3 | 3 | 2 | 2 | 2 | 3 | 3 | 3 | 2 |
| 117888 | 2 | 3 | 4 | 3 | 4 | 3 | 3 | 4 | 3 | 4 | 3 | 4 | 3 | 3 | 3 | 3 | 3 | 3 | 3 | 4 | 3 | 4 | 3 | 3 | 3 | 3 | 3 | 3 | 3 | 3 | 3 | 3 | 3 |
| 117888 | 3 | 3 | 4 | 3 | 3 | 3 | 3 | 3 | 3 | 3 | 3 | 3 | 3 | 3 | 3 | 3 | 3 | 3 | 3 | 4 | 3 | 4 | 3 | 4 | 3 | 3 | 3 | 3 | 3 | 3 | 3 | 3 | 3 |
| 117888 | 4 | 1 | 2 | 2 | 2 | 2 | 2 | 1 | 2 | 1 | 2 | 2 | 0 | 2 | 2 | 2 | 0 | 2 | 2 | 2 | 2 | 1 | 2 | 2 | 2 | 1 | 0 | 2 | 2 | 2 | 2 | 2 | 2 |
| 117888 | 5 | 3 | 3 | 3 | 4 | 3 | 3 | 3 | 3 | 3 | 3 | 4 | 4 | 3 | 3 | 3 | 3 | 3 | 3 | 3 | 3 | 3 | 4 | 4 | 3 | 3 | 3 | 4 | 3 | 3 | 3 | 3 | 3 |
| 117892 | 1 | 3 | 3 | 3 | 3 | 3 | 3 | 3 | 3 | 2 | 2 | 2 | 2 | 2 | 2 | 3 | 2 | 3 | 3 | 3 | 3 | 3 | 3 | 3 | 2 | 3 | 3 | 2 | 3 | 3 | 3 | 2 | 3 |
| 117892 | 2 | 3 | 3 | 0 | 3 | 3 | 2 | 3 | 2 | 2 | 3 | 3 | 2 | 3 | 2 | 3 | 3 | 3 | 3 | 3 | 3 | 3 | 3 | 3 | 2 | 2 | 3 | 3 | 3 | 3 | 2 | 2 | 3 |
| 117892 | 3 | 0 | 1 | 1 | 1 | 1 | 1 | 2 | 0 | 0 | 2 | 2 | 2 | 1 | 1 | 0 | 1 | 1 | 1 | 2 | 1 | 2 | 1 | 2 | 0 | 2 | 0 | 0 | 3 | 3 | 3 | 3 | 3 |
| 117892 | 4 | 1 | 1 | 1 | 0 | 2 | 1 | 1 | 1 | 1 | 2 | 0 | 1 | 0 | 1 | 1 | 1 | 1 | 1 | 1 | 0 | 1 | 0 | 2 | 1 | 0 | 1 | 1 | 2 | 2 | 2 | 2 | 2 |
| 117892 | 5 | 2 | 2 | 2 | 2 | 3 | 2 | 2 | 2 | 2 | 2 | 2 | 2 | 2 | 2 | 2 | 3 | 2 | 2 | 3 | 3 | 2 | 3 | 2 | 2 | 2 | 2 | 2 | 2 | 2 | 2 | 3 | 2 |
| 119406 | 1 | 2 | 3 | 0 | 3 | 3 | 2 | 3 | 2 | 2 | 3 | 2 | 2 | 2 | 2 | 3 | 2 | 3 | 2 | 3 | 2 | 3 | 3 | 2 | 2 | 2 | 2 | 3 | 3 | 3 | 2 | 2 | 2 |
| 119406 | 2 | 2 | 3 | 1 | 3 | 3 | 2 | 3 | 2 | 2 | 3 | 2 | 2 | 2 | 2 | 3 | 2 | 3 | 2 | 3 | 2 | 3 | 3 | 2 | 2 | 2 | 2 | 3 | 3 | 3 | 2 | 2 | 2 |
| 119406 | 3 | 2 | 4 | 1 | 3 | 3 | 2 | 3 | 2 | 2 | 3 | 2 | 2 | 2 | 2 | 3 | 2 | 3 | 2 | 3 | 2 | 3 | 3 | 2 | 2 | 2 | 2 | 3 | 3 | 3 | 2 | 2 | 2 |
| 119406 | 4 | 2 | 3 | 0 | 3 | 3 | 2 | 3 | 2 | 2 | 3 | 2 | 2 | 2 | 2 | 3 | 2 | 3 | 2 | 3 | 2 | 3 | 3 | 2 | 2 | 2 | 2 | 3 | 3 | 3 | 2 | 2 | 2 |
| 119406 | 5 | 2 | 3 | 0 | 3 | 3 | 1 | 3 | 2 | 2 | 3 | 2 | 2 | 2 | 2 | 3 | 2 | 3 | 2 | 3 | 2 | 3 | 3 | 2 | 2 | 2 | 2 | 3 | 3 | 3 | 2 | 2 | 2 |
| 119407 | 1 | 3 | 3 | 2 | 4 | 3 | 3 | 3 | 3 | 3 | 2 | 2 | 3 | 3 | 3 | 3 | 3 | 3 | 3 | 3 | 3 | 3 | 3 | 4 | 3 | 3 | 3 | 3 | 3 | 3 | 3 | 3 | 3 |
| 119407 | 2 | 3 | 3 | 2 | 3 | 3 | 3 | 3 | 3 | 3 | 2 | 2 | 3 | 3 | 3 | 3 | 3 | 3 | 3 | 3 | 3 | 3 | 3 | 3 | 3 | 3 | 3 | 3 | 3 | 3 | 3 | 3 | 3 |
| 119407 | 3 | 3 | 3 | 2 | 3 | 3 | 3 | 3 | 3 | 3 | 2 | 2 | 3 | 3 | 3 | 3 | 3 | 3 | 3 | 3 | 3 | 3 | 3 | 3 | 3 | 3 | 3 | 3 | 3 | 3 | 3 | 3 | 3 |
| 119407 | 4 | 3 | 3 | 2 | 3 | 3 | 3 | 3 | 3 | 3 | 2 | 2 | 3 | 3 | 3 | 3 | 3 | 3 | 3 | 3 | 3 | 3 | 3 | 3 | 3 | 3 | 3 | 3 | 3 | 3 | 3 | 3 | 3 |
| 119407 | 5 | 3 | 3 | 2 | 3 | 3 | 3 | 3 | 3 | 3 | 2 | 2 | 3 | 3 | 3 | 3 | 3 | 3 | 3 | 3 | 3 | 3 | 3 | 3 | 3 | 3 | 3 | 3 | 3 | 3 | 3 | 3 | 3 |
| 119413 | 1 | 4 | 4 | 1 | 4 | 2 | 4 | 4 | 4 | 4 | 4 | 4 | 4 | 4 | 4 | 4 | 4 | 4 | 4 | 4 | 4 | 4 | 4 | 4 | 4 | 4 | 4 | 3 | 3 | 4 | 3 | 3 | 3 |
| 119413 | 2 | 3 | 4 | 0 | 4 | 2 | 4 | 3 | 4 | 4 | 4 | 4 | 4 | 4 | 4 | 4 | 4 | 4 | 4 | 4 | 4 | 4 | 4 | 4 | 4 | 4 | 4 | 4 | 3 | 4 | 3 | 3 | 3 |
| 119413 | 3 | 4 | 4 | 1 | 4 | 2 | 4 | 3 | 4 | 4 | 4 | 4 | 4 | 4 | 4 | 4 | 4 | 4 | 4 | 4 | 4 | 4 | 4 | 4 | 4 | 4 | 4 | 4 | 3 | 4 | 3 | 3 | 3 |
| 119413 | 4 | 4 | 4 | 1 | 4 | 2 | 2 | 4 | 2 | 2 | 3 | 2 | 2 | 2 | 2 | 2 | 2 | 3 | 2 | 3 | 2 | 2 | 4 | 2 | 2 | 2 | 2 | 2 | 3 | 4 | 3 | 3 | 3 |
| 119413 | 5 | 3 | 3 | 0 | 3 | 3 | 3 | 4 | 3 | 2 | 4 | 3 | 3 | 2 | 3 | 3 | 3 | 3 | 2 | 3 | 3 | 3 | 3 | 3 | 3 | 3 | 3 | 3 | 3 | 4 | 3 | 3 | 3 |
| 119447 | 1 | 2 | 2 | 3 | 3 | 3 | 3 | 3 | 3 | 2 | 3 | 3 | 3 | 3 | 3 | 3 | 3 | 3 | 3 | 3 | 3 | 3 | 3 | 3 | 3 | 3 | 2 | 3 | 3 | 3 | 2 | 3 | 3 |
| 119447 | 2 | 2 | 2 | 3 | 3 | 3 | 3 | 3 | 3 | 2 | 3 | 3 | 3 | 3 | 3 | 3 | 3 | 3 | 3 | 3 | 3 | 3 | 3 | 3 | 3 | 3 | 2 | 3 | 3 | 3 | 2 | 3 | 3 |
| 119447 | 3 | 2 | 2 | 3 | 3 | 3 | 3 | 3 | 3 | 2 | 3 | 3 | 3 | 3 | 3 | 3 | 3 | 3 | 3 | 3 | 3 | 3 | 3 | 3 | 3 | 3 | 2 | 3 | 3 | 3 | 2 | 3 | 3 |
| 119447 | 4 | 2 | 2 | 0 | 3 | 1 | 2 | 1 | 3 | 3 | 3 | 3 | 3 | 3 | 3 | 3 | 2 | 2 | 3 | 3 | 3 | 3 | 3 | 3 | 2 | 3 | 2 | 2 | 2 | 3 | 1 | 2 | 3 |
| 119447 | 5 | 2 | 2 | 3 | 3 | 3 | 3 | 3 | 3 | 2 | 3 | 3 | 3 | 3 | 3 | 3 | 3 | 3 | 3 | 3 | 3 | 3 | 3 | 3 | 3 | 3 | 2 | 3 | 3 | 3 | 2 | 3 | 3 |
| 120790 | 1 | 3 | 4 | 3 | 4 | 3 | 3 | 4 | 3 | 3 | 3 | 3 | 3 | 3 | 3 | 3 | 3 | 3 | 3 | 3 | 3 | 3 | 4 | 4 | 3 | 4 | 3 | 3 | 3 | 3 | 3 | 3 | 2 |
| 120790 | 2 | 2 | 2 | 3 | 2 | 0 | 2 | 1 | 2 | 2 | 2 | 2 | 2 | 2 | 2 | 2 | 2 | 1 | 1 | 2 | 2 | 2 | 2 | 2 | 3 | 2 | 2 | 2 | 2 | 3 | 1 | 2 | 1 |
| 120790 | 3 | 3 | 4 | 4 | 4 | 3 | 3 | 4 | 4 | 3 | 3 | 3 | 3 | 3 | 3 | 3 | 3 | 3 | 3 | 3 | 4 | 3 | 4 | 3 | 3 | 3 | 3 | 3 | 3 | 3 | 3 | 3 | 2 |
| 120790 | 4 | 2 | 2 | 3 | 2 | 1 | 2 | 2 | 2 | 2 | 2 | 2 | 2 | 2 | 2 | 2 | 2 | 2 | 1 | 2 | 1 | 2 | 2 | 2 | 3 | 2 | 2 | 2 | 2 | 3 | 2 | 2 | 2 |
| 120790 | 5 | 2 | 2 | 3 | 2 | 1 | 2 | 1 | 2 | 2 | 2 | 1 | 2 | 1 | 2 | 2 | 2 | 2 | 1 | 2 | 1 | 0 | 2 | 2 | 3 | 2 | 2 | 2 | 2 | 3 | 1 | 2 | 2 |
| 120792 | 1 | 2 | 2 | 1 | 2 | 1 | 2 | 1 | 1 | 2 | 2 | 2 | 3 | 2 | 2 | 1 | 2 | 1 | 3 | 2 | 1 | 2 | 2 | 2 | 3 | 2 | 2 | 2 | 2 | 3 | 2 | 2 | 2 |
| 120792 | 2 | 3 | 1 | 1 | 3 | 4 | 2 | 2 | 3 | 3 | 4 | 3 | 3 | 3 | 3 | 2 | 2 | 3 | 3 | 2 | 3 | 3 | 3 | 3 | 3 | 2 | 2 | 2 | 3 | 3 | 3 | 3 | 3 |
| 120792 | 3 | 4 | 2 | 2 | 4 | 3 | 2 | 2 | 3 | 3 | 4 | 3 | 3 | 3 | 3 | 2 | 2 | 3 | 3 | 2 | 4 | 4 | 4 | 3 | 4 | 2 | 2 | 2 | 3 | 4 | 3 | 3 | 3 |
| 120792 | 4 | 2 | 3 | 2 | 2 | 0 | 2 | 0 | 3 | 3 | 2 | 3 | 3 | 3 | 3 | 2 | 3 | 2 | 3 | 3 | 2 | 2 | 3 | 3 | 3 | 2 | 3 | 3 | 2 | 3 | 2 | 3 | 3 |
| 120792 | 5 | 2 | 2 | 1 | 2 | 3 | 3 | 3 | 3 | 2 | 3 | 3 | 3 | 3 | 3 | 2 | 3 | 1 | 3 | 3 | 3 | 2 | 3 | 3 | 3 | 2 | 2 | 3 | 3 | 3 | 3 | 2 | 3 |
| 120796 | 1 | 2 | 3 | 3 | 3 | 3 | 3 | 3 | 4 | 3 | 3 | 3 | 3 | 3 | 3 | 3 | 4 | 3 | 3 | 3 | 3 | 3 | 3 | 2 | 3 | 3 | 2 | 3 | 3 | 3 | 2 | 3 | 2 |
| 120796 | 2 | 2 | 2 | 3 | 3 | 2 | 3 | 2 | 3 | 3 | 3 | 3 | 3 | 3 | 3 | 3 | 3 | 2 | 3 | 3 | 3 | 3 | 3 | 3 | 3 | 3 | 3 | 2 | 3 | 3 | 3 | 3 | 3 |
| 120796 | 3 | 2 | 2 | 3 | 4 | 2 | 3 | 2 | 3 | 3 | 3 | 4 | 3 | 4 | 3 | 3 | 3 | 2 | 3 | 4 | 3 | 3 | 3 | 3 | 3 | 4 | 3 | 2 | 3 | 3 | 3 | 3 | 3 |
| 120796 | 4 | 2 | 2 | 3 | 3 | 0 | 3 | 2 | 3 | 3 | 3 | 3 | 3 | 4 | 3 | 3 | 3 | 2 | 3 | 3 | 4 | 3 | 3 | 3 | 3 | 3 | 3 | 2 | 3 | 3 | 3 | 3 | 3 |
| 120796 | 5 | 1 | 2 | 3 | 3 | 2 | 3 | 1 | 3 | 3 | 3 | 3 | 3 | 3 | 3 | 3 | 3 | 2 | 3 | 3 | 3 | 3 | 3 | 3 | 3 | 3 | 3 | 2 | 3 | 3 | 3 | 3 | 3 |
| 120797 | 1 | 2 | 2 | 2 | 2 | 0 | 2 | 2 | 2 | 2 | 2 | 3 | 3 | 3 | 2 | 2 | 2 | 2 | 3 | 2 | 0 | 0 | 3 | 3 | 2 | 2 | 2 | 2 | 3 | 3 | 3 | 3 | 3 |
| 120797 | 2 | 2 | 2 | 3 | 3 | 0 | 3 | 1 | 4 | 3 | 3 | 4 | 3 | 4 | 3 | 3 | 3 | 1 | 3 | 4 | 4 | 3 | 3 | 4 | 4 | 4 | 4 | 2 | 3 | 3 | 2 | 3 | 3 |
| 120797 | 3 | 2 | 2 | 4 | 3 | 0 | 3 | 2 | 3 | 3 | 3 | 3 | 3 | 4 | 3 | 3 | 3 | 2 | 3 | 4 | 3 | 3 | 3 | 3 | 3 | 3 | 3 | 2 | 3 | 4 | 2 | 3 | 3 |
| 120797 | 4 | 2 | 2 | 4 | 3 | 0 | 3 | 2 | 3 | 3 | 3 | 3 | 3 | 4 | 3 | 3 | 3 | 2 | 3 | 4 | 4 | 3 | 4 | 3 | 3 | 3 | 3 | 2 | 3 | 4 | 2 | 3 | 3 |
| 120797 | 5 | 2 | 2 | 0 | 0 | 0 | 2 | 0 | 2 | 2 | 0 | 3 | 3 | 4 | 1 | 1 | 1 | 0 | 3 | 1 | 2 | 2 | 3 | 3 | 2 | 1 | 2 | 2 | 3 | 3 | 3 | 3 | 3 |
| 120798 | 1 | 2 | 2 | 1 | 2 | 3 | 2 | 2 | 3 | 2 | 3 | 2 | 4 | 2 | 2 | 3 | 3 | 2 | 3 | 3 | 3 | 3 | 3 | 3 | 3 | 2 | 3 | 3 | 3 | 3 | 2 | 2 | 3 |
| 120798 | 2 | 2 | 2 | 1 | 2 | 3 | 2 | 0 | 3 | 2 | 3 | 2 | 4 | 2 | 2 | 3 | 3 | 2 | 3 | 3 | 3 | 3 | 3 | 3 | 3 | 2 | 3 | 3 | 3 | 3 | 2 | 2 | 3 |
| 120798 | 3 | 2 | 2 | 1 | 2 | 3 | 2 | 1 | 3 | 2 | 3 | 2 | 4 | 0 | 2 | 3 | 3 | 2 | 3 | 3 | 3 | 3 | 3 | 3 | 3 | 2 | 3 | 3 | 3 | 3 | 2 | 2 | 3 |
| 120798 | 4 | 2 | 2 | 1 | 1 | 3 | 2 | 2 | 3 | 2 | 3 | 2 | 3 | 2 | 2 | 3 | 3 | 2 | 3 | 3 | 3 | 3 | 3 | 3 | 3 | 1 | 3 | 3 | 3 | 3 | 2 | 2 | 3 |
| 120798 | 5 | 2 | 0 | 1 | 2 | 3 | 2 | 2 | 3 | 2 | 3 | 2 | 4 | 2 | 2 | 3 | 3 | 2 | 3 | 3 | 3 | 3 | 3 | 3 | 3 | 2 | 3 | 3 | 3 | 3 | 2 | 2 | 3 |
| 120799 | 1 | 2 | 1 | 0 | 2 | 0 | 2 | 2 | 1 | 0 | 2 | 2 | 2 | 2 | 2 | 2 | 2 | 2 | 2 | 2 | 2 | 2 | 2 | 3 | 2 | 1 | 2 | 2 | 2 | 3 | 2 | 2 | 3 |
| 120799 | 2 | 1 | 0 | 1 | 0 | 0 | 2 | 1 | 0 | 2 | 2 | 2 | 2 | 0 | 2 | 2 | 1 | 2 | 2 | 2 | 2 | 2 | 2 | 3 | 0 | 1 | 1 | 2 | 2 | 3 | 2 | 2 | 3 |
| 120799 | 3 | 1 | 0 | 0 | 1 | 0 | 2 | 2 | 2 | 2 | 2 | 2 | 2 | 1 | 1 | 2 | 2 | 0 | 2 | 2 | 2 | 2 | 2 | 3 | 2 | 2 | 2 | 2 | 2 | 3 | 2 | 2 | 3 |
| 120799 | 4 | 3 | 3 | 2 | 2 | 3 | 3 | 2 | 2 | 2 | 3 | 3 | 2 | 2 | 2 | 3 | 2 | 2 | 3 | 3 | 3 | 3 | 2 | 3 | 2 | 3 | 3 | 3 | 2 | 3 | 2 | 3 | 2 |
| 120799 | 5 | 1 | 0 | 0 | 1 | 0 | 2 | 2 | 1 | 2 | 0 | 2 | 1 | 2 | 1 | 2 | 0 | 2 | 2 | 1 | 2 | 2 | 2 | 3 | 2 | 1 | 2 | 0 | 2 | 3 | 2 | 2 | 3 |
| 123975 | 1 | 2 | 2 | 3 | 3 | 2 | 2 | 2 | 3 | 2 | 2 | 2 | 3 | 3 | 2 | 3 | 3 | 2 | 3 | 3 | 3 | 3 | 3 | 3 | 3 | 3 | 3 | 2 | 3 | 3 | 3 | 3 | 3 |
| 123975 | 2 | 2 | 2 | 3 | 3 | 2 | 2 | 2 | 3 | 2 | 2 | 2 | 3 | 3 | 2 | 3 | 3 | 2 | 3 | 3 | 3 | 3 | 3 | 3 | 3 | 3 | 3 | 1 | 3 | 3 | 3 | 3 | 3 |
| 123975 | 3 | 0 | 0 | 3 | 3 | 0 | 2 | 1 | 3 | 2 | 2 | 2 | 3 | 3 | 2 | 3 | 3 | 2 | 3 | 3 | 3 | 4 | 3 | 3 | 3 | 3 | 3 | 1 | 3 | 3 | 3 | 3 | 3 |
| 123975 | 4 | 2 | 1 | 3 | 3 | 0 | 2 | 2 | 3 | 2 | 2 | 2 | 3 | 3 | 2 | 3 | 3 | 2 | 3 | 3 | 3 | 3 | 3 | 3 | 3 | 3 | 3 | 2 | 3 | 3 | 3 | 3 | 3 |
| 123975 | 5 | 1 | 1 | 3 | 3 | 2 | 2 | 2 | 3 | 2 | 2 | 2 | 3 | 3 | 2 | 3 | 3 | 1 | 3 | 3 | 3 | 3 | 3 | 3 | 3 | 3 | 3 | 1 | 3 | 3 | 3 | 3 | 3 |
| 126414 | 1 | 3 | 3 | 1 | 3 | 3 | 3 | 3 | 3 | 3 | 3 | 3 | 3 | 3 | 3 | 3 | 3 | 3 | 2 | 2 | 3 | 3 | 3 | 3 | 3 | 3 | 3 | 2 | 3 | 3 | 3 | 3 | 3 |
| 126414 | 2 | 1 | 1 | 1 | 1 | 1 | 1 | 2 | 2 | 2 | 1 | 2 | 1 | 2 | 2 | 1 | 1 | 2 | 1 | 1 | 2 | 1 | 2 | 2 | 2 | 2 | 1 | 2 | 2 | 3 | 2 | 2 | 1 |
| 126414 | 3 | 2 | 0 | 2 | 2 | 2 | 2 | 2 | 2 | 2 | 0 | 2 | 1 | 2 | 2 | 2 | 2 | 2 | 1 | 2 | 2 | 1 | 2 | 2 | 2 | 2 | 1 | 1 | 2 | 3 | 2 | 2 | 2 |
| 126414 | 4 | 2 | 2 | 2 | 2 | 2 | 2 | 2 | 2 | 2 | 2 | 2 | 2 | 2 | 1 | 2 | 2 | 2 | 1 | 2 | 2 | 2 | 1 | 2 | 2 | 2 | 2 | 2 | 2 | 3 | 2 | 2 | 2 |
| 126414 | 5 | 3 | 3 | 2 | 3 | 3 | 3 | 3 | 3 | 3 | 3 | 3 | 3 | 3 | 3 | 3 | 3 | 3 | 2 | 2 | 3 | 3 | 3 | 3 | 3 | 3 | 3 | 2 | 3 | 4 | 3 | 3 | 3 |
| 126433 | 1 | 4 | 2 | 2 | 4 | 4 | 2 | 2 | 2 | 3 | 3 | 4 | 4 | 2 | 4 | 2 | 2 | 4 | 4 | 2 | 4 | 3 | 3 | 3 | 3 | 2 | 2 | 2 | 3 | 4 | 3 | 3 | 3 |
| 126433 | 2 | 3 | 2 | 2 | 3 | 3 | 2 | 2 | 2 | 3 | 3 | 4 | 3 | 2 | 3 | 2 | 2 | 3 | 4 | 2 | 4 | 3 | 4 | 3 | 3 | 2 | 2 | 2 | 3 | 3 | 3 | 3 | 3 |
| 126433 | 3 | 3 | 2 | 2 | 3 | 4 | 2 | 2 | 2 | 4 | 4 | 3 | 4 | 2 | 3 | 2 | 2 | 3 | 4 | 2 | 4 | 4 | 4 | 3 | 3 | 2 | 2 | 2 | 3 | 3 | 3 | 3 | 3 |
| 126433 | 4 | 3 | 2 | 2 | 4 | 4 | 2 | 2 | 2 | 3 | 4 | 4 | 4 | 2 | 3 | 2 | 2 | 3 | 4 | 2 | 4 | 3 | 4 | 3 | 3 | 2 | 2 | 2 | 3 | 3 | 3 | 3 | 4 |
| 126433 | 5 | 3 | 2 | 2 | 3 | 4 | 2 | 2 | 2 | 3 | 3 | 4 | 4 | 2 | 3 | 2 | 2 | 3 | 4 | 2 | 4 | 3 | 4 | 4 | 3 | 2 | 2 | 2 | 3 | 3 | 3 | 3 | 3 |
| 131685 | 1 | 2 | 2 | 2 | 3 | 2 | 4 | 2 | 3 | 3 | 3 | 3 | 3 | 3 | 3 | 3 | 3 | 2 | 3 | 3 | 4 | 3 | 3 | 3 | 3 | 3 | 3 | 1 | 3 | 3 | 3 | 3 | 3 |
| 131685 | 2 | 2 | 2 | 2 | 3 | 2 | 3 | 2 | 3 | 3 | 3 | 4 | 3 | 4 | 3 | 4 | 3 | 2 | 4 | 3 | 4 | 3 | 3 | 4 | 3 | 3 | 3 | 2 | 3 | 3 | 3 | 3 | 3 |
| 131685 | 3 | 2 | 2 | 2 | 3 | 2 | 3 | 2 | 3 | 3 | 3 | 3 | 3 | 3 | 3 | 3 | 3 | 2 | 3 | 3 | 3 | 3 | 3 | 3 | 3 | 3 | 3 | 2 | 3 | 3 | 3 | 3 | 3 |
| 131685 | 4 | 2 | 2 | 1 | 3 | 2 | 3 | 2 | 3 | 3 | 3 | 3 | 3 | 3 | 3 | 3 | 3 | 1 | 3 | 3 | 3 | 3 | 3 | 3 | 3 | 3 | 3 | 2 | 3 | 3 | 3 | 3 | 3 |
| 131685 | 5 | 2 | 2 | 2 | 3 | 2 | 3 | 2 | 3 | 3 | 3 | 3 | 3 | 3 | 3 | 3 | 3 | 1 | 3 | 3 | 3 | 3 | 3 | 3 | 3 | 3 | 3 | 2 | 3 | 3 | 3 | 3 | 3 |
| 135239 | 1 | 3 | 3 | 0 | 3 | 3 | 3 | 4 | 3 | 3 | 3 | 3 | 4 | 3 | 3 | 3 | 3 | 2 | 3 | 4 | 3 | 3 | 3 | 3 | 3 | 3 | 3 | 3 | 3 | 3 | 3 | 3 | 3 |
| 135239 | 2 | 3 | 3 | 0 | 3 | 3 | 3 | 4 | 3 | 3 | 3 | 3 | 4 | 3 | 3 | 3 | 3 | 2 | 3 | 3 | 3 | 3 | 3 | 3 | 3 | 3 | 3 | 3 | 3 | 3 | 3 | 3 | 3 |
| 135239 | 3 | 3 | 3 | 0 | 4 | 4 | 4 | 4 | 3 | 3 | 3 | 3 | 4 | 3 | 3 | 3 | 3 | 2 | 3 | 3 | 3 | 3 | 3 | 3 | 3 | 3 | 3 | 3 | 3 | 4 | 3 | 3 | 3 |
| 135239 | 4 | 3 | 3 | 0 | 3 | 3 | 3 | 4 | 3 | 2 | 3 | 3 | 4 | 3 | 3 | 3 | 3 | 2 | 3 | 3 | 3 | 3 | 4 | 3 | 3 | 3 | 3 | 3 | 3 | 3 | 3 | 4 | 3 |
| 135239 | 5 | 3 | 3 | 2 | 4 | 4 | 3 | 3 | 3 | 3 | 3 | 3 | 3 | 3 | 3 | 4 | 4 | 2 | 3 | 3 | 3 | 4 | 3 | 3 | 3 | 4 | 3 | 4 | 3 | 3 | 3 | 3 | 3 |
| 135244 | 1 | 3 | 3 | 1 | 0 | 3 | 1 | 2 | 3 | 0 | 1 | 3 | 3 | 3 | 3 | 2 | 3 | 3 | 3 | 3 | 2 | 1 | 3 | 3 | 2 | 1 | 2 | 1 | 3 | 3 | 3 | 3 | 3 |
| 135244 | 2 | 3 | 3 | 0 | 0 | 3 | 1 | 1 | 3 | 1 | 1 | 3 | 3 | 3 | 3 | 1 | 3 | 3 | 3 | 3 | 2 | 1 | 3 | 3 | 2 | 1 | 2 | 2 | 3 | 3 | 3 | 3 | 3 |
| 135249 | 1 | 1 | 2 | 1 | 2 | 2 | 3 | 3 | 3 | 2 | 2 | 3 | 3 | 3 | 3 | 3 | 2 | 2 | 2 | 3 | 3 | 1 | 3 | 3 | 2 | 3 | 2 | 3 | 3 | 3 | 3 | 3 | 3 |
| 135249 | 2 | 2 | 2 | 0 | 2 | 2 | 2 | 2 | 2 | 2 | 2 | 2 | 2 | 2 | 2 | 2 | 2 | 2 | 2 | 2 | 2 | 2 | 2 | 1 | 1 | 2 | 2 | 2 | 2 | 2 | 2 | 2 | 2 |
| 135249 | 3 | 2 | 2 | 1 | 2 | 2 | 3 | 3 | 3 | 2 | 2 | 3 | 3 | 3 | 3 | 3 | 2 | 2 | 2 | 3 | 3 | 2 | 3 | 3 | 2 | 3 | 2 | 3 | 3 | 3 | 3 | 3 | 3 |
| 135249 | 4 | 2 | 2 | 1 | 2 | 2 | 3 | 3 | 3 | 2 | 2 | 3 | 3 | 3 | 3 | 3 | 2 | 2 | 2 | 3 | 3 | 2 | 3 | 3 | 2 | 3 | 2 | 3 | 3 | 3 | 3 | 3 | 3 |
| 135249 | 5 | 2 | 2 | 1 | 2 | 2 | 3 | 3 | 3 | 2 | 2 | 3 | 3 | 3 | 3 | 3 | 2 | 2 | 2 | 3 | 3 | 2 | 3 | 3 | 2 | 3 | 2 | 3 | 3 | 3 | 3 | 3 | 3 |
| 135270 | 1 | 2 | 3 | 2 | 3 | 3 | 3 | 3 | 3 | 2 | 3 | 3 | 3 | 3 | 3 | 3 | 3 | 3 | 3 | 3 | 3 | 2 | 3 | 3 | 3 | 3 | 3 | 3 | 4 | 3 | 3 | 3 | 3 |
| 135270 | 2 | 2 | 3 | 0 | 3 | 3 | 3 | 3 | 3 | 2 | 3 | 3 | 3 | 3 | 3 | 3 | 3 | 3 | 3 | 3 | 3 | 2 | 3 | 3 | 3 | 3 | 3 | 3 | 3 | 3 | 3 | 3 | 3 |
| 135270 | 3 | 2 | 2 | 2 | 3 | 3 | 3 | 3 | 2 | 2 | 3 | 3 | 3 | 3 | 3 | 3 | 3 | 2 | 3 | 3 | 3 | 2 | 3 | 3 | 3 | 3 | 2 | 2 | 3 | 3 | 3 | 3 | 3 |
| 135270 | 4 | 2 | 1 | 2 | 3 | 3 | 3 | 3 | 2 | 2 | 3 | 3 | 3 | 3 | 3 | 3 | 3 | 2 | 3 | 3 | 3 | 2 | 3 | 3 | 3 | 3 | 1 | 2 | 3 | 3 | 3 | 3 | 3 |
| 135270 | 5 | 2 | 3 | 0 | 3 | 3 | 3 | 3 | 3 | 2 | 3 | 3 | 3 | 3 | 3 | 3 | 3 | 3 | 3 | 3 | 3 | 2 | 3 | 3 | 3 | 3 | 3 | 3 | 3 | 3 | 3 | 3 | 3 |
| 135279 | 1 | 2 | 2 | 3 | 2 | 3 | 3 | 3 | 2 | 2 | 3 | 3 | 3 | 3 | 3 | 2 | 3 | 2 | 2 | 3 | 2 | 3 | 3 | 3 | 2 | 3 | 3 | 3 | 3 | 2 | 2 | 3 | 4 |
| 135279 | 2 | 1 | 1 | 0 | 1 | 1 | 3 | 1 | 2 | 1 | 3 | 1 | 3 | 2 | 2 | 2 | 1 | 1 | 1 | 2 | 2 | 3 | 2 | 1 | 1 | 3 | 3 | 1 | 2 | 3 | 2 | 3 | 2 |
| 135279 | 3 | 2 | 3 | 2 | 3 | 3 | 3 | 3 | 2 | 2 | 3 | 2 | 3 | 3 | 3 | 3 | 2 | 2 | 3 | 3 | 3 | 3 | 3 | 3 | 3 | 3 | 3 | 3 | 3 | 3 | 3 | 3 | 4 |
| 135279 | 4 | 3 | 4 | 4 | 4 | 4 | 4 | 4 | 4 | 4 | 4 | 4 | 3 | 4 | 3 | 4 | 3 | 4 | 3 | 4 | 3 | 3 | 3 | 3 | 3 | 3 | 3 | 3 | 4 | 3 | 3 | 3 | 4 |
| 135279 | 5 | 3 | 4 | 4 | 4 | 4 | 4 | 4 | 4 | 3 | 4 | 4 | 3 | 4 | 4 | 4 | 3 | 4 | 3 | 4 | 3 | 3 | 4 | 4 | 3 | 3 | 4 | 3 | 4 | 3 | 3 | 4 | 4 |
| 135294 | 1 | 3 | 3 | 3 | 3 | 4 | 3 | 4 | 3 | 3 | 3 | 3 | 3 | 3 | 3 | 3 | 3 | 3 | 3 | 3 | 3 | 3 | 3 | 4 | 3 | 3 | 3 | 3 | 4 | 3 | 3 | 3 | 3 |
| 135294 | 2 | 2 | 2 | 4 | 3 | 3 | 3 | 2 | 2 | 3 | 3 | 3 | 3 | 3 | 2 | 3 | 3 | 2 | 3 | 3 | 3 | 3 | 3 | 3 | 2 | 3 | 3 | 3 | 2 | 3 | 2 | 2 | 3 |
| 135294 | 3 | 3 | 3 | 3 | 3 | 3 | 3 | 3 | 3 | 3 | 3 | 3 | 3 | 4 | 3 | 3 | 3 | 3 | 3 | 3 | 3 | 3 | 3 | 3 | 3 | 3 | 3 | 4 | 3 | 3 | 3 | 3 | 3 |
| 135294 | 4 | 1 | 2 | 3 | 2 | 3 | 2 | 3 | 3 | 2 | 2 | 2 | 2 | 3 | 3 | 3 | 2 | 2 | 3 | 3 | 3 | 2 | 3 | 3 | 3 | 2 | 3 | 2 | 3 | 3 | 3 | 3 | 3 |
| 135294 | 5 | 3 | 2 | 3 | 2 | 3 | 3 | 3 | 3 | 3 | 2 | 3 | 3 | 3 | 3 | 3 | 2 | 2 | 3 | 3 | 3 | 2 | 3 | 3 | 3 | 3 | 3 | 3 | 3 | 3 | 2 | 3 | 3 |
| 135333 | 1 | 3 | 2 | 1 | 3 | 1 | 2 | 3 | 3 | 3 | 3 | 3 | 3 | 3 | 3 | 3 | 3 | 3 | 3 | 3 | 3 | 3 | 3 | 3 | 3 | 3 | 3 | 3 | 3 | 2 | 2 | 3 | 3 |
| 135333 | 2 | 3 | 2 | 1 | 3 | 1 | 2 | 3 | 3 | 3 | 3 | 3 | 3 | 3 | 3 | 3 | 3 | 3 | 3 | 3 | 3 | 3 | 3 | 3 | 3 | 4 | 3 | 3 | 3 | 2 | 2 | 3 | 3 |
| 135333 | 3 | 2 | 2 | 0 | 2 | 0 | 3 | 3 | 3 | 2 | 2 | 2 | 2 | 2 | 2 | 2 | 3 | 2 | 3 | 3 | 3 | 3 | 3 | 3 | 2 | 3 | 3 | 2 | 4 | 3 | 2 | 3 | 3 |
| 135333 | 4 | 2 | 2 | 1 | 3 | 3 | 2 | 2 | 3 | 2 | 3 | 2 | 3 | 2 | 2 | 3 | 2 | 2 | 2 | 3 | 2 | 3 | 2 | 3 | 3 | 3 | 3 | 2 | 2 | 3 | 2 | 1 | 2 |
| 135333 | 5 | 2 | 1 | 2 | 0 | 3 | 3 | 0 | 2 | 3 | 4 | 3 | 4 | 4 | 2 | 0 | 4 | 1 | 3 | 4 | 3 | 0 | 3 | 3 | 3 | 2 | 3 | 4 | 3 | 4 | 3 | 3 | 4 |
| 135351 | 1 | 2 | 2 | 4 | 3 | 2 | 4 | 2 | 3 | 3 | 2 | 3 | 3 | 4 | 3 | 3 | 3 | 2 | 3 | 3 | 3 | 3 | 3 | 3 | 3 | 3 | 3 | 2 | 3 | 3 | 3 | 3 | 2 |
| 135351 | 2 | 2 | 2 | 3 | 3 | 2 | 3 | 2 | 3 | 3 | 2 | 3 | 3 | 3 | 3 | 3 | 4 | 2 | 3 | 3 | 3 | 3 | 3 | 3 | 3 | 3 | 3 | 2 | 3 | 3 | 3 | 3 | 2 |
| 135351 | 3 | 2 | 2 | 4 | 3 | 2 | 4 | 2 | 3 | 3 | 2 | 3 | 3 | 3 | 3 | 3 | 4 | 2 | 3 | 3 | 3 | 3 | 3 | 3 | 3 | 3 | 3 | 2 | 3 | 3 | 3 | 3 | 2 |
| 135351 | 4 | 2 | 2 | 4 | 3 | 2 | 4 | 2 | 3 | 3 | 2 | 3 | 3 | 3 | 3 | 3 | 4 | 2 | 3 | 3 | 3 | 3 | 3 | 3 | 3 | 3 | 3 | 2 | 3 | 3 | 3 | 3 | 2 |
| 135351 | 5 | 2 | 2 | 3 | 3 | 2 | 4 | 2 | 3 | 3 | 2 | 3 | 3 | 3 | 3 | 3 | 3 | 2 | 3 | 3 | 3 | 3 | 3 | 3 | 3 | 3 | 3 | 2 | 3 | 3 | 3 | 3 | 2 |
| 135461 | 1 | 2 | 3 | 3 | 2 | 2 | 2 | 3 | 1 | 2 | 2 | 1 | 2 | 1 | 3 | 3 | 3 | 3 | 2 | 1 | 1 | 2 | 3 | 3 | 2 | 2 | 2 | 2 | 3 | 2 | 2 | 1 | 1 |
| 135461 | 2 | 2 | 3 | 3 | 2 | 2 | 2 | 3 | 2 | 2 | 2 | 2 | 2 | 2 | 3 | 3 | 3 | 3 | 2 | 2 | 1 | 2 | 3 | 3 | 2 | 2 | 2 | 2 | 3 | 2 | 2 | 2 | 2 |
| 135461 | 3 | 2 | 3 | 3 | 2 | 2 | 2 | 3 | 2 | 2 | 2 | 2 | 2 | 2 | 3 | 3 | 3 | 3 | 2 | 2 | 2 | 2 | 3 | 3 | 2 | 2 | 2 | 2 | 3 | 2 | 2 | 2 | 2 |
| 135461 | 4 | 2 | 3 | 3 | 2 | 2 | 2 | 3 | 2 | 2 | 2 | 2 | 2 | 2 | 3 | 3 | 3 | 3 | 2 | 2 | 2 | 2 | 3 | 3 | 2 | 2 | 2 | 2 | 3 | 2 | 2 | 1 | 1 |
| 135461 | 5 | 2 | 3 | 3 | 2 | 1 | 2 | 3 | 2 | 2 | 2 | 2 | 2 | 2 | 3 | 3 | 3 | 3 | 2 | 1 | 2 | 2 | 3 | 3 | 2 | 2 | 2 | 2 | 3 | 2 | 2 | 1 | 2 |
| 135474 | 1 | 3 | 3 | 2 | 3 | 3 | 3 | 3 | 3 | 3 | 3 | 3 | 3 | 3 | 3 | 3 | 3 | 3 | 3 | 3 | 3 | 3 | 3 | 3 | 3 | 3 | 3 | 3 | 3 | 3 | 2 | 1 | 2 |
| 135474 | 2 | 3 | 3 | 0 | 3 | 3 | 3 | 3 | 3 | 3 | 3 | 3 | 3 | 3 | 3 | 3 | 3 | 3 | 3 | 3 | 3 | 3 | 3 | 3 | 3 | 3 | 3 | 3 | 3 | 3 | 2 | 2 | 2 |
| 135474 | 3 | 3 | 3 | 2 | 3 | 3 | 3 | 3 | 3 | 3 | 3 | 3 | 3 | 3 | 3 | 3 | 3 | 3 | 3 | 3 | 3 | 3 | 3 | 3 | 3 | 3 | 3 | 3 | 3 | 3 | 1 | 2 | 2 |
| 135474 | 4 | 3 | 3 | 2 | 3 | 3 | 3 | 3 | 3 | 3 | 3 | 3 | 3 | 3 | 3 | 3 | 3 | 3 | 3 | 3 | 3 | 3 | 3 | 3 | 3 | 3 | 3 | 3 | 3 | 3 | 2 | 2 | 2 |
| 135474 | 5 | 3 | 3 | 2 | 3 | 3 | 3 | 3 | 3 | 3 | 3 | 3 | 3 | 3 | 3 | 3 | 3 | 3 | 3 | 3 | 3 | 3 | 3 | 3 | 3 | 3 | 3 | 3 | 3 | 3 | 2 | 1 | 2 |
| 135489 | 1 | 1 | 2 | 2 | 2 | 2 | 2 | 2 | 2 | 2 | 2 | 2 | 2 | 2 | 2 | 2 | 2 | 2 | 1 | 2 | 2 | 1 | 2 | 2 | 2 | 2 | 2 | 2 | 2 | 2 | 1 | 1 | 2 |
| 135489 | 2 | 3 | 3 | 3 | 3 | 3 | 3 | 3 | 3 | 3 | 3 | 3 | 3 | 3 | 3 | 3 | 3 | 3 | 2 | 3 | 3 | 3 | 3 | 3 | 3 | 3 | 3 | 3 | 3 | 3 | 2 | 2 | 3 |
| 135489 | 3 | 3 | 2 | 2 | 3 | 2 | 2 | 3 | 3 | 3 | 2 | 3 | 3 | 3 | 3 | 3 | 3 | 2 | 2 | 2 | 3 | 3 | 3 | 3 | 3 | 3 | 2 | 3 | 3 | 3 | 2 | 1 | 2 |
| 135489 | 4 | 3 | 2 | 2 | 3 | 2 | 2 | 3 | 3 | 3 | 2 | 3 | 3 | 3 | 3 | 3 | 3 | 2 | 2 | 2 | 3 | 3 | 3 | 3 | 3 | 3 | 2 | 3 | 3 | 3 | 1 | 1 | 2 |
| 135489 | 5 | 3 | 3 | 3 | 3 | 3 | 3 | 3 | 3 | 3 | 3 | 3 | 3 | 3 | 3 | 3 | 3 | 3 | 2 | 3 | 3 | 3 | 3 | 3 | 3 | 3 | 3 | 3 | 3 | 3 | 2 | 2 | 3 |
| 135536 | 1 | 1 | 1 | 1 | 1 | 1 | 3 | 1 | 2 | 2 | 1 | 2 | 2 | 2 | 2 | 2 | 2 | 1 | 2 | 2 | 3 | 2 | 1 | 2 | 2 | 2 | 2 | 2 | 2 | 2 | 1 | 1 | 2 |
| 135536 | 2 | 3 | 1 | 2 | 3 | 2 | 2 | 2 | 2 | 3 | 3 | 3 | 3 | 2 | 2 | 2 | 2 | 3 | 3 | 2 | 3 | 2 | 3 | 3 | 3 | 2 | 2 | 2 | 2 | 2 | 1 | 2 | 2 |
| 135536 | 3 | 3 | 1 | 2 | 3 | 2 | 2 | 2 | 2 | 3 | 3 | 3 | 3 | 2 | 2 | 2 | 2 | 3 | 3 | 2 | 3 | 2 | 3 | 3 | 3 | 2 | 2 | 2 | 2 | 2 | 2 | 2 | 2 |
| 135536 | 4 | 2 | 1 | 1 | 1 | 1 | 2 | 1 | 2 | 2 | 1 | 2 | 2 | 2 | 2 | 2 | 2 | 1 | 2 | 2 | 2 | 2 | 1 | 2 | 2 | 2 | 2 | 2 | 2 | 2 | 1 | 2 | 2 |
| 135536 | 5 | 3 | 1 | 2 | 3 | 2 | 2 | 2 | 2 | 3 | 3 | 3 | 3 | 2 | 2 | 2 | 2 | 3 | 3 | 2 | 3 | 2 | 3 | 3 | 3 | 2 | 2 | 2 | 2 | 2 | 2 | 2 | 2 |
| 135553 | 1 | 2 | 2 | 3 | 2 | 2 | 3 | 2 | 3 | 2 | 2 | 2 | 3 | 2 | 2 | 3 | 2 | 2 | 2 | 2 | 2 | 2 | 2 | 3 | 2 | 3 | 2 | 2 | 2 | 2 | 2 | 2 | 2 |
| 135553 | 2 | 2 | 2 | 3 | 2 | 2 | 3 | 1 | 3 | 2 | 2 | 2 | 3 | 1 | 2 | 3 | 2 | 1 | 1 | 2 | 2 | 2 | 2 | 3 | 1 | 3 | 2 | 2 | 2 | 2 | 2 | 1 | 2 |
| 135553 | 3 | 2 | 2 | 2 | 1 | 2 | 2 | 2 | 2 | 2 | 1 | 1 | 2 | 1 | 2 | 2 | 2 | 1 | 1 | 2 | 2 | 2 | 2 | 2 | 2 | 2 | 2 | 2 | 2 | 2 | 1 | 1 | 1 |
| 135553 | 4 | 2 | 2 | 3 | 2 | 2 | 3 | 2 | 3 | 0 | 2 | 2 | 3 | 2 | 2 | 3 | 2 | 2 | 2 | 2 | 2 | 2 | 2 | 3 | 2 | 3 | 2 | 2 | 2 | 2 | 2 | 2 | 2 |
| 135553 | 5 | 2 | 1 | 3 | 2 | 2 | 3 | 2 | 3 | 2 | 2 | 2 | 3 | 2 | 2 | 3 | 2 | 2 | 2 | 2 | 2 | 2 | 1 | 3 | 2 | 3 | 2 | 2 | 2 | 2 | 1 | 2 | 2 |
| 135682 | 1 | 1 | 0 | 0 | 0 | 0 | 2 | 0 | 1 | 1 | 2 | 1 | 2 | 1 | 1 | 1 | 1 | 1 | 1 | 2 | 1 | 2 | 2 | 1 | 0 | 2 | 0 | 0 | 1 | 2 | 1 | 2 | 1 |
| 135682 | 2 | 2 | 3 | 2 | 2 | 3 | 2 | 1 | 1 | 1 | 2 | 3 | 3 | 4 | 3 | 3 | 3 | 2 | 1 | 3 | 2 | 3 | 3 | 2 | 2 | 2 | 1 | 3 | 3 | 2 | 3 | 3 | 3 |
| 135682 | 3 | 2 | 3 | 2 | 2 | 3 | 2 | 2 | 2 | 1 | 2 | 3 | 3 | 3 | 3 | 3 | 3 | 2 | 1 | 3 | 2 | 3 | 3 | 1 | 2 | 2 | 1 | 3 | 3 | 2 | 3 | 3 | 3 |
| 135682 | 4 | 2 | 3 | 2 | 2 | 3 | 2 | 1 | 2 | 2 | 2 | 3 | 3 | 3 | 3 | 3 | 3 | 2 | 2 | 3 | 2 | 3 | 3 | 2 | 2 | 2 | 2 | 3 | 3 | 2 | 3 | 3 | 4 |
| 135682 | 5 | 1 | 1 | 0 | 1 | 1 | 2 | 1 | 1 | 1 | 3 | 1 | 3 | 1 | 2 | 0 | 1 | 1 | 1 | 3 | 1 | 3 | 2 | 0 | 1 | 3 | 3 | 1 | 1 | 2 | 1 | 2 | 1 |
| 135762 | 1 | 3 | 3 | 3 | 3 | 3 | 3 | 2 | 4 | 2 | 3 | 3 | 2 | 3 | 3 | 2 | 3 | 3 | 2 | 3 | 3 | 3 | 3 | 2 | 3 | 3 | 0 | 3 | 3 | 3 | 3 | 2 | 3 |
| 135762 | 2 | 1 | 1 | 2 | 1 | 1 | 2 | 1 | 2 | 1 | 0 | 2 | 2 | 1 | 2 | 1 | 1 | 1 | 2 | 1 | 2 | 1 | 1 | 2 | 1 | 2 | 2 | 2 | 3 | 3 | 2 | 3 | 3 |
| 135777 | 1 | 2 | 3 | 2 | 3 | 2 | 3 | 1 | 3 | 2 | 3 | 3 | 3 | 3 | 3 | 2 | 1 | 1 | 1 | 3 | 1 | 2 | 3 | 1 | 3 | 3 | 1 | 1 | 2 | 3 | 2 | 3 | 2 |
| 135777 | 2 | 1 | 0 | 1 | 0 | 3 | 2 | 1 | 3 | 2 | 2 | 3 | 1 | 3 | 3 | 2 | 2 | 2 | 1 | 2 | 2 | 1 | 3 | 3 | 3 | 2 | 0 | 2 | 2 | 2 | 1 | 2 | 2 |
| 135777 | 3 | 2 | 3 | 2 | 3 | 2 | 3 | 2 | 3 | 2 | 3 | 3 | 3 | 3 | 3 | 2 | 2 | 2 | 1 | 3 | 2 | 1 | 3 | 2 | 3 | 3 | 2 | 2 | 2 | 3 | 2 | 3 | 2 |
| 135777 | 4 | 1 | 2 | 0 | 2 | 2 | 2 | 0 | 2 | 2 | 2 | 3 | 3 | 3 | 2 | 2 | 3 | 2 | 1 | 2 | 2 | 1 | 3 | 2 | 2 | 2 | 1 | 2 | 2 | 2 | 1 | 3 | 2 |
| 135777 | 5 | 2 | 3 | 1 | 3 | 2 | 3 | 1 | 3 | 2 | 3 | 3 | 3 | 3 | 3 | 2 | 2 | 2 | 2 | 3 | 2 | 2 | 3 | 0 | 3 | 3 | 1 | 2 | 2 | 3 | 2 | 3 | 2 |
| 135808 | 1 | 3 | 3 | 2 | 3 | 3 | 3 | 3 | 3 | 2 | 3 | 3 | 3 | 4 | 4 | 3 | 3 | 3 | 3 | 3 | 3 | 3 | 3 | 3 | 3 | 3 | 2 | 3 | 3 | 3 | 3 | 3 | 3 |
| 135808 | 2 | 2 | 3 | 2 | 2 | 3 | 2 | 2 | 2 | 2 | 2 | 3 | 3 | 3 | 2 | 2 | 2 | 2 | 2 | 3 | 3 | 2 | 3 | 2 | 2 | 2 | 2 | 3 | 3 | 3 | 2 | 3 | 2 |
| 135808 | 3 | 3 | 3 | 2 | 3 | 3 | 3 | 3 | 3 | 2 | 3 | 3 | 3 | 3 | 3 | 3 | 3 | 3 | 3 | 3 | 3 | 3 | 3 | 3 | 3 | 3 | 2 | 3 | 3 | 3 | 3 | 3 | 3 |
| 135808 | 4 | 2 | 3 | 2 | 2 | 3 | 2 | 2 | 2 | 2 | 2 | 3 | 3 | 3 | 2 | 2 | 2 | 2 | 2 | 3 | 3 | 2 | 3 | 2 | 2 | 2 | 2 | 3 | 3 | 3 | 2 | 3 | 2 |
| 135808 | 5 | 2 | 3 | 2 | 2 | 3 | 2 | 2 | 2 | 2 | 2 | 3 | 3 | 3 | 2 | 2 | 2 | 2 | 2 | 3 | 3 | 2 | 3 | 2 | 2 | 2 | 2 | 3 | 3 | 3 | 2 | 3 | 2 |
| 135897 | 1 | 2 | 2 | 1 | 1 | 2 | 2 | 1 | 1 | 2 | 3 | 3 | 2 | 2 | 2 | 3 | 3 | 1 | 1 | 3 | 2 | 2 | 3 | 1 | 2 | 2 | 1 | 3 | 3 | 3 | 3 | 3 | 3 |
| 135897 | 2 | 2 | 2 | 1 | 1 | 2 | 2 | 1 | 1 | 2 | 2 | 2 | 2 | 2 | 2 | 2 | 2 | 1 | 2 | 2 | 2 | 2 | 3 | 2 | 2 | 2 | 2 | 3 | 3 | 3 | 3 | 3 | 3 |
| 135897 | 3 | 3 | 4 | 0 | 3 | 2 | 3 | 4 | 3 | 3 | 3 | 3 | 3 | 3 | 3 | 3 | 3 | 2 | 1 | 3 | 3 | 3 | 3 | 3 | 4 | 3 | 3 | 3 | 3 | 3 | 2 | 3 | 3 |
| 135897 | 4 | 2 | 2 | 1 | 1 | 2 | 2 | 1 | 2 | 2 | 3 | 3 | 2 | 2 | 2 | 3 | 3 | 1 | 1 | 3 | 2 | 2 | 3 | 2 | 2 | 2 | 2 | 3 | 3 | 3 | 3 | 3 | 3 |
| 135897 | 5 | 2 | 2 | 1 | 1 | 2 | 2 | 0 | 2 | 2 | 1 | 2 | 2 | 2 | 2 | 2 | 2 | 2 | 2 | 2 | 2 | 2 | 3 | 1 | 2 | 2 | 2 | 3 | 3 | 3 | 3 | 3 | 3 |

*Origin and virulence of isolates in the given order are shown in Table 2.

†Response types 0–4, where 0 = resistant and 4 = susceptible (Torp et al. 1978).
